# Direct pharmacological targeting of asparagine synthetase to overcome resistance to L-asparaginase in ALL therapy

**DOI:** 10.1172/jci.insight.203777

**Published:** 2026-06-04

**Authors:** Rodney Claude, Sankalp Srivastava, Kirk A. Staschke, Carlos Mellado-Fritz, Shaoxiong Chen, Lei Liu, Minghua Zhong, Harish Kothandaraman, Nadia A. Lanman, Utpal Davé, Sandeep Batra, Jiehao Zhou, Yue Fang, Chi Zhang, Reuben Kapur, Jing Fan, Ronald C. Wek, Ji Zhang

**Affiliations:** 1 Herman B. Wells Center for Pediatric Research,; 2 Department of Biochemistry, Molecular Biology, and Pharmacology, and; 3 Melvin and Bren Simon Comprehensive Cancer Center, Indiana University School of Medicine, Indianapolis, Indiana, USA.; 4 Morgridge Institute for Research & Department of Medical Microbiology and Immunology, University of Wisconsin-Madison, Madison, Wisconsin, USA.; 5 Department of Pathology, Indiana University School of Medicine, Indianapolis, Indiana, USA.; 6 Purdue University Institute for Cancer Research, and; 7 Department of Comparative Pathobiology, Purdue University, West Lafayette, Indiana, USA.; 8 Department of Medicine, Indiana University School of Medicine, Indianapolis, Indiana, USA.; 9 Riley Hospital for Children at Indiana University Health, Indianapolis, Indiana, USA.; 10 Department of Laboratory Medicine and Pathology, Mayo Clinic Arizona, Phoenix, Arizona, USA.; 11 Department of Medical and Molecular Genetics, Indiana University School of Medicine, Indianapolis, Indiana, USA.

**Keywords:** Hematology, Metabolism, Amino acid metabolism, Cell stress, Leukemias

## Abstract

Acute lymphoblastic leukemia (ALL) is the most common pediatric cancer, arising from both B and T cell lineages (B-ALL and T-ALL). Current therapy exploits ALL cells’ low expression of asparagine synthetase (ASNS) by using L-asparaginase, a bacterial enzyme that depletes circulating asparagine. However, resistance can emerge through induction of ASNS, mediated in part by the amino acid stress sensor GCN2. In this study, we addressed the efficacy of L-asparaginase in combination with genetic or pharmacological inhibition of GCN2 and the ASNS inhibitor ASX-173. Using a *Kras^G12D^*-driven mouse model of T-ALL, we found that GCN2 is dispensable for leukemogenesis. However, genetic inactivation or pharmacologic inhibition of GCN2 sensitized ALL cells to asparagine depletion, correlating with impaired ASNS induction. While GCN2 targeting enhanced sensitivity to asparagine depletion, a subset of *Gcn2*^–/–^ T-ALL cells retained high ASNS expression and remained resistant to L-asparaginase. Likewise, some human T-ALL cells with elevated ASNS levels were refractory to GCN2 inhibition even under asparagine-depleted conditions. When combined with L-asparaginase, ASX-173 effectively eliminated ASNS^hi^ leukemic cells in vitro and in vivo. These findings suggest that direct targeting of ASNS provides therapeutic benefit in leukemias that express high levels of ASNS and are resistant to GCN2 inhibition under asparagine-depleted conditions.

## Abstract 2

We established a strategy to prevent leukemia cells from producing their own amino acid to synergize with a traditional chemoagent.

## Graphical abstract

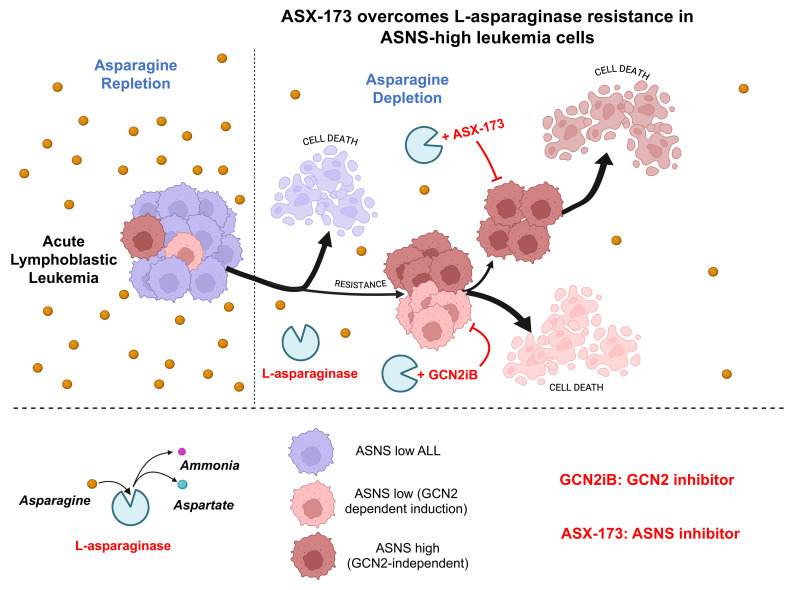

## Introduction

Acute lymphoblastic leukemia (ALL) is the most frequently diagnosed pediatric cancer, characterized by the rapid proliferation of immature B or T lymphoblasts (B-ALL and T-ALL) in the bone marrow that can disseminate to the peripheral blood and lymphoid organs such as the spleen (1, 2). Recent advances in understanding metabolic vulnerabilities in cancer have led to the development of therapeutic agents that target metabolic dependencies (3). Among these, L-asparaginase is a key component of ALL therapy, functioning by depleting circulating asparagine (4). Its efficacy relies on the observation that ALL cells express low levels of asparagine synthetase (ASNS) and therefore depend on extracellular asparagine for survival (5).

However, emerging evidence indicates that ALL cells can upregulate ASNS expression following asparagine depletion through the integrated stress response (ISR), thereby conferring resistance to L-asparaginase (6). The ISR features a family of protein kinases that phosphorylate the α subunit of eIF2, conferring adaptive gene expression. Central to the ISR are eIF2α kinases that sense cellular stresses, including GCN2 that responds to amino acid depletion and PERK that is activated by endoplasmic reticulum (ER) stress (7). The ensuing induced phosphorylation of eIF2α (p-eIF2α) leads to enhanced expression of ATF4, a transcriptional activator of genes involved in uptake, reclamation, and synthesis of amino acids, including ASNS. Furthermore, promoter demethylation of the *ASNS* gene is required for ATF4 recruitment and transcriptional activation (8). Therefore, multiple mechanisms contribute to regulation of ASNS expression.

Given that GCN2 and the ISR are critical for ASNS induction, pharmacological inhibition of GCN2 has been shown to sensitize ASNS^lo^ leukemia cells to L-asparaginase treatment (6). Interestingly, GCN2 has also been implicated in prostate cancer cell proliferation under amino acid–replete conditions, suggesting a broader role in supporting tumor growth under suboptimal nutritional microenvironment (9). Nonetheless, the contribution of GCN2 to leukemia initiation and whether GCN2-independent mechanisms may also contribute to leukemia resistance to asparagine depletion remain unclear.

Despite high cure rates, approximately 15% of pediatric ALL patients relapse and develop chemoresistance, leading to poor outcomes (10). Thus, identifying new therapeutic strategies to overcome L-asparaginase resistance is of critical importance. Notably, combining GCN2 inhibitors with L-asparaginase does not benefit cancers with high ASNS expression (6), likely because these cells can synthesize asparagine de novo and are independent of the GCN2 pathway for ASNS induction. These observations suggest that small-molecule inhibitors of ASNS would be effective in combination therapies for relapsed ALL. Although first-generation ASNS inhibitors demonstrated poor cell permeability and limited efficacy in cellular assays (11), the potential of newer ASNS inhibitors to overcome L-asparaginase resistance in ASNS-high cancers remains unexplored.

In this study, we addressed the therapeutic utility of ASX-173, a new and potent ASNS inhibitor (12). We employed a *Kras^G12D^*-driven mouse T-ALL model to investigate ASX-173 in combination with L-asparaginase and genetic and pharmacological inhibition of GCN2 to evaluate individual and combination efficacy in leukemia initiation and therapeutic resistance. Deletion of *Gcn2* did not delay leukemia onset but rendered leukemia cells sensitive to asparagine depletion. Similarly, pharmacological inhibition of GCN2 using GCN2iB sensitized wild-type (WT) leukemia cells to L-asparaginase both in vitro and in vivo. However, we identified a subset of *Gcn2^–/–^* leukemia cells that maintained high ASNS expression, indicating a GCN2-independent mechanism of ASNS regulation. Consistent with this, certain human T-ALL cell lines with elevated basal ASNS levels exhibited minimal response to GCN2iB and L-asparaginase treatment. These findings suggested features of ALL chemoresistance that we address by directly targeting ASNS using ASX-173. Combined treatment with ASX-173 and L-asparaginase demonstrated strong antileukemic efficacy in ASNS^hi^ cells. Collectively, our results establish that targeting the GCN2/ASNS axis represents a promising strategy to overcome L-asparaginase resistance in ALL.

## Results

### Genetic inactivation of GCN2 alone does not delay leukemia onset in a Kras^G12D^-driven mouse T-ALL model.

**Figure 1:**
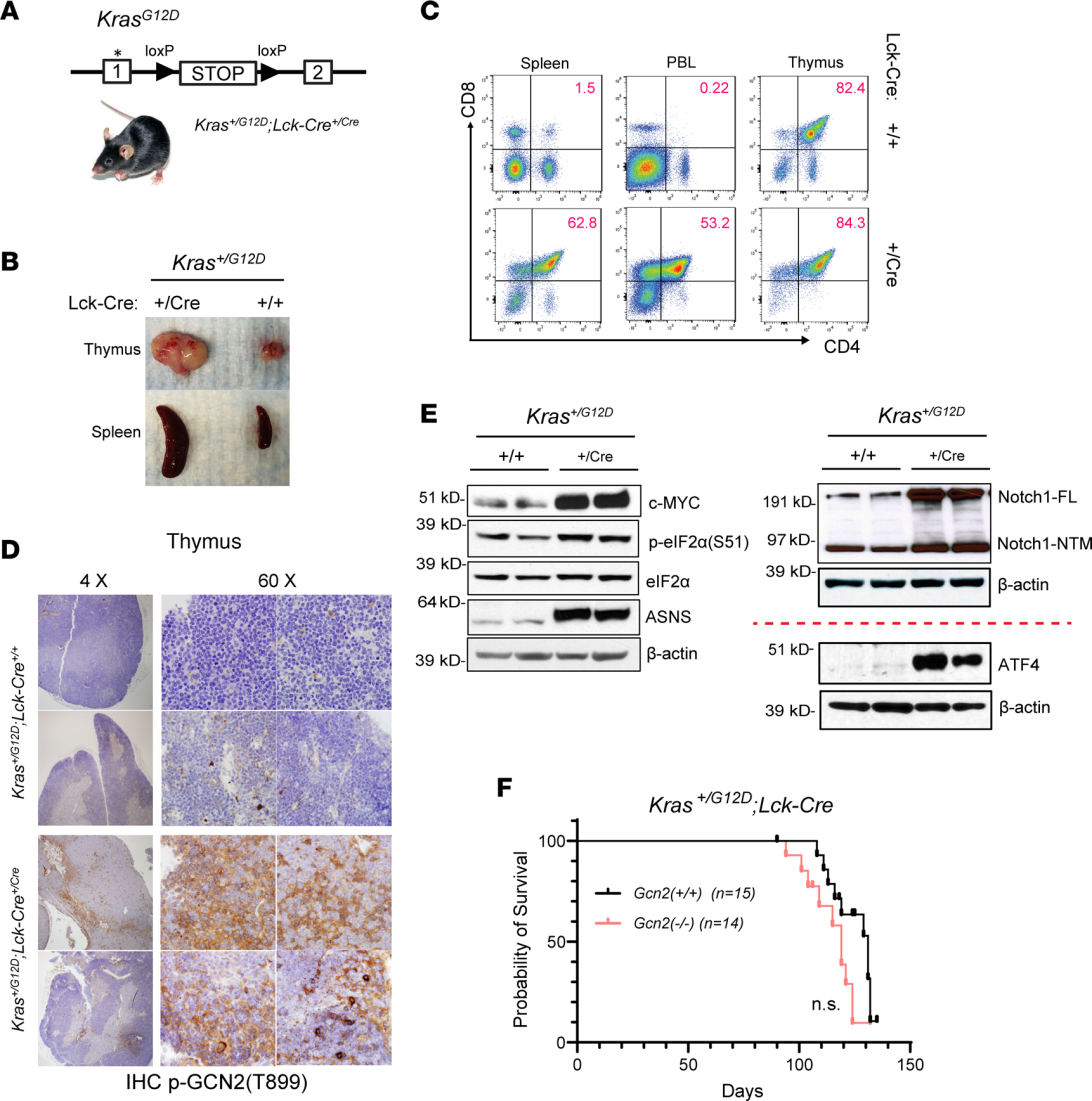
GCN2 is not required for leukemogenesis. (**A**) Schematic diagram of T-ALL mice that were established by a *Kras^G12D^* mutation knocked into the endogenous locus of the *Kras* gene. The loxP-flanked STOP codon was deleted only when Cre was expressed under the T cell–specific *Lck* promoter. (**B**) Representative images of thymus and spleen, collected at 16 weeks from *Kras^G12D^* mice with or without Cre. (**C**) CD4^+^/CD8^+^ cell profiles were assessed by flow cytometry analysis in spleen, peripheral blood (PBL), and thymus of *Kras^G12D^* mice with or without Cre. (**D**) IHC staining of p-GCN2 (T899) in thymus from *Kras^G12D^* mice with or without Cre. Images from 2 representative mice of each genotype are shown. (**E**) Western blot analysis was used to measure c-MYC, NOTCH-1, p-eIF2α (S51), ATF4, and ASNS protein in thymus from *Kras^G12D^* mice, with or without Cre. NOTCH-1 and ATF4 blots were run at a separate time, and protein lysates for the ATF4 blot were prepared from independent animals. (**F**) *Kras^G12D^;Lck-Cre* T-ALL mice were crossed with *Gcn2^–/–^* mice. Kaplan-Meier curve comparing survival between the *Gcn2^+/+^* and *Gcn2^–/–^* mice. Statistical comparison between the 2 groups was done using the log-rank (Mantel-Cox) test.

Previous studies indicate that GCN2 is activated under asparagine-depleted conditions to alleviate amino acid stress through ATF4-dependent induction of ASNS (6, 8). To address its role in ALL, we employed a mouse T-ALL model driven by Kras^G12D^ activated by a T cell–specific Cre recombinase (*Lck-Cre*) (Figure 1A) (13, 14). Mice expressing *Lck-Cre* developed aggressive T-ALL within 16 weeks with 100% penetrance, characterized by enlarged thymus and spleen (Figure 1B), and increased CD4^+^CD8^+^ double-positive cells in the peripheral blood (PBL) and spleen (Figure 1C). Immunohistochemical (IHC) staining for p-GCN2 (T899), a marker of GCN2 activation, revealed regional positivity in the thymus, suggesting a potential role for GCN2 in leukemogenesis (Figure 1D). In addition, leukemic thymus tissues exhibited elevated expression of oncogenic molecules NOTCH-1 and c-MYC (Figure 1E), both critical for T-ALL pathogenesis (15). Furthermore, we also observed increased phosphorylation of eIF2α (S51), and upregulation of ATF4 and ASNS in leukemic compared with normal thymus (Figure 1E), implicating activation of an upstream eIF2α kinase. Among the candidate kinases, PERK and GCN2 are known to contribute to amino acid stress adaptation in MYC-driven cancers (16). While PERK primarily responds to endoplasmic reticulum (ER) stress (7), its phosphorylation at Thr982 — a marker of its activation — was minimally detected in leukemic thymus tissues (Supplemental Figure 1A; supplemental material available online with this article; https://doi.org/10.1172/jci.insight.203777DS1), suggesting PERK is not a major driver of eIF2α phosphorylation in this model. These results prompted us to further evaluate the role of GCN2 in T-ALL initiation and progression.

We crossed *Kras^G12D^;Lck-Cre* mice with *Gcn2^–/–^* animals to generate GCN2-deficient T-ALL mice. *Gcn2*-germline-knockout mice develop normally unless fed on amino acid–restricted food, confirming its central role in amino acid sensing (17). Kaplan-Meier survival analysis revealed no significant difference in leukemia onset or overall survival between *Gcn2^+/+^* and *Gcn2^–/–^* T-ALL mice (Figure 1F). We did not observe differences in spleen or thymus weights, nor in splenic CD4^+^/CD8^+^ cell profiles, between genotypes (Supplemental Figure 1, B and C). Western blot analysis of thymic tissues showed minimal PERK activation only in one sample, though ASNS expression trended lower in *Gcn2^–/–^* leukemias (Supplemental Figure 1D). To determine whether this effect was intrinsic to leukemia cells, we generated a secondary T-ALL model by transplanting preleukemic bone marrow from 7-week-old *Kras^G12D^;Lck-Cre;Gcn2^+/+^* or *Gcn2^–/–^* mice into lethally irradiated WT recipients (Supplemental Figure 1E). At this preleukemic stage, donor bone marrow displayed a normal CD4^+^/CD8^+^ cell profile (Supplemental Figure 1F). This secondary model exhibited a shorter latency, yet no significant difference in leukemia onset was observed between genotypes (Supplemental Figure 1G). Control mice lacking the *Kras^G12D^* allele did not develop leukemia (Supplemental Figure 1G). Collectively, these data indicate that GCN2 is dispensable for leukemia initiation and progression in this *Kras^G12D^*-driven T-ALL model.

### GCN2 deficiency sensitizes ALL cells to asparagine depletion.

**Figure 2:**
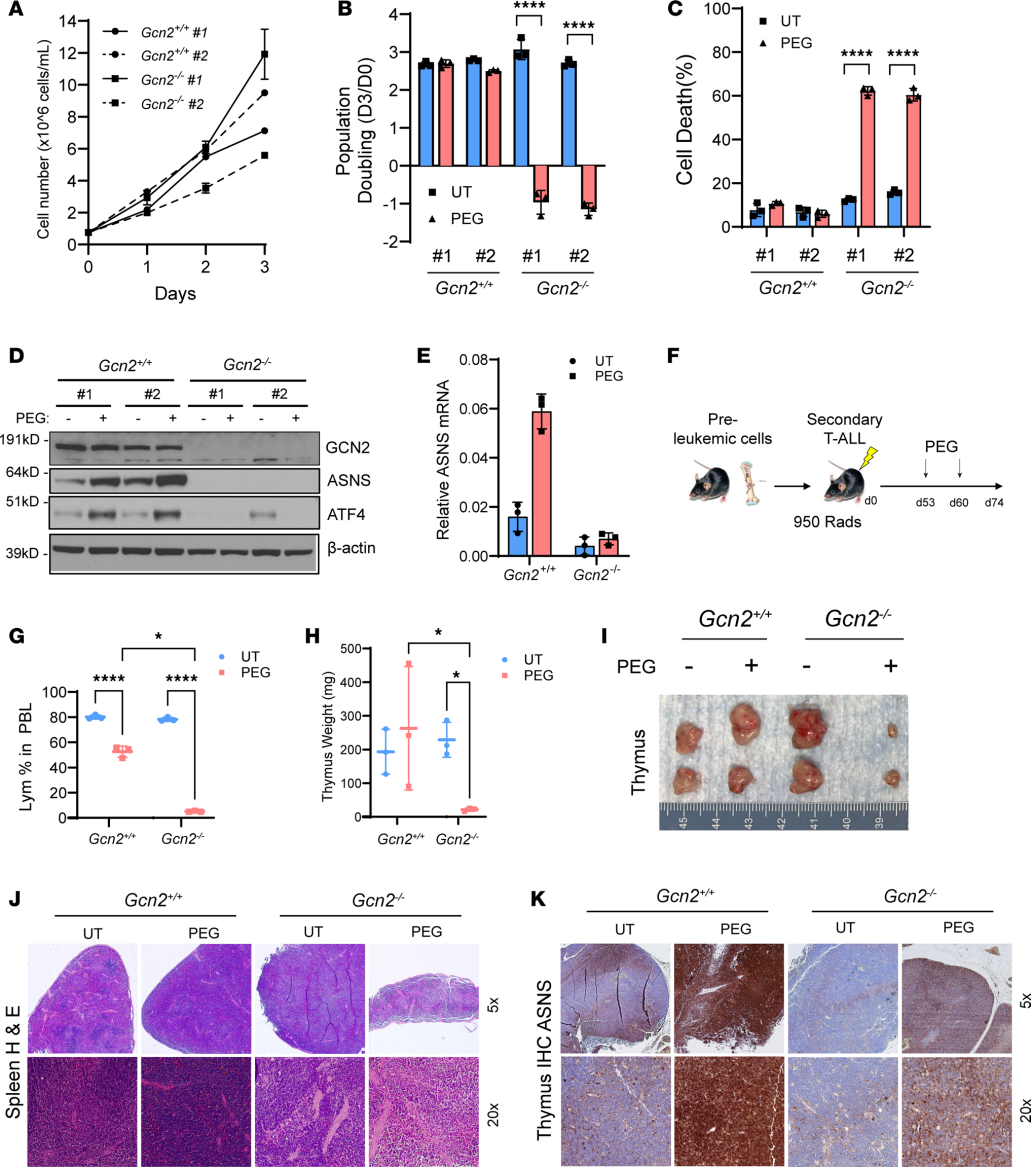
GCN2 deficiency sensitizes ALL cells to asparagine depletion. (**A**) *Gcn2^+/+^* and *Gcn2^–/–^* murine leukemic lines were cultured in RPMI media and cell numbers were recorded over a 3-day period. (**B** and **C**) *Gcn2^+/+^* and *Gcn2^–/–^* T-ALL cells from panel **A** were treated with pegylated L-asparaginase (PEG) (0.01 IU/mL) or untreated (UT) over a 3-day period. Population doublings and cell viability were measured on day 3. Statistical significance was determined using 2-way ANOVA. ****P < 0.0001. (**D**) *Gcn2^+/+^* and *Gcn2^–/–^* T-ALL mouse cell lines were treated with PEG for 16 hours to collect cell lysates. Western blot was used to detect GCN2, ASNS, and ATF4 proteins. (**E**) RNA was isolated from representative *Gcn2^+/+^* and *Gcn2^–/–^* mouse T-ALL cells treated with or without PEG for 16 hours. qPCR was used to measure *Asns* mRNA levels. (**F**) Schematic illustration of secondary T-ALL model. Seven-week-old preleukemic bone marrow cells from *Kras^G12D^*;*Gcn2^+/+^* and *Kras^G12D^;Gcn2^–/–^* mice were transplanted into lethally irradiated WT recipients. Mice received 2 doses of PEG at 53 and 60 days after transplantation (arrows). Mice were euthanized on day 74 after transplantation. (**G** and **H**) Lymphocyte percentages in peripheral blood and thymus weights from **F** were recorded at the experimental endpoint (*n* = 3 per group). Statistical significance was determined using 2-way ANOVA (**G**) and multiple unpaired *t* test (**H**). *P < 0.05; ****P < 0.0001. (**I**) Images of representative thymus from mice in **F**. (**J**) Representative image of H&E-stained mouse spleen from **F**. (**K**) Representative IHC staining for ASNS in thymus tissues from mice in **I**.

To assess the impact of GCN2 loss on asparagine depletion, we established primary T-ALL cell lines from *Gcn2^+/+^* and *Gcn2^–/–^* leukemias. All lines proliferate normally in asparagine-containing RPMI medium (Figure 2A). However, upon treatment with pegylated L-asparaginase (PEG), *Gcn2^–/–^* cells failed to proliferate and underwent cell death, while *Gcn2^+/+^* cells remained viable and proliferating (Figure 2, B and C). Western blotting analysis confirmed that *Gcn2^–/–^* cells failed to induce ATF4 and ASNS protein following PEG treatment (Figure 2D), and the failure of ASNS protein induction was due to mRNA expression (Figure 2E).

To assess the impact of GCN2 deficiency in response to L-asparaginase treatment in vivo, we employed our secondary T-ALL model with *Gcn2^+/+^* and *Gcn2^–/–^* preleukemic bone marrow transplantation. Mice received 2 doses of PEG 1 week apart, and analysis was performed 2 weeks after the second dose (Figure 2F). PEG treatment resulted in a greater reduction in peripheral lymphocyte percentage and thymus size in *Gcn2^–/–^* than *Gcn2^+/+^* mice (Figure 2, G–I). Hematoxylin and eosin (H&E) staining revealed decreased leukemic infiltration in *Gcn2^–/–^* spleen (Figure 2J). IHC staining of ASNS showed strong induction in *Gcn2^+/+^* but only modest induction in *Gcn2^–/–^* thymic tissues (Figure 2K). These findings demonstrate that GCN2 is required for ASNS induction during asparagine depletion, and its loss sensitizes leukemia cells to L-asparaginase both in vitro and in vivo.

### A small-molecule inhibitor of GCN2 sensitizes ALL cells to asparagine depletion therapy.

**Figure 3:**
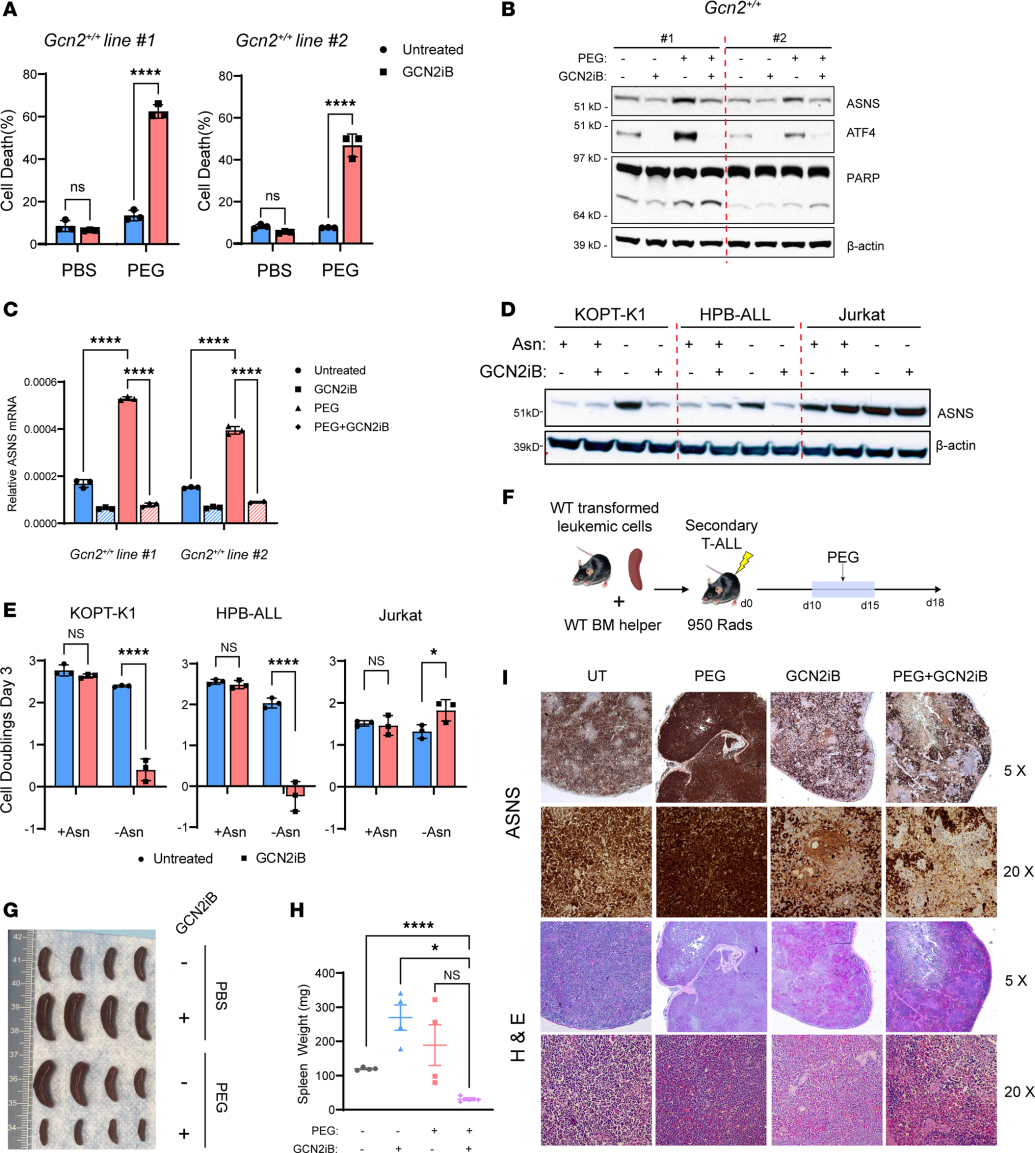
A small-molecule inhibitor of GCN2 sensitizes ALL cells to asparagine depletion. (**A**) *Gcn2^+/+^* lines 1 and 2, from Figure 2A, were treated with or without PEG (0.01 IU/mL) in the presence or absence of GCN2iB (2.5 μM). Cell viability was recorded on day 3. Statistical significance was determined using 2-way ANOVA. ****P < 0.0001. (**B** and **C**) *Gcn2^+/+^* lines 1 and 2 were treated as described in **A** for 16 hours. ASNS, ATF4, and PARP protein levels were assessed by Western blot (**B**), and ASNS mRNA was measured by qPCR (**C**). (**D**) Human T-ALL cell lines, KOPT-K1, HPB-ALL, and Jurkat were grown with or without exogenous asparagine in the presence or absence of GCN2iB (2.5 μM) for 24 hours. ASNS protein level was detected by Western blot. (**E**) KOPT-K1, HPB-ALL, and Jurkat cells were treated with GCN2iB (2.5 μM) for 3 days in the presence or absence of exogenous asparagine. Population doublings on day 3 were recorded. Statistical significance was determined using 2-way ANOVA. *P < 0.05; ****P < 0.0001. (**F**) Schematic illustration of a secondary *Kras^G12D^* T-ALL model. *Kras^G12D^;Lck-Cre;Gcn2^+/+^* primary leukemic spleen cells were transplanted into lethally irradiated mice. GCN2iB was administered daily via oral gavage at 30 mg/kg for 5 days starting at 10 days after transplantation. PEG was administered on day 13 after transplantation and mice were euthanized on day 18. (**G**) Images of representative spleen from mice in **F**. (**H**) Spleen weight of the mice treated in **G** were recorded, and the Brown-Forsythe ANOVA test was used to determine the *P* values. *P < 0.05; ****P < 0.0001. (**I**) IHC staining of ASNS (top) and H&E staining (bottom) of spleen from mice treated in **G**. Representative microscopy images were obtained at ×5 or ×20 magnification as indicated.

We next tested whether pharmacological inhibition of GCN2 phenocopied genetic deletion of GCN2. Treatment of *Gcn2^+/+^* T-ALL lines with GCN2iB (6), a small-molecule inhibitor of GCN2, induced cell death only in combination with PEG (Figure 3A). Consistent with our prediction, GCN2iB blocked ATF4 and ASNS induction after PEG treatment, which correlated with increased PARP cleavage (Figure 3B). The suppression of ASNS protein induction by GCN2iB is attributable to inhibition of its mRNA induction (Figure 3C). In human T-ALL lines, GCN2iB similarly suppressed ASNS induction following asparagine depletion in KOPT-K1 and HPB-ALL cells, but not in Jurkat cells, which expressed high basal ASNS (Figure 3D). Correspondingly, GCN2iB suppressed proliferation in KOPT-K1 and HPB-ALL cells, but not Jurkat cells, only when asparagine was removed from the media (Figure 3E). GCN2iB inhibited eIF2α phosphorylation and ATF4 induction in KOPT-K1 and HPB-ALL cells within 4 hours of asparagine depletion (Supplemental Figure 2A), preceding ASNS induction. At 16 hours after asparagine depletion, GCN2iB suppressed both ASNS mRNA and protein expression (Supplemental Figure 2B). These results suggest that GCN2iB can effectively suppress the induction of ASNS in ALL cells expressing low levels of ASNS. Furthermore, eIF2α phosphorylation and ATF4 induction are early events of GCN2 activation, and the induction of ASNS requires a longer time, likely due to mRNA transcription and protein synthesis.

To test efficacy in vivo, primary leukemic cells from *Gcn2^+/+^* mice were transplanted into recipients to generate a more aggressive secondary T-ALL model. The advantage of this model is its short latency of approximately 2 weeks, making it ideal for therapeutic testing of GCN2iB and/or PEG (Figure 3F). While each monotherapy had variable effects on leukemia progression in spleen (no statistical significance of spleen weight between untreated and each monotherapy), combination treatment consistently normalized spleen size (Figure 3, G and H). IHC staining of ASNS showed elevated expression of ASNS in PEG-treated spleen, which was blocked by GCN2iB, correlating with a reduction in leukemia burden (Figure 3I). Together, these results demonstrate that GCN2 inhibition enhances the efficacy of L-asparaginase therapy by suppressing the upregulation of ASNS in T-ALL following treatment.

### GCN2-indepenent mechanisms contribute L-asparaginase resistance via ASNS induction.

**Figure 4:**
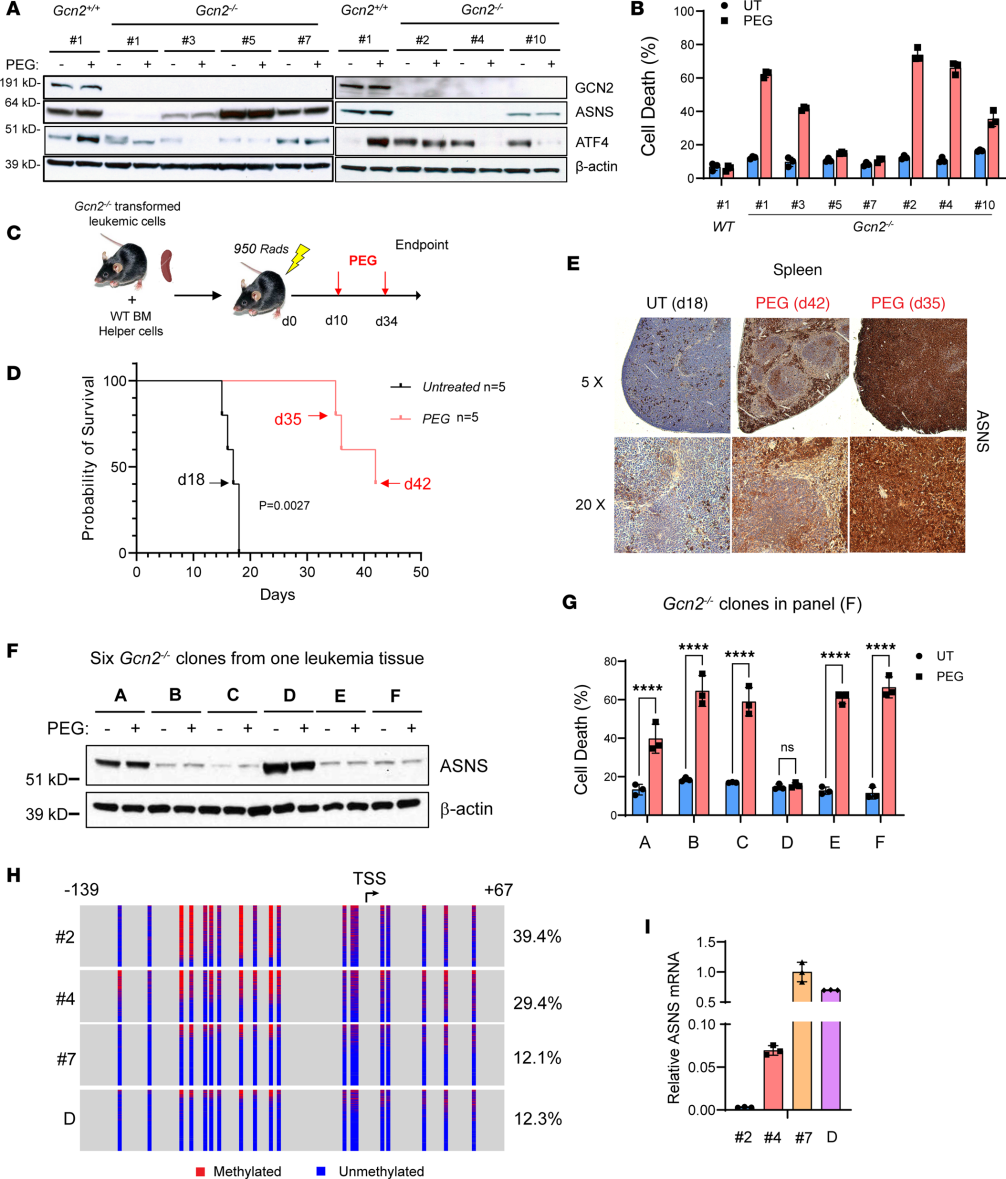
GCN2 inactivation drives GCN2-independent expression of ASNS to confer L-asparaginase resistance. (**A**) *Gcn2^–/–^* T-ALL lines 1, 3, 5, 7, 2, 4, and 10 were treated with PEG for 16 hours. GCN2, ASNS, and ATF4 proteins were assessed by Western blot. (**B**) T-ALL lines in **A** were subjected to PEG treatment or untreated (UT) for 3 days, and cell death percentage was recorded by trypan blue staining. (**C** and **D**) Schematic illustration of a secondary T-ALL model established by using *Gcn2^–/–^* primary *Kras^G12D^;Lck-Cre* leukemia cells. PEG was given in 2 doses on days 10 and 34 after transplantation. Kaplan-Meier curve comparing survival between the untreated and PEG-treated mice. Statistical comparison between the 2 groups was done using the log-rank (Mantel-Cox) test. (**E**) IHC staining of ASNS from the spleen of mice euthanized on day 18 (untreated), day 35 (PEG), and day 42 (PEG). Representative microscopy images were obtained at ×5 or ×20 magnification, as indicated. (**F** and **G**) Six single-cell-derived clones from the same untreated spleen tissue in **E** were subjected to PEG treatment for 16 hours, and protein lysates were subjected to Western blotting analysis for ASNS (**F**). Cell death percentage following PEG treatment for 3 days were recorded (**G**). ****P < 0.0001 by 2-way ANOVA. (**H**) DNA methylation status in the CpG island of the *Asns* promoter was determined by bisulfite sequencing in *Gcn2^–/–^* lines 2, 4, 7, and clone D. Results are shown as heatmaps. The percentages of methylated CpG are indicated on the right of each panel. TSS, transcription start site. (**I**) *Asns* mRNA levels from the cells in **H** were assessed by qPCR.

In clinical applications of L-asparaginase, there are reports of ALL resistance that feature enhanced ASNS expression (18), suggesting a selection for variants that boost synthesis of asparagine likely by GCN2 activation and induction of ATF4 that overcome the L-asparaginase–based therapy (19). However, we observed that 2 out of 7 *Gcn2^–/–^* T-ALL lines expressed high levels of ASNS (Figure 4A, lines 5 and 7). As a result, lines 5 and 7 were fully resistant to PEG treatment in vitro (Figure 4B). The remaining cell lines are sensitive or partially sensitive to PEG, correlating with ASNS protein expression (Figure 4, A and B). Notably, shRNA-mediated knockdown of ATF4 or ZBTB1 — 2 transcription factors known to regulate ASNS expression (20, 21) — in cell line 7 did not alter ASNS expression and had minimal effects on cell proliferation and survival following PEG treatment (Supplemental Figure 3, A–D), indicating a GCN2/ATF4- and ZBTB1-independent mechanism regulating ASNS expression.

To determine whether GCN2 inactivation in vivo selects for GCN2-independent mechanisms driving ASNS expression and L-asparaginase resistance, we treated leukemia-bearing mice transplanted with primary *Gcn2^–/–^* leukemic cells with PEG (Figure 4C). PEG treatment significantly delayed leukemia-associated lethality (Figure 4D). Analyzing spleen tissues from untreated and PEG-treated mice showed that ASNS expression was markedly increased in PEG-treated animals at the endpoint of morbidity (day 35) (Figure 4E). In contrast, animals euthanized on day 42 remained healthy and exhibited intermediate ASNS induction (Figure 4E). Consistent results were observed in an independent experiment using primary leukemia cells derived from a separate *Gcn2^–/–^* donor (Supplemental Figure 3, E and F). In this cohort, all 5 PEG-treated animals reached morbidity (Supplemental Figure 3E), and 2 representative animals (days 25 and 43) displayed elevated expression of ASNS (Supplemental Figure 3F).

To determine whether heterogeneity in ASNS expression contributes to its induction in the in vivo experiment, we established multiple single-cell-derived T-ALL clones from spleen cells harvested from an untreated mouse on day 18 (Figure 4, D and E). Six clones derived from the same spleen exhibited variable ASNS expression (Figure 4F). Furthermore, their sensitivity to PEG treatment was inversely correlated with ASNS protein expression (Figure 4G).

We previously showed that promoter demethylation of human *ASNS* is required for ATF4-dependent ASNS expression in human T-ALL lines (8). To assess the role of promoter demethylation of the *Asns* gene in mouse leukemia cells, we performed bisulfite sequencing in 4 *Gcn2^–/–^* T-ALL lines expressing variable levels of ASNS. We found that the degree of promoter methylation was inversely correlated with ASNS expression at both protein and mRNA levels (Figure 4, A, F, H, and I), indicating a role for promoter demethylation in driving ASNS expression in *Gcn2^–/–^* leukemia cells. These results are consistent with a model where expression of ASNS is a key driver of L-asparaginase resistance. Furthermore, the ASNS induction can occur even when induction of GCN2 and the ISR are disabled.

### ASX-173 overcomes resistance caused by GCN2-independent ASNS induction.

**Figure 5:**
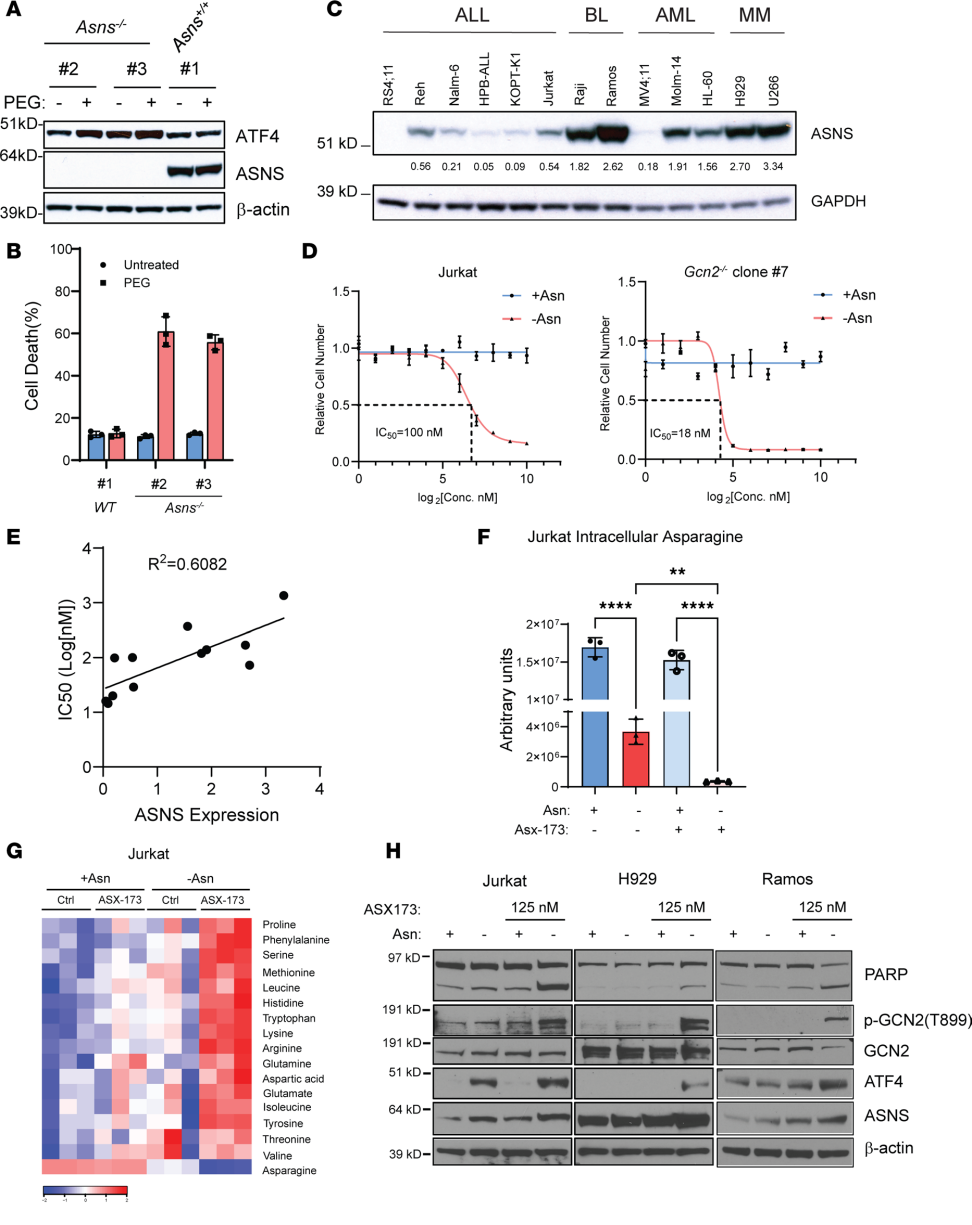
ASX-173 overcomes L-asparaginase resistance in ALL cells due to induced ASNS expression independent of GCN2. (**A**) *Kras^G12D^;Lck-Cre* mice were crossed with *Asns^loxP/loxP^* mice (32). Mouse T-ALL lines from 2 *Asns^–/–^* mice (lines 2 and 3) were treated with PEG for 16 hours. A WT mouse T-ALL line was used as a control. ATF4 and ASNS were assessed by Western blot. (**B**) Mouse T-ALL lines in **A** were treated with PEG for 3 days and cell death was recorded by trypan blue staining. (**C**) Basal ASNS expression was measured by Western blot analysis in a panel of human hematological cancer cell lines. ALL (RS4;11, Reh, Nalm-6, HPB-ALL, KOPT-K1, Jurkat), Burkitt lymphoma (BL) (Raji and Ramos), acute myeloid leukemia (AML) (MV4;11, Molm-14, HL-60), and multiple myeloma (MM) (H929, U266). (**D**) Human T-ALL Jurkat cells and mouse T-ALL *Gcn2^–/–^* line 7 from Figure 4A were grown with or without asparagine for 2 days, in the presence of a range of ASX-173 doses (2–1024 nM). IC_50_ values were measured via MTT assay. (**E**) IC_50_ values from **D** and Supplemental Figure 4A were plotted against basal ASNS expression from **C**. (**F**) Jurkat cells were treated with ASX-173 (125 nM) in the presence or absence of exogenous asparagine for 16 hours. Intracellular asparagine levels were measured by liquid chromatograph–mass spectrometry (LC-MS). Statistical significance was determined using 1-way ANOVA. **P < 0.01; ****P < 0.0001. (**G**) Heatmap showing intracellular levels of amino acids in Jurkat cells treated in **F** (*n* = 3). (**H**) Jurkat, H929, and Ramos cells were cultured with or without asparagine in the presence or absence of ASX-173 (125 nM) for 24 hours. PARP, p-GCN2 (T899), ATF4, and ASNS proteins were assessed via Western blot analysis.

To further test whether ASNS is the key driver of resistance to L-asparaginase treatment, we generated *Kras^G12D^;Lck-Cre;Asns^fl/fl^* mice, allowing for concurrent *Kras^G12D^* activation and *Asns* deletion in T cells. Leukemia lines derived from these mice lack ASNS and were highly sensitive to PEG treatment (Figure 5, A and B), confirming ASNS as a key resistance determinant. These results suggest that targeting ASNS will directly overcome resistance to L-asparaginase in ASNS^hi^ cancer cells.

To address the variations in ASNS expression among human hematopoietic cancers, we measured the expression of ASNS protein in lines derived from ALL, Burkitt lymphoma, acute myeloid leukemia, and multiple myeloma. The levels of ASNS as judged by Western blot varied, with ALL lines showing the lowest levels (Figure 5C). These results are consistent with the literature (22, 23), and align with the clinical success of L-asparaginase in the treatment of ALL patients (24, 25).

We next pursued the idea of targeted inhibition of ASNS to expand the utility of L-asparaginase to help alleviate resistance in ALL and potentially expand L-asparaginase among hematopoietic cancers. ASX-173 is a recently reported cell permeable ASNS inhibitor (12, 26). We found that all tested hematopoietic cancer lines responded to ASX-173 when asparagine is depleted from the cell culture media (Figure 5D and Supplemental Figure 4A), with most of these lines showing IC_50_ values of ASX-173 below 200 nM. None of the tested cell lines responded to ASX-173 even up to 1 μM when asparagine was present in the media (Figure 5D and Supplemental Figure 4A), indicating high specificity of this drug for ASNS. Furthermore, the IC_50_ values of ASX-173 correlated well with ASNS protein expression in these lines (Figure 5E).

Metabolomic profiling in Jurkat cells confirmed a 70% reduction in intracellular asparagine when exogenous asparagine was removed (Figure 5F); however, this reduction was further exaggerated (>95%) when ASX-173 was given simultaneously (Figure 5F). Notably, there was no reduction in other amino acids, but changes in nucleotides and their precursors when combining ASX-173 with asparagine depletion (Figure 5G and Supplemental Figure 4, B and C). In Jurkat, H929, and Ramos cells, ASX-173 induced PARP cleavage under asparagine-depleted conditions, accompanied with increased GCN2 phosphorylation and ATF4 accumulation (Figure 5H). These results suggest that leukemia cells primarily acquire asparagine through uptake, rendering ASX-173 ineffective when extracellular asparagine is present (Figure 5D); however, upon depletion of exogenous asparagine, cells rely exclusively on ASNS for intracellular asparagine synthesis (Figure 5F). Consequently, ASX-173 triggers ISR and cell death only in the absence of exogenous asparagine (Figure 5H).

### ASX-173 suppresses leukemia progression in ASNS-high ALL in vivo.

**Figure 6:**
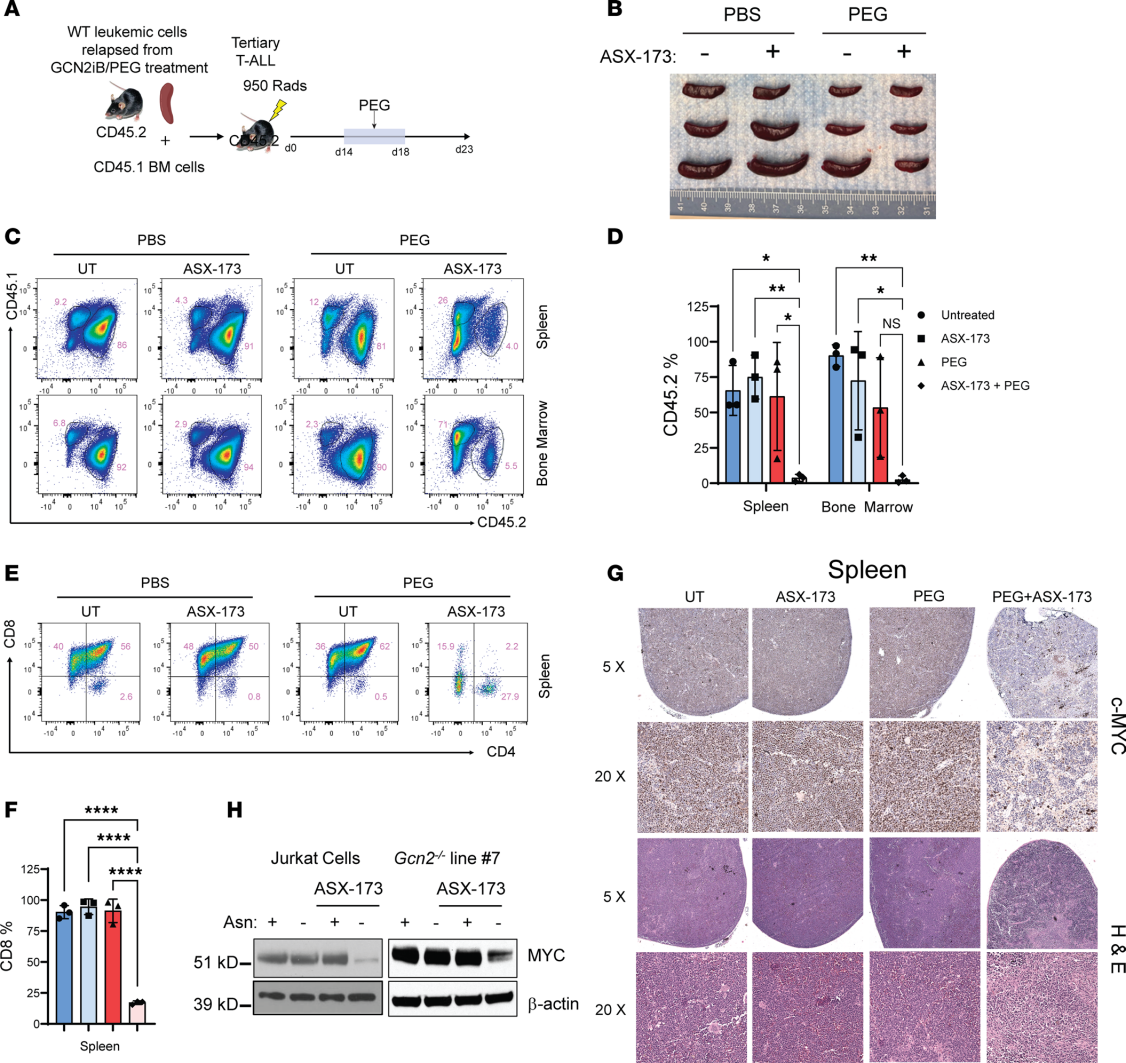
ASX-173 reduces leukemic burden in an ASNS-high ALL model when coupled with L-asparaginase. (**A**) Schematic illustration of tertiary transplant of leukemia cells from *Gcn2^+/+^* mice relapsed from PEG and GCN2iB combo treatment. Lethally irradiated mice were transplanted with 1.2 × 10^6^ CD45.2^+^ leukemia cells and 0.5 × 10^6^ CD45.1^+^ helper bone marrow cells. ASX-173 was administered at 25 mg/kg for 5 days via oral gavage, starting on day 14 after transplantation (blue box). PEG was administered at 2.0 IU/g body weight on day 17 after transplantation. Mice were euthanized on day 23. (**B**) Images of representative spleen from mice euthanized on day 23 in **A**. (**C** and **D**) CD45.1 and CD45.2 profile of spleen and bone marrow cells measured by flow cytometry and the summary of CD45.2^+^ cell percentage (*n* = 3). (**E** and **F**) CD4^+^/CD8^+^ cell profile of CD45.2^+^CD3^+^ T cells from spleens in **B** and the summary of CD8^+^ cell percentage (*n* = 3). Statistical significance was determined using 2-way ANOVA. *P < 0.05; **P < 0.01; ****P < 0.0001. (**G**) Spleen tissues from **B** were subjected to IHC staining for c-MYC and H&E staining. Representative microscopy images were obtained at ×5 or ×20 magnification, as indicated. (**H**) Human Jurkat cells and mouse *Gcn2^–/–^* line 7 were subjected to asparagine depletion for 12 hours with or without ASX-173 (125 nM). c-MYC expression was assessed by Western blotting.

ASX-173 demonstrated promising therapeutic potential in our in vitro experiment when combined with asparagine depletion. To test whether ASX-173 can overcome resistance of L-asparaginase in ASNS-high leukemias in vivo, we established another transplantable secondary T-ALL model. In the experiment in Figure 3, F–I, we retained animals in the GCN2iB plus PEG group for long-term monitoring. Many of the treated animals relapsed (spleen weight > 350 mg) 2 weeks after treatment (Supplemental Figure 5A). Leukemic tissues from relapsed animals expressed high levels of ASNS (Supplemental Figure 5B), which provided a model for therapy-resistant disease. As highlighted in the illustration of our experimental design (Figure 6A), we used CD45.1^+^ bone marrow cells as the helper cells to differentiate normal hematopoietic cells (CD45.1^+^) and leukemia cells (CD45.2^+^) (Figure 6A). ASX-173 was given by oral gavage at 1 dose per day for 5 days, and PEG was administrated after the third dose of ASX-173 (Figure 6A). The combination treatment of ASX-173 with PEG markedly reduced spleen size (Figure 6B) and CD45.2^+^ leukemia burden in spleen and bone marrow (Figure 6, C and D). Of interest, combining ASX-173 with PEG restored the spleen CD4^+^/CD8^+^ cell profile to near normal in recipient mice, which was not seen by monotherapy (Figure 6, E and F). In addition, combining ASX-173 with PEG markedly reduced MYC staining in the spleen tissues (Figure 6G), correlating with a reduction in leukemia burden. Consistently, ASX-173 suppressed MYC protein expression in Jurkat and *Gcn2^–/–^* line 7 cells in vitro only when exogenous asparagine was depleted (Figure 6H). PEG treatment resulted in a modest decline in body weight, which was not exaggerated by ASX-173 at the dose used (Supplemental Figure 5C). Histology and IHC studies revealed that combining ASX-173 and PEG markedly reduced leukemia infiltration into the liver and partially restored a normal liver architecture (Supplemental Figure 5D). Together, these findings suggest that ASX-173 enhances the efficacy of PEG-mediated asparagine depletion in suppressing leukemia progression in vivo, with no evidence of prohibitive toxicity.

## Discussion

GCN2 is a central component of the ISR, functioning as a sensor of amino acid deficiency (7). Its role in cancer has only recently been appreciated. In solid tumor models, the GCN2/ATF4 pathway has been shown to be critical for tumor adaptation to amino acid–limited microenvironments (27). Similarly, a recent study reported that GCN2 is required for prostate cancer proliferation even under amino acid–replete conditions (9), suggesting that these tumors may exist in nutrient-poor microenvironments and rely on GCN2 to regulate amino acid acquisition. However, these studies utilized xenograft models and fully transformed tumor cells, which may not accurately reflect the metabolic state during tumor initiation. In a mouse genetic model of soft tissue sarcoma, *Gcn2* deletion did not impair tumor initiation or progression; instead, compensatory PERK activation maintained ISR signaling (28), indicating its role in de novo tumorigenesis may be context dependent.

Using a mouse genetic model of de novo leukemogenesis, we demonstrate that GCN2 is dispensable for *Kras^G12D^*-driven T-ALL initiation and progression (Figure 1). In contrast to the sarcoma study, we did not observe compensatory PERK activation in the absence of GCN2 (Supplemental Figure 1). These results suggest that leukemia cells may reside in a microenvironment with sufficient amino acid availability during initiation and early progression. The upregulation of ISR signaling observed in leukemia tissues compared with normal tissues (Figure 1, D and E) may instead reflect increased local amino acid consumption at later stages of the disease. Collectively, these observations suggest that the role of GCN2 in tumor initiation is context dependent, likely influenced by cell of origin, microenvironment, and oncogenic signaling. Future studies should evaluate the role of GCN2 in additional genetic models of hematologic malignancies, such as acute myeloid leukemia and B cell lymphoma.

Despite its dispensability in leukemia initiation and progression, our results clearly showed that GCN2 becomes essential under asparagine-depleted conditions, mediating ATF4/ASNS activation and conferring resistance to L-asparaginase (Figures 2 and 3). In contrast to prostate cancers (9), GCN2-deficient T-ALL cells grow and proliferate normally in amino acid–replete media, suggesting their full capacity of uptake. Using both genetic and pharmacological approaches, we showed that inhibition of GCN2 significantly reduced the induction of ASNS by asparagine depletion and sensitized primary T-ALL to L-asparaginase treatment in vivo. These results are consistent with a report where GCN2iB sensitizes ASNS^lo^ cancer cells to L-asparaginase therapy (6). However, GCN2iB has no effect in ASNS^hi^ cancer cells because they do not rely on GCN2 to drive the expression of ASNS.

To our surprise, we identified a subset of *Gcn2^–/–^* leukemias expressing high levels of ASNS regardless of L-asparaginase treatment (Figure 4A). Notably, ASNS protein expression remained unchanged following shRNA-mediated knockdown of ATF4, a key transcription factor upstream of the *Asns* gene (8, 20), suggesting a GCN2/ATF4-independent regulatory mechanism. Importantly, single-cell-derived clones from the same *Gcn2^–/–^* leukemic tissue exhibited variable ASNS expression (Figure 4, F and G), indicating that cellular heterogeneity may contribute to the selection of GCN2/ATF4-independent mechanisms driving ASNS expression and L-asparaginase resistance in vivo (Figure 4, D and E). Recent work has implicated the transcription factor ZBTB1 in ASNS transcription via promoter recruitment in human T-ALL cells (21). However, shRNA-mediated knockdown of ZBTB1 in ASNS^hi^ *Gcn2^–/–^* mouse T-ALL cells did not affect ASNS expression (Supplemental Figure 3C). Whether additional transcription factors are involved in this context warrants further investigation.

In contrast, we found that the degree of DNA demethylation within the CpG island of the *Asns* promoter correlates with ASNS expression (Figure 4, H and I), indicating a critical role for promoter demethylation in GCN2/ATF4-independent ASNS regulation. These findings raise the concern that combination of GCN2iB with L-asparaginase may enable selection for GCN2-independent ASNS induction, ultimately leading to therapeutic resistance (Supplemental Figure 5, A and B). Consistent with this, human T-ALL cells (Jurkat) also express high levels of ASNS under amino acid–replete conditions, rendering them insensitive to the combination of GCN2iB and asparagine depletion (Figure 3, D and E). Collectively, these results support the development of therapeutics that directly target ASNS.

The role of ASNS in solid tumors has been recently explored extensively (5). All these studies indicate that it is a promising therapeutic target under asparagine-restricted conditions, except for the lack of effective inhibitors. We found that ASX-173 is a potent and selective ASNS inhibitor with minimal off-target toxicity in vitro. Combined ASX-173 and L-asparaginase treatment suppressed both ALL and other hematologic malignancies with high ASNS expression (Figure 5D and Supplemental Figure 4A), potentially broadening the therapeutic utility of L-asparaginase. Metabolomic profiling revealed that ASX-173 specifically depleted intracellular asparagine when exogenous asparagine was withdrawn (Figure 5, F and G). Of interest, levels of all the other amino acids were increased by the same treatment (Figure 5G). Whether this is due to a suppression of global protein synthesis and thereby cells accumulate amino acids (29), or other mechanisms, remains to be determined. Furthermore, pool of nucleotides and their precursors were altered by ASX-173 in asparagine-depleted conditions (Supplemental Figure 4C). This result is unexpected, as asparagine cannot be used as a biosynthetic precursor in mammalian cells due to their lack of endogenous asparaginase activities (29). Thus, we speculate that the alteration of nucleotides and their precursors may be a secondary effect of growth inhibition or indicate an allosteric regulation of nucleotide biosynthesis by asparagine, which warrants further investigation.

To test the efficacy of ASX-173 in vivo, we applied a tertiary transplantable T-ALL by using the leukemia cells relapsed from a combined treatment with GCN2iB and L-asparaginase (Supplemental Figure 5, A and B). These leukemia cells express high levels of ASNS and are suitable tools to test the efficacy of ASX-173 in vivo. We found that combining ASX-173 with L-asparaginase in vivo markedly suppresses leukemia progression (Figure 6), which cannot be achieved by monotherapy. The fact that ASX-173 synergizes with L-asparaginase to reduce MYC expression in leukemia cells may indicate the importance of asparagine in regulating MYC mRNA translation (30), which warrants further investigation. Importantly, ASX-173 plus L-asparaginase therapy did not significantly impact body weight or normal T cell homeostasis, and even partially restored normal liver morphology (Figure 6E and Supplemental Figure 5, C and D), indicating the treatment is well tolerated in mice. In liver, even PEG monotherapy substantially reduced leukemia infiltration, with most leukemia cells found near the blood vessels (Supplemental Figure 5D), suggesting a potential role of circulating asparagine in leukemia infiltration to other tissues, a phenomenon sharing similarity to metastasis in solid tumors (31). Given the adverse effects associated with L-asparaginase, including pancreatitis and allergic reactions (4), future studies exploring dietary asparagine restriction in combination with ASX-173 may provide safer therapeutic options. In summary, our work establishes that GCN2 is dispensable for T-ALL initiation but critical for resistance to L-asparaginase via ASNS induction. Direct ASNS inhibition using ASX-173 represents a promising strategy to overcome resistance driven by GCN2-independent mechanism and potentially expand asparagine-targeted therapies beyond ALL.

## Methods

### Sex as a biological variable.

Sex is a biological variable in the context of leukemia. Both males and females were used for animal experiments in this study.

### Cell culture and primary cell line establishment.

Human T-ALL cell lines were cultured in our lymphocyte culture medium (LCM) at 37°C in 5% CO_2_. LCM was prepared as previously described (8) using high-glucose DMEM (11965092, Thermo Fisher Scientific) as a base supplemented with 0.1 mM final concentration of asparagine. Primary leukemic lines derived from T-ALL mice were cultured in RPMI media (11875093, Thermo Fisher Scientific). Both media contained 10% FBS, 100 U/mL penicillin/streptomycin, and 55 μM β-mercaptoethanol. A list of human cell lines, disease type, and origin is supplied in Supplemental Table 1. Primary mouse T-ALL lines were established by plating spleen leukemia cells on methylcellulose (03231, STEMCELL Technologies). Cells from all colonies on the plate were pulled and transferred into RPMI medium to expand until they became stable cell lines. Single-cell-derived clones were established by plating cells at a low density (~50,000 per 3-cm dish). Colonies were picked 10–14 days after plating and expanded into RPMI medium.

### Cell growth and viability assay.

Cell growth and viability experiments were done using a Vi-CellXR cell analyzer (Beckman Coulter), which estimates viable cells based on trypan blue exclusion. Assays were performed in triplicate. Population doubling was calculated by normalizing cell numbers to day 0, followed by a logarithmic transformation with base 2.

### Asparagine depletion experiments.

To deprive human T-ALL cell lines of asparagine, cells were centrifuged at 400 g for 5 minutes at room temperature. The supernatant was removed, and cells were resuspended in asparagine-deficient LCM. Asparagine-deficient LCM was prepared as previously described, except it was supplemented with 10% dialyzed FBS and no asparagine supplementation. For cells cultured in RPMI, asparagine depletion was achieved by adding the clinically used pegylated asparaginase (Asparlas) to a final concentration of 0.01 IU/mL.

### Flow cytometry.

For immunophenotyping, cells were collected from peripheral blood, spleen, and bone marrow of T-ALL mice. Bone marrow was flushed with 1 mL of PBS using a 25-gauge needle into a microcentrifuge rube and kept on ice. Spleens were crushed and filtered with PBS using a 40 μm nylon cell strainer (210804-301, MIDSCI). Cells were collected by centrifugation at room temperature. Red blood cells were removed by using 1× RBC lysis buffer (sc-296258, Santa Cruz Biotechnology), as recommended by the manufacturer. The harvested single cells were resuspended with blocking buffer containing 10% goat serum (16210064, Gibco) and purified anti-mouse CD16/CD32 antibody (101302, BioLegend) in PBS and kept on ice for 10 minutes. After centrifugation, the supernatant was discarded, and cells were stained with fluorescence-conjugated cell-surface antibodies and run on an Attune NxT Flow cytometer. A list of the antibodies used can be found in Supplemental Table 2. Annexin V/PI staining was used to assess apoptosis. Cells were pelleted by centrifugation followed by a PBS wash to remove remaining media. Cells were resuspended in 100 μL of 1× Annexin V binding buffer (1 mM HEPES pH 7.4, 15 mM NaCl, and 0.25 mM CaCl_2_), supplemented with 5 μL FITC-Annexin V (640905, BioLegend) and 2 μL of propidium iodide solution (P3566, Thermos Fisher Scientific). Cells were incubated for 15 minutes in the dark, followed by washing the excess dye using 1× binding buffer. Cells were run on the Attune NxT flow cytometer using the recommended channels. Data were analyzed using FlowJo software (BD Biosciences).

### Western blotting.

Cells were centrifuged and pellets were washed with PBS. Cells were lysed using RIPA lysis buffer (20-188, EMD Millipore) supplemented with Halt Protease inhibitor (87785, Thermo Fisher Scientific), and Halt Phosphatase inhibitor (78420, Thermo Fisher Scientific). Protein concentrations were measured using Bradford’s reagent (5000006, Bio-Rad) as described in the manufacturer’s protocol. Equal amounts of protein were loaded and separated via electrophoresis on a 4%–12% Bis-Tris gel (NP0322BOX, Invitrogen) using MOPS SDS running buffer (NP0001, Invitrogen). Protein was transferred using 1× transfer buffer (NP00061, Invitrogen) onto a 0.45 μm nitrocellulose membrane (1620115, Bio-Rad). Membrane was blocked using 5% milk (232100, Difco) prepared in 1× Tris-buffered saline with Tween 20 (TBST) (sc-362311, Santa Cruz Biotechnology). The membrane was incubated with primary antibody overnight at 4°C and washed 3 times for 10 minutes each at room temperature with 1× TBST. Afterwards, the membrane was incubated at room temperature for 1 hour with horseradish peroxidase–conjugated secondary antibody. Then membrane was washed as described previously. Signal detection was done by SuperSignal West Pico PLUS Chemiluminescent Substrate (34578, Thermo Fisher Scientific). The membrane was stripped for detection of other proteins using Restore Western Blot stripping buffer (21059, Thermo Fisher Scientific) as per the manufacturer’s recommendation. The membrane was then reprobed with other primary antibodies. A list of antibodies used in this study is provided in Supplemental Table 2.

### mRNA quantification by PCR.

Approximately 5 × 10^6^ cells were centrifuged and resuspended in Trizol reagent (15596026, Life Technologies) to collect total RNA following the manufacturer’s protocol. RNA (500–1000 ng) was used for cDNA synthesis using a ProtoScript II first strand cDNA synthesis kit (E6560, NEB). Quantitative PCR (qPCR) was done using EvaGreen qPCR master mix (BEQPCR, MIDSCI) on a QuantStudio 3 (Applied Biosystems). A list of primers used in the qPCR analyses in this study is included in Supplemental Table 3.

### MTT assay.

Cells were plated at an initial density of 0.4 × 10^6^ cells/mL as indicated, with or without asparagine with ASX-173 concentrations from 0–1024 nM in technical triplicate. Plates were incubated for 2 days at 37°C in 5% CO_2_. One hundred microliters of cells was used for the MTT assay using a Cell Proliferation Kit I (MTT) (11465007001, Roche) following the manufacturer’s protocol. Absorbance was read on SpectraMax iD3 plate reader (Molecular Devices). Absorbance values were normalized to the respective untreated conditions. IC_50_ graphs were generated in GraphPad Prism by fitting the data to a sigmoidal IC_50_ analysis.

### Animal experiments.

For our primary T-ALL model, we used a *Kras^G12D^* mutation that was activated by a loxP-flanked stop codon (LSL) system driven by a *Lck-Cre* promoter during T cell development. To assess the role of GCN2 in T-ALL development, *Kras^G12D^;Lck-Cre* mice were crossed with *Gcn2^–/–^* mice. To establish a secondary T-ALL model, 7-week preleukemic bone marrows were collected. Preleukemic bone marrow cells (1 × 10^6^) were transplanted via intravenous injection into C57BL/6 mice that received 9.5 Gy of irradiation. In cases we need to transplant primary leukemia cells, we included 0.5 × 10^6^ helper bone marrow cells derived from BoyJ mice (CD45.1^+^). To generate *Asns^–/–^* T-ALL mice, *Kras^G12D^;Lck-Cre* mice were crossed with a *Asns^loxP/loxP^* strain (a gift from Ruoning Wang, Nationwide Children’s Hospital, Columbus, Ohio, USA) (32). Asparaginase treatment was done with the clinically used Asparlas as indicated in the figures. Mice received 2.0 IU/g body weight via intraperitoneal injection. For pharmacological inhibition of GCN2, GCN2iB (2071802, Sun-shine Chemical) was prepared in 5% DMSO/20% Captisol solution. One hundred fifty microliters of GCN2iB was administered daily via oral gavage to achieve a dosage of 30 mg/kg. For pharmacological inhibition of ASNS, ASX-173 (24015, Sun-shine Chemical) was prepared in 10% DMSO/20% Captisol solution. One hundred microliters of ASX-173 was administered daily via oral gavage to achieve a dosage of 25 mg/kg.

### Immunohistochemistry.

Spleen and thymus were sliced into an appropriate size and fixed in 4% paraformaldehyde (PFA) for 24 hours. The next day, the tissues were washed with PBS and stored in 70% ethanol at 4°C. Tissue sectioning, embedding, and staining were done by the Indiana University (IU) Pathology Lab. IHC staining was performed with DAKO Omnis Automated Stainer and antigens were retrieved using DAKO High pH solution. Primary antibodies were against ASNS (ProteinTech, 14681-1-AP; 1:500), p-GCN2 (T899) (Abcam, ab75836; 1:200), and c-MYC (Ventana, 790-4628; ready to use). Slides were counter stained with H&E. Slides were visualized using a Leica DM4 microscope and images captured using the Leica Application Suite X platform.

### Mass spectrum analysis of metabolites.

Jurkat cells (15 × 10^6^ per replicate) were cultured as indicated for 16 hours. Cells were collected by centrifugation, supernatant was aspirated, and the pellets were washed thoroughly once with ice cold 1× HBSS (14025092, Life Technologies). Cellular metabolites were extracted with 80% methanol on ice. Supernatant was collected and dried with SpeedVac (SPD111V, Thermo Fisher Scientific) connected to Refrigerated Vapor Trap (RVT5105, Thermo Fisher Scientific) at room temperature. Dried samples were resuspended in water and analyzed using a Thermo Q-Exactive mass spectrometer coupled to a Vanquish Horizon UHPLC. Metabolites were separated on a 2.1 × 100 mm, 1.7 μm Acquity UPLC BEH C18 Column (Waters) with a gradient of solvent A (97:3 H_2_O/methanol, 10 mM TBA, 9 mM acetate, pH 8.2) and solvent B (100% methanol). The gradient was as follows: 0 minutes, 5% B; 2.5 minutes, 5% B; 17 minutes, 95% B; 21 minutes, 95% B; 21.5 minutes, 5% B. The flow rate was 0.2 mL/min. Metabolites were identified based on exact *m*/*z* and retention time determined using chemical standards. Data were normalized to internal standard of ^13^C_4_,^15^N_2_-asparagine (1 pmole/sample) and then total cell number of each sample. Heatmaps were generated by using the Galaxy Heatmap 2 webtool (https://usegalaxy.org).

### Bisulfite sequencing analysis.

Genomic DNA was isolated from the cells using a Puregene kit (158063, Qiagen) according to the manufacturer’s instructions. The isolated genomic DNA was then subjected to CT conversion using the EZ DNA methylation kit (D5001, Zymo Research) following the manufacturer’s protocol. Primers flanking the mouse *Asns* promoter were designed using the online MethPrimer webtool (https://methprimer.com/). First-round amplification was performed using the hot-start polymerase protocol (E2001, Zymo Research). The PCR product was purified, followed by a second round of amplification of the purified product using barcoding primers (Supplemental Table 3). The PCR products were gel purified and sent for Amplicon-EZ Sequencing (Azenta).

R1 reads were initially demultiplexed with cutadapt v3.7 (https://cutadapt.readthedocs.io/en/v3.7/) using the barcoded sequences with the default error rate of 10%, allowing no mismatches based on indels and having a minimum overlap of 20 bases between read and the barcoded adapter sequence. Bismark v0.24.0 (https://github.com/FelixKrueger/Bismark/releases) index for the promoter sequence was prepared, and reads were aligned to the prepared reference using end-to-end mode (default for bowtie2-based alignments) allowing for 1 mismatch, with a seed length of 20 (for sensitive matches) and a scoring function L, 0, –0.6 to control read mapping. Methylation events were extracted using the bismark_methylation_extractor module to extract methylation events in all the contexts, and the events were visualized in R 4.2.1 using ggplot2 R package (https://www.r-project.org/).

### shRNA-mediated knockdown and virus production.

shRNAs for mouse ATF4 and ZBTB1 were designed using the splashRNA program from MSKCC (33). The individual 97-mer hairpins were PCR amplified and cloned into an MSCV-miRE-SV40-GFP backbone using Xho1/ EcoR1 enzymes (34). Retroviruses were generated in 293T cells using pCL-Eco and VSV-G for packaging. Mouse leukemia cell lines were transduced with the retroviruses in the presence of polybrene (6 μg/mL) and sorted based on GFP expression.

### Data rigor and reproducibility.

Experiments were performed in biological and technical replicates to confirm data reproducibility. Western blots were performed twice or more with a representative blot shown in figures. Cell growth experiments were conducted and recorded in biological triplicate. Chemical inhibitors were assessed in a range of doses alone or in combination with other drugs to confirm observations.

### Statistics.

GraphPad Prism (v10.5.0) was used to plot data. Bar graphs were shown as mean ± standard deviation (SD) with at least 3 biological replicates. Statistical significance was determined using 2-way ANOVA. Specific additional information is listed in the figure legends. A *P* value of less than 0.05 was considered significant.

### Study approval.

All mouse experiments were performed in compliance with IU’s animal care and use protocols (IACUC protocol 26065).

### Data availability.

Values for all data points in graphs are reported in the Supporting Data Values file.

## Author contributions

RC, SS, and J Zhang wrote the manuscript. RC and SS designed and performed experiments and analyzed the results. KAS, CMF, SC, LL, MZ, and J Zhou performed experiments. UD, RK, and SB provided critical experimental reagents. HK, NAL, YF, and CZ conducted bioinformatics analysis. JF, RCW, and J Zhang designed experiments. J Zhang provided the conceptual idea and overall supervision of the project.

## Conflict of interest

RW serves as a scientific advisor to HiberCell and KS has served as a consultant for HiberCell.

## Funding support

This work is the result of NIH funding, in whole or in part, and is subject to the NIH Public Access Policy. Through acceptance of this federal funding, the NIH has been given a right to make the work publicly available in PubMed Central.

- NIH/NCI grant R01 CA244625 (to JZ).
- Riley Children Foundation (to JZ).
- NIH/NCI grant R01CA173852 (to RK).
- Department of Defense grant HT9425-24-1-0525 (to KS and RW).
- Ralph W. and Grace M. Showalter Research Trust (to RW).
- IU Simon Comprehensive Cancer Center (IUSCCC) Pilot Funding Award (to RC and SS).
- Adam W. Herbert Fellowship (to RC).
- NIH grant P30CA082709 (to the IUSCCC).
- NIH grant P30CA023168 (to the Purdue University Center for Cancer Research).

## Supplementary Material

Supplemental data

Unedited blot and gel images

Supporting data values

## Version 1 06/04/2026

In-Press Preview

## Version 2 07/22/2026

Electronic publication
