# Supplementary material for: Direct pharmacological targeting of asparagine synthetase to overcome resistance to L-asparaginase in ALL therapy
Source: JCI Insight. 2026 Jun 4;11(14):e203777. doi: 10.1172/jci.insight.203777 (PMC13461159; doi:10.1172/jci.insight.203777)
Supplement: Supplementary file 1 [file jciinsight-11-203777-s116.pdf]

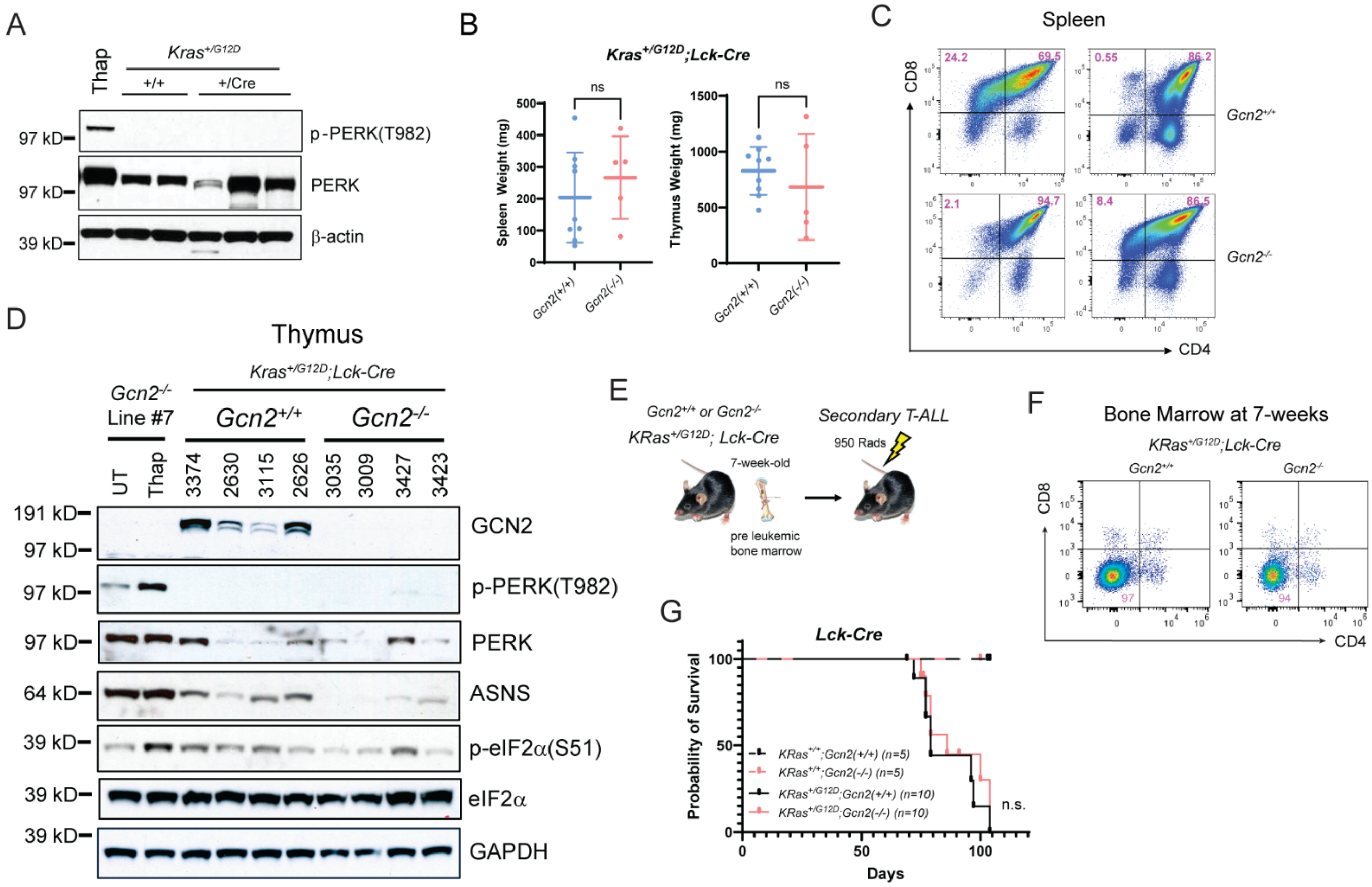

Supplemental Figure 1. GCN2 is not required for leukemogenesis. (A) Western blot analysis was used to assess p-PERK (T982) in thymus from *Kras*^{G12D} mice, with (n=3) or without (n=2) Cre. Mouse leukemia cells treated with thapsigargin (1 µM for 3 hours) were used as a control. **(B)** Spleen and thymus weights from *KRas*^{G12D};*Lck-Cre* animals that are either *Gcn2*$^{+/+}$ or *Gcn2*$^{-/-}$. (**C**) Representative CD4/CD8 profiles from the spleen tissues in panel **B**. **(D)** Western Blot measuring GCN2, p-PERK (T982), PERK, p-eIF2α(S51), eIF2α, and ASNS protein expression in thymus tissues from *Gcn2*$^{+/+}$ and *Gcn2*$^{-/-}$ leukemia mice (n=4 for each genotype). (**E**) Schematic establishment of a secondary T-ALL, driven by pre-leukemic bone marrow cells from *KRas*^{G12D};*Lck-Cre* mice transplanted into lethally irradiated recipient mice. (**F**) Representative CD4/CD8 profiles of pre-leukemic bone marrow cells from *KRas*^{G12D};*Lck-Cre* mice that are either *Gcn2*$^{+/+}$ or *Gcn2*$^{-/-}$. (**G**) Kaplan-Meier survival curves were plotted for the secondary transplant model illustrated in panel (**E**). *Kras wild-type* bone marrow cells were used as controls. Survival was monitored over a 120-day period after pre-leukemic transplantation. Statistical significance was determined using the log-rank (Mantel-Cox) test.

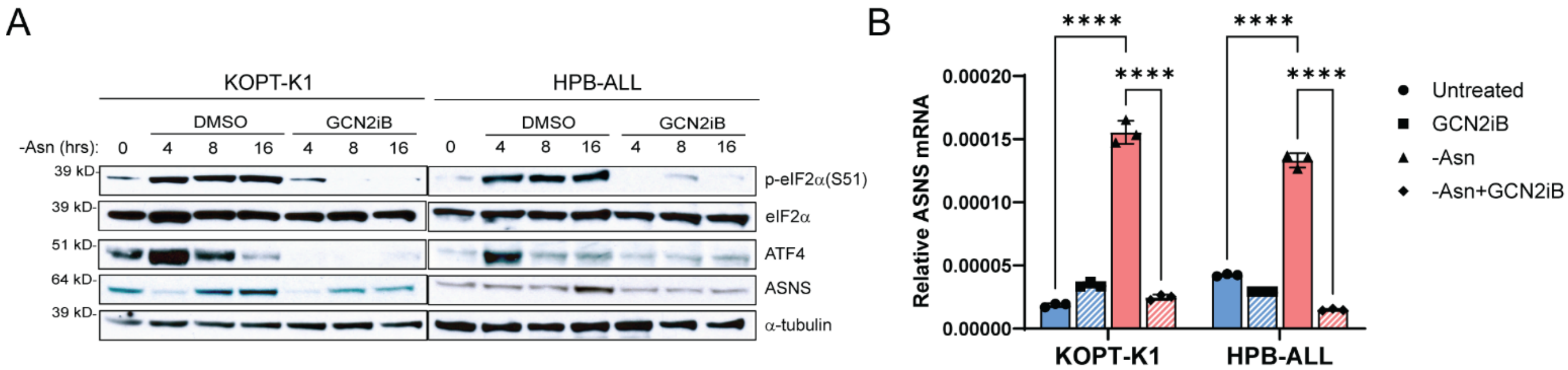

Supplemental Figure 2. GCN2iB suppresses ASNS induction in KOPT-K1 and HPB-ALL cells following asparagine depletion. (A) KOPT-K1 and HPB-ALL cells were grown in asparagine deficient media with or without GCN2iB over a time course of 0, 4, 8 and 16 hours. ATF4, ASNS and p-eIF2α(S51) were measured by Western blot analysis. **(B)** mRNA from the 16-hour time point in panel **(A)** was collected for qPCR analysis of ASNS. Statistical significance was determined using two-way ANOVA. **** $p<0.0001$.

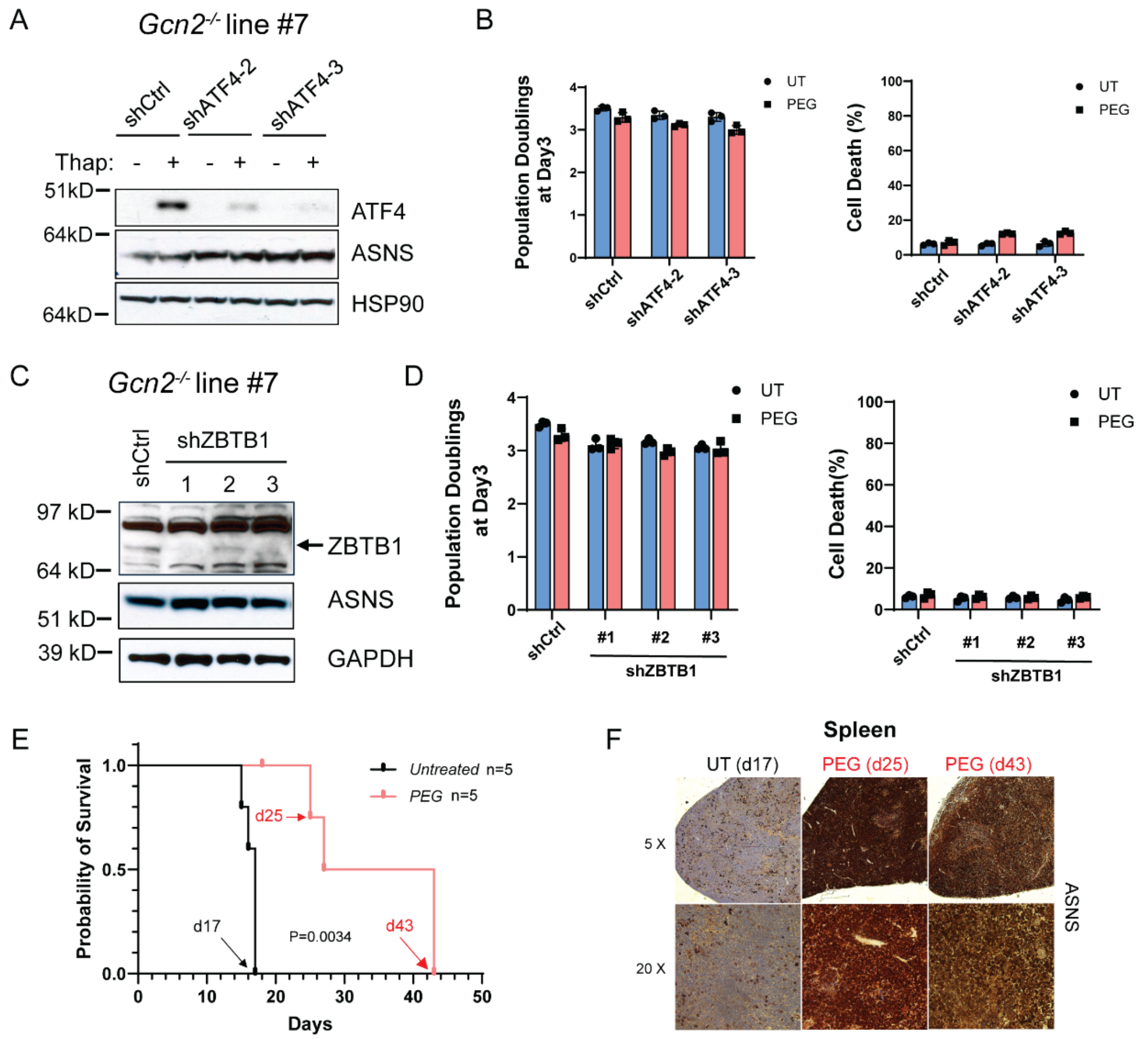

Supplemental Figure 3. GCN2-independent mechanisms contribute to L-asparaginase resistance via ASNS induction. (**A**) shRNA-mediated knockdown of ATF4 in the *Gcn2*$^{-/-}$ line #7 effectively depletes ATF4 protein following thapsigargin treatment (1μM for 3 hours). (**B**) Cells in panel (**A**) were treated with PEG for 3 days. Population doublings and cell death were recorded. (**C**) shRNA-mediated knockdown of ZBTB1 in the *Gcn2*$^{-/-}$ line #7 effectively reduces ZBTB1 protein with two hairpins at least. (**D**) Cells in panel (**C**) were treated with PEG for 3 days. Population doublings and cell death were recorded. (**E**) Another *Gcn2*$^{-/-}$ leukemia model was established as shown in **Figure 4C**. PEG was given two doses on day 10 & 34 post-transplantation. Kaplan-Meier curve comparing survival between the untreated and PEG-treated mice were plotted. Statistical comparison between the two groups was done using the log-rank (Mantel-Cox) test. (**F**) IHC staining of ASNS from the spleen of mice euthanized on day 17 (untreated), 25 (PEG), and 43 (PEG). Representative microscopy images were obtained at 5X or 20X magnification as indicated.

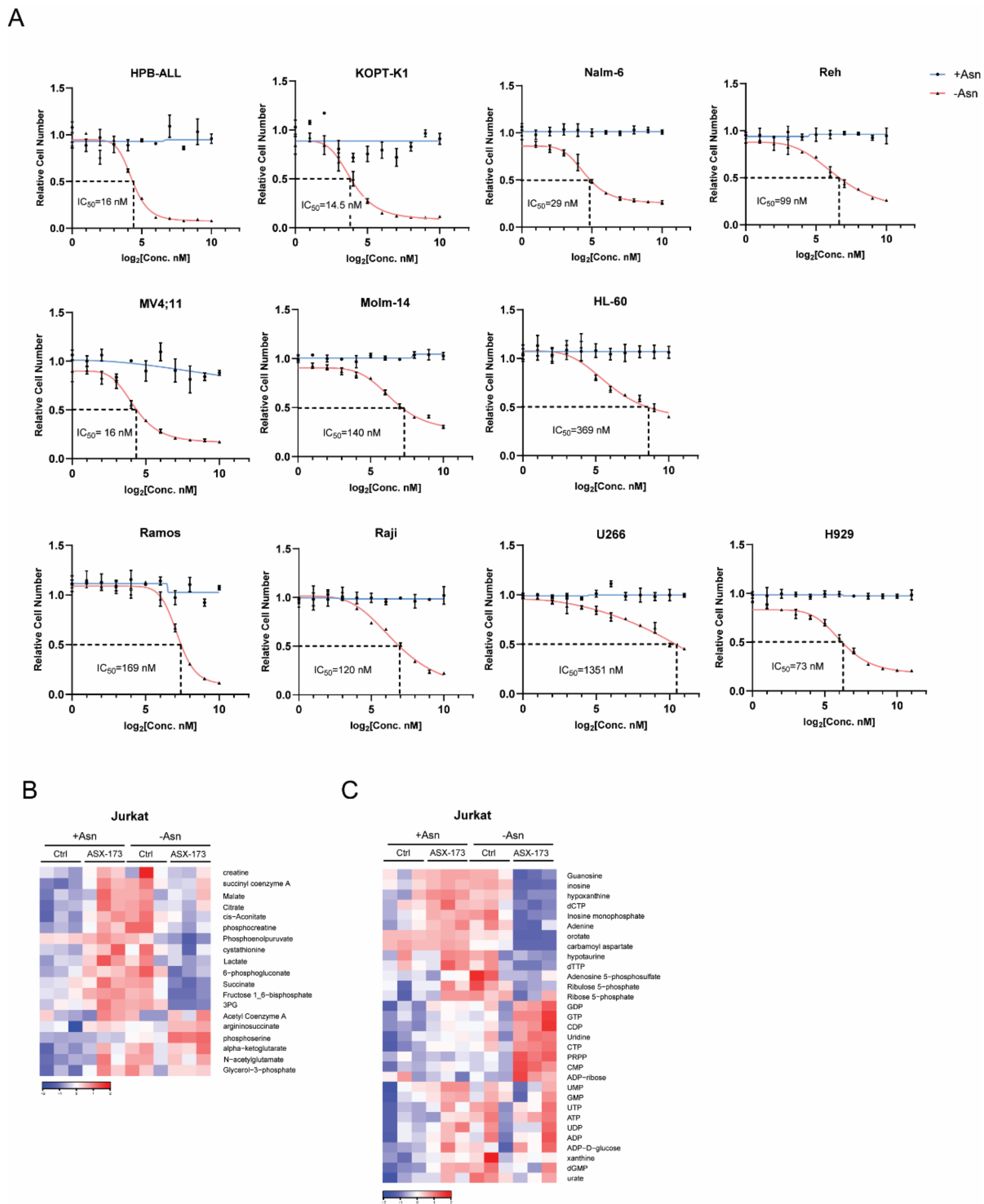

Supplemental Figure 4. ASX-173 overcomes resistance to asparagine depletion in ASNS-high blood cancer cells. (**A**) Relative numbers of hematopoietic cancer cells in **Figure 5C** cultured in the presence or absence of asparagine for 2 days with a series of concentration of ASX-173, as indicated on the x-axis. (**B, C**) Heatmap analysis of metabolites (central carbon metabolites in **B**, nucleotide and nucleotide derivatives in **C**) from Jurkat cells treated as **Figure 5F**.

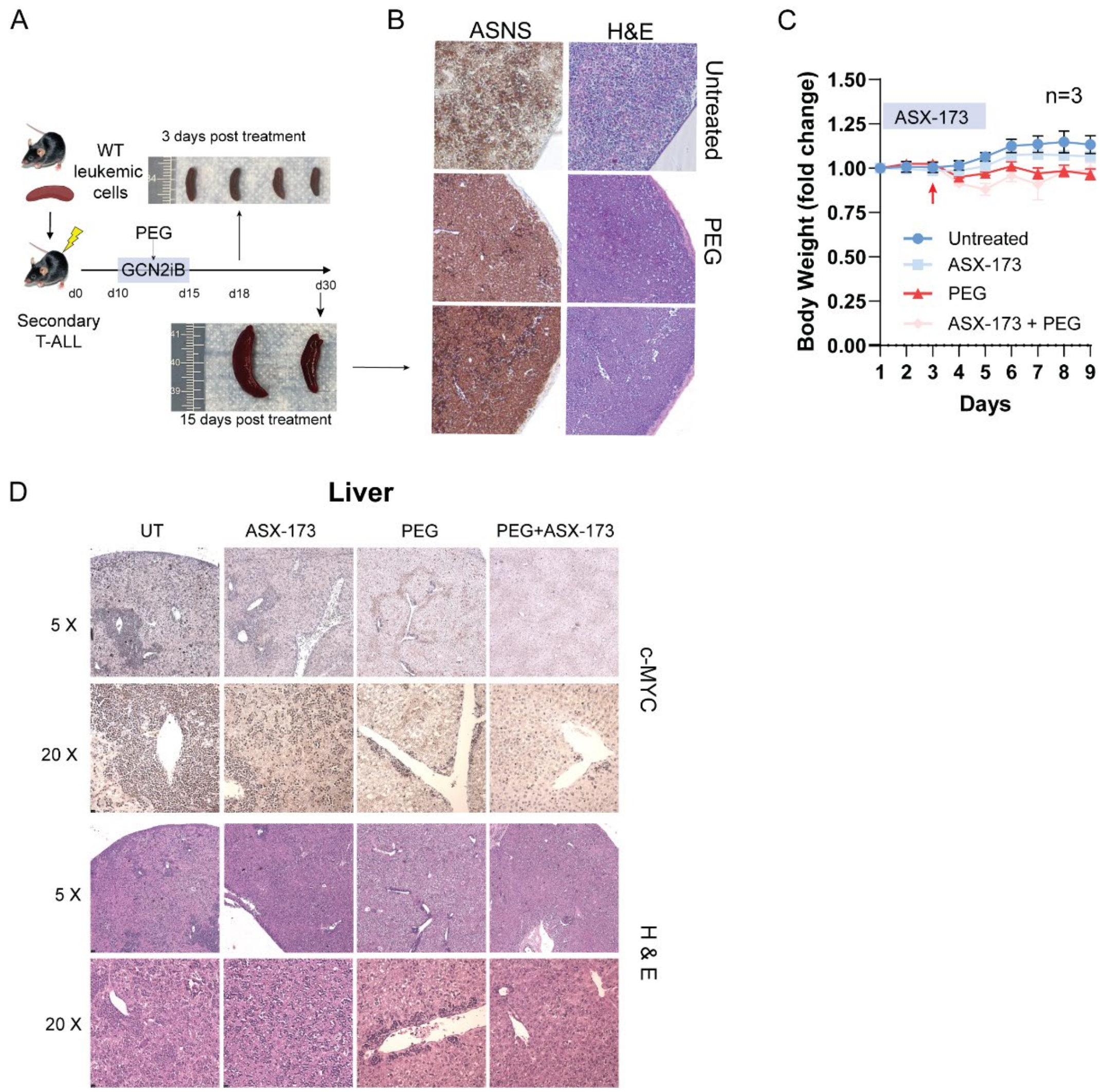

Supplemental Figure 5. ASX-173 reduces leukemic burden in ALL cells relapsed from GCN2iB and L-asparaginase treatment. (**A**) Extended schematic illustration of leukemic model in **Figure 3G**. Mice from the GCN2iB plus PEG combo treatment group were monitored closely after day 18 for continued therapeutic assessment. Mice relapsed 15 days after treatment. Top panel of spleen images are from **Figure 3G**. Bottom panel of spleen are from mice 15 days post treatment. (**B**) IHC staining of ASNS (left) in the spleens from panel (**A**). Right panels were stained for H&E. (**C**) Body weight fold change of mice during treatment. ASX-173 was given between day 1 and 5. PEG was given on day 3. (**D**) Liver tissues from the mice in **Figure 6A** were subjected to IHC staining for c-MYC and H&E staining. Representative microscopy images were obtained at 5X or 20X magnification as indicated.

Supplementary Table 1

Human cell lines	Source	RRID	Comments
Jurkat	ATCC	CVCL_0065	T-ALL from PB of 14 yr old male
HPB-ALL	Gift from Hui Feng lab	CVCL_1820	T-ALL from PB of 14 yr old male
KOPT-K1	Gift from Hui Feng lab	CVCL_4965	T-ALL from PB of 6 yr old male
Nalm-6	DSMZ	CVCL_0092	B-ALL from of 19 yr old male
Reh	ATCC	CVCL_1650	B-ALL from of 15 yr old female
RS4;11	ATCC	CVCL_0093	B-ALL from of 32 yr old female
Ramos	ATCC	CVCL_0597	Burkitt's Lymphoma from 3 yr old male
Raji	ATCC	CVCL_0511	Burkitt's Lymphoma from 11 yr old male
MV4;11	ATCC	CVCL_0064	AML from 10 yr old male
MOLM-14	Gift from Reuben Kapur lab	CVCL_7916	AML from 20 yr old male

HL-60	Gift from Reuben Kapur lab	CVCL_0027	AML from 36 yr old female
H929	Gift from Ngoc Tung Tran lab	CVCL_1600	Multiple myeloma from 62 yr old female
U266	Gift from Ngoc Tung Tran lab	CVCL_0566	Multiple myeloma from 53 yr old male

Supplementary Table 2

**Antibody**	**Source**	**Catalog #**
Rabbit Polyclonal ASNS	Proteintech	14681-1-AP
Rabbit monoclonal ATF4 (D4B8)	Cell Signaling Technology	#11815
Mouse monoclonal β-actin (AC-74)	Sigma-Aldrich	A2228
Mouse monoclonal α-tubulin (DM1A)	Sigma-Aldrich	T9026
Mouse monoclonal HSP90 α/β	Santa Cruz Biotechnology	Sc-13119
Rabbit monoclonal c-MYC (D84C12)	Cell Signaling Technology	#5605
Rabbit monoclonal PARP (46D11)	Cell Signaling Technology	#9532

Rabbit polyclonal Phospho GCN2 (Thr899)	Cell Signaling Technology	#94668
Rabbit Monoclonal GCN2 (E7I8W)	Cell Signaling Technology	#63675
Rabbit polyclonal GCN2	Cell Signaling Technology	#3302
Rabbit Polyclonal eIF2α	Cell Signaling Technology	#9722
Rabbit monoclonal Phospho eIF2α(Ser51) (D9G8) XP(R)	Cell Signaling Technology	#3398
Rabbit monoclonal NOTCH1 (D1E11)	Cell Signaling Technology	#3608
Rabbit Phospho PERK (T982)	Dr. Ronald C. Wek, Indiana University	(in-laboratory)
Rabbit PERK	Cell Signaling Technology	#3192
GAPDH	Cell Signaling Technology	#97166
Purified anti-mouse CD16/32 Antibody	BioLegend	#101302
PerCP/Cyanine5.5 anti-mouse CD45.1 Antibody	BioLegend	#110727
FITC anti-mouse CD45.2 Antibody	BioLegend	#109806
PerCP/Cyanine5.5 anti-mouse CD45 Antibody	BioLegend	#103132

PE/Cyanine7 anti-mouse CD3 Antibody	BioLegend	#100220
Brilliant Violet 605™ anti-mouse CD8a Antibody	BioLegend	#100744
Brilliant Violet 711™ anti-mouse CD4 Antibody	BioLegend	#100557
PE anti-mouse/human CD44 Antibody	BioLegend	#103008

Supplementary Table 3

**Oligoes**	**Sequence**
Human & Mouse 18s rRNA qPCR primers	CTGGATACCGCAGCTAGGAA3<br>GAATTTCACCTCTAGCGGCG
Mouse ASNS qPCR primers	GTTGCGTCTGTGGAAATGGT<br>AGAGTTTCTCCACGCTGTCA
Human ASNS qPCR primers	CTCTCCAGACATTTGCAATTGG<br>GCCACCTTTCTAGCAGCCAGTA
Primers to amplify mouse Asns promoter for bisulfite sequencing	AGTATTTTAAGGAATAGAAGTAGAATATTT<br>CCAAAAAAAATAAAACAAAAACAAC
mASNS bisulfite forward Barcode 1 (*Gcn2*$^{-/-}$ #2)	ACACTCTTTCCCTACACGACGCTCTTCCGATCTAAGTAGAG<br>AGTATTTTAAGGAATAGAAGTAGAATATTT
mASNS bisulfite forward Barcode 2 (*Gcn2*$^{-/-}$ #4)	ACACTCTTTCCCTACACGACGCTCTTCCGATCTACACGATC<br>AGTATTTTAAGGAATAGAAGTAGAATATTT
mASNS bisulfite forward Barcode 3 (*Gcn2*$^{-/-}$ D)	ACACTCTTTCCCTACACGACGCTCTTCCGATCTTATGCGTTA<br>GTATTTTAAGGAATAGAAGTAGAATATTT
mASNS bisulfite forward Barcode 4 (*Gcn2*$^{-/-}$ #7)	ACACTCTTTCCCTACACGACGCTCTTCCGATCTCGCGCGGT<br>AGTATTTTAAGGAATAGAAGTAGAATATTT
mASNS bisulfite reverse adaptor	GACTGGAGTTCAGACGTGTGCTCTTCCGATCTCCAAAAAAA<br>ATAAAACAAAAACAAC

shRNA 1 for mATF4	TGCTGTTGACAGTGAGCGAACAAAGATACCTTCGAGTTAAT AGTGAAGCCACAGATGTATTAACTCGAAGGTATCTTTGTCT GCCTACTGCCTCGGA
shRNA 2 for mATF4	TGCTGTTGACAGTGAGCGATCTAGTCCAAGAGACTAATAAT AGTGAAGCCACAGATGTATTATTAGTCTCTTGGACTAGAGTG CCTACTGCCTCGGA
shRNA 1 for mZBTB1	TGCTGTTGACAGTGAGCGCCCAGAAAAAGCAGTTATTTAAT AGTGAAGCCACAGATGTATTAAATAACTGCTTTTTCTGGATG CCTACTGCCTCGGA
shRNA 2 for mZBTB1	TGCTGTTGACAGTGAGCGAACCGACAATGATGAATTAGAAT AGTGAAGCCACAGATGTATTCTAATTCATCATTGTCGGTGTG CCTACTGCCTCGGA
shRNA 3 for mZBTB1	TGCTGTTGACAGTGAGCGCGTGGAGCAAAGTTTAATTTGAT AGTGAAGCCACAGATGTATCAAATTAAACTTTGCTCCACATG CCTACTGCCTCGGA
PCR primers to amplify shRNA for cloning	TACAATACTCGAGAAGGTATATTGCTGTTGACAGTGAGCG<br>TTAGATGAATTCTAGCCCCTTGAAGTCCGAGGCAGTAGGCA
