# Supplementary material for: Direct pharmacological targeting of asparagine synthetase to overcome resistance to L-asparaginase in ALL therapy
Source: JCI Insight. 2026 Jun 4;11(14):e203777. doi: 10.1172/jci.insight.203777 (PMC13461159; doi:10.1172/jci.insight.203777)

Figure 1E

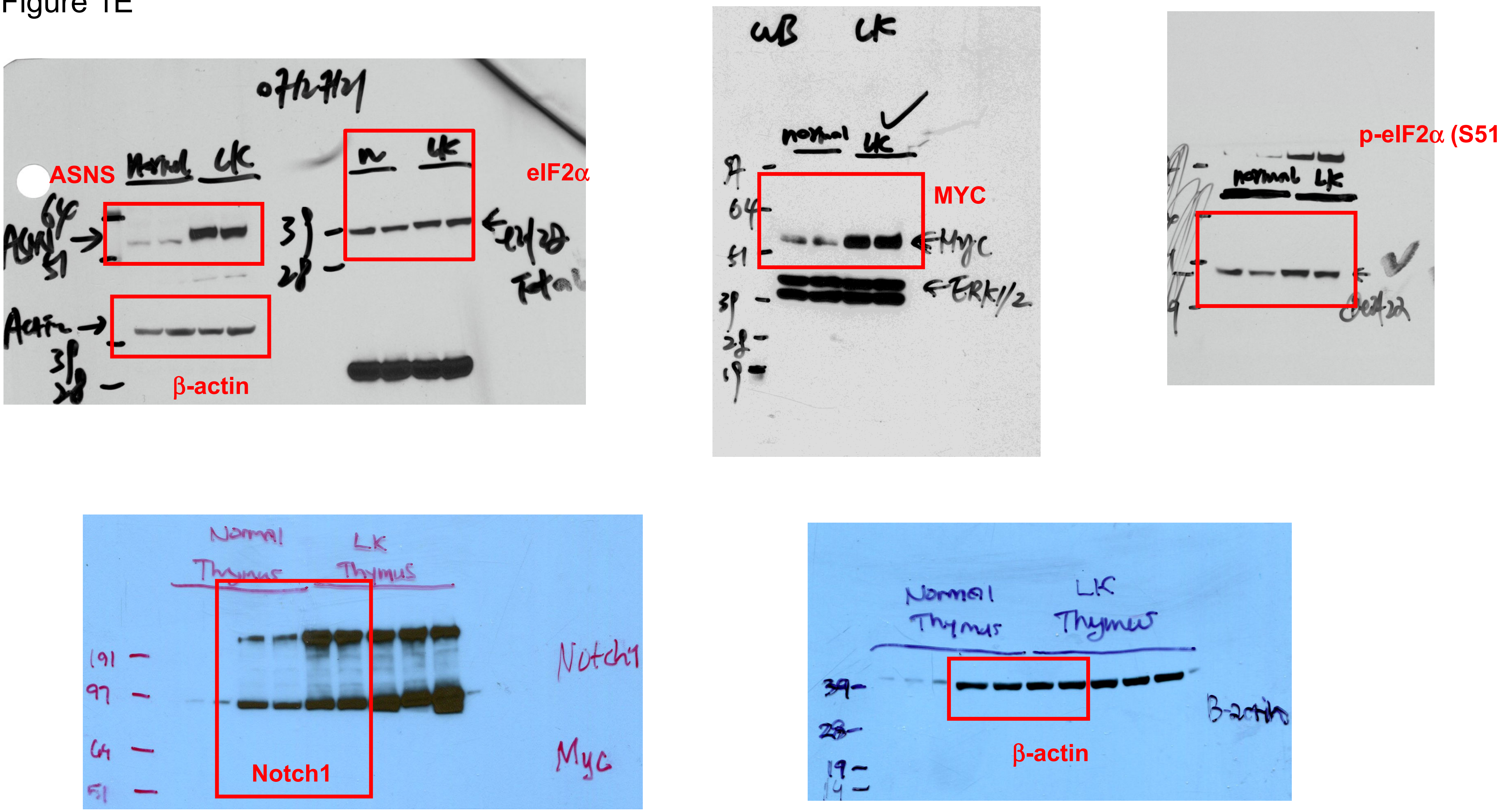

ASNS
β-actin
eIF2α
MYC
p-eIF2α (S51)
Normal Thymus
LK Thymus
Notch1
Myc
β-actin

Figure 1E

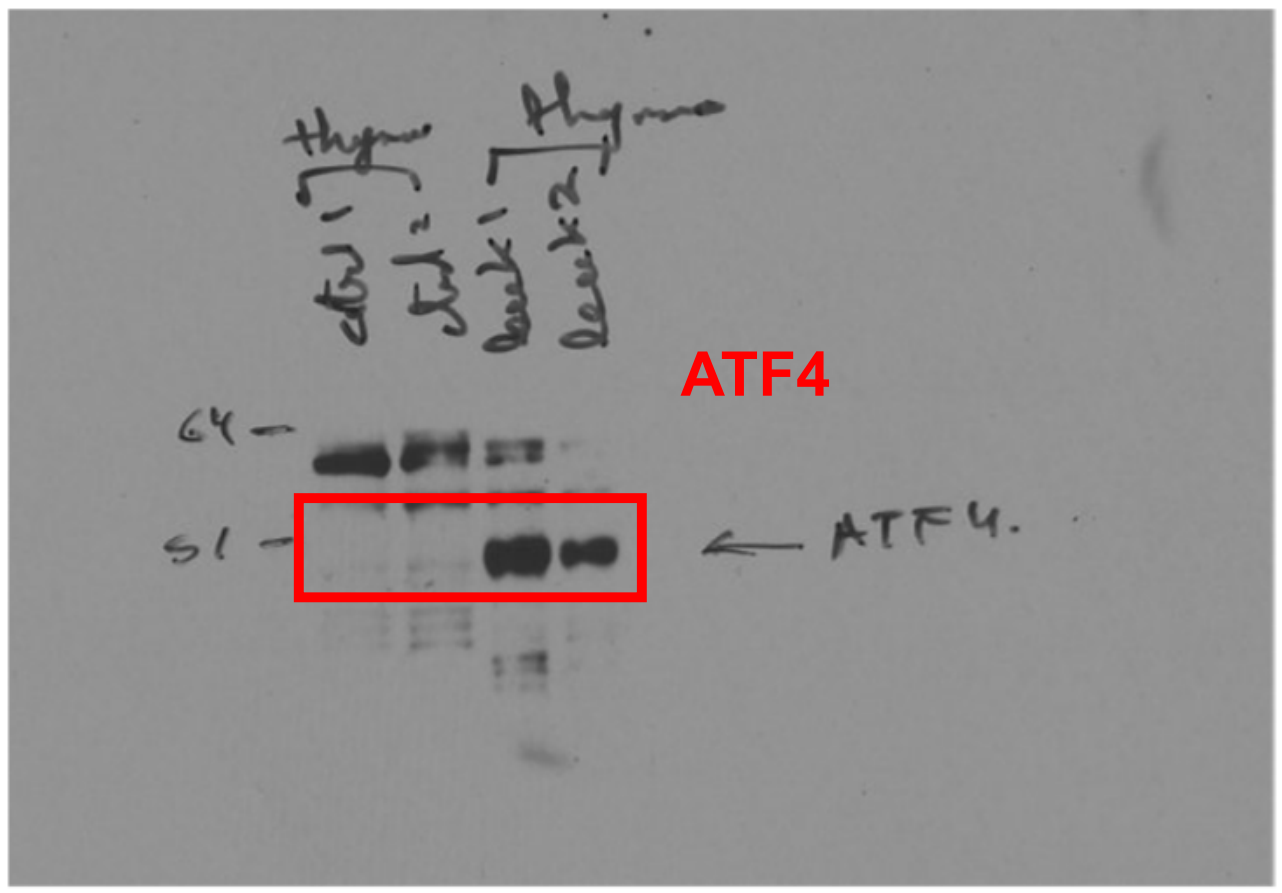

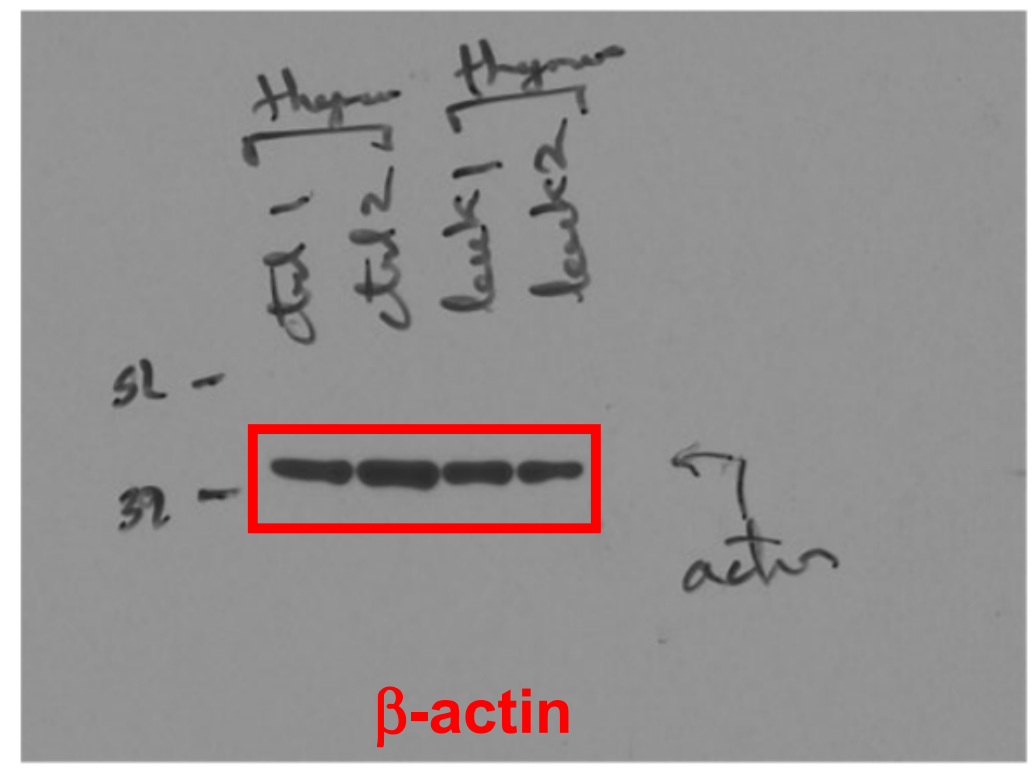

Figure 2D

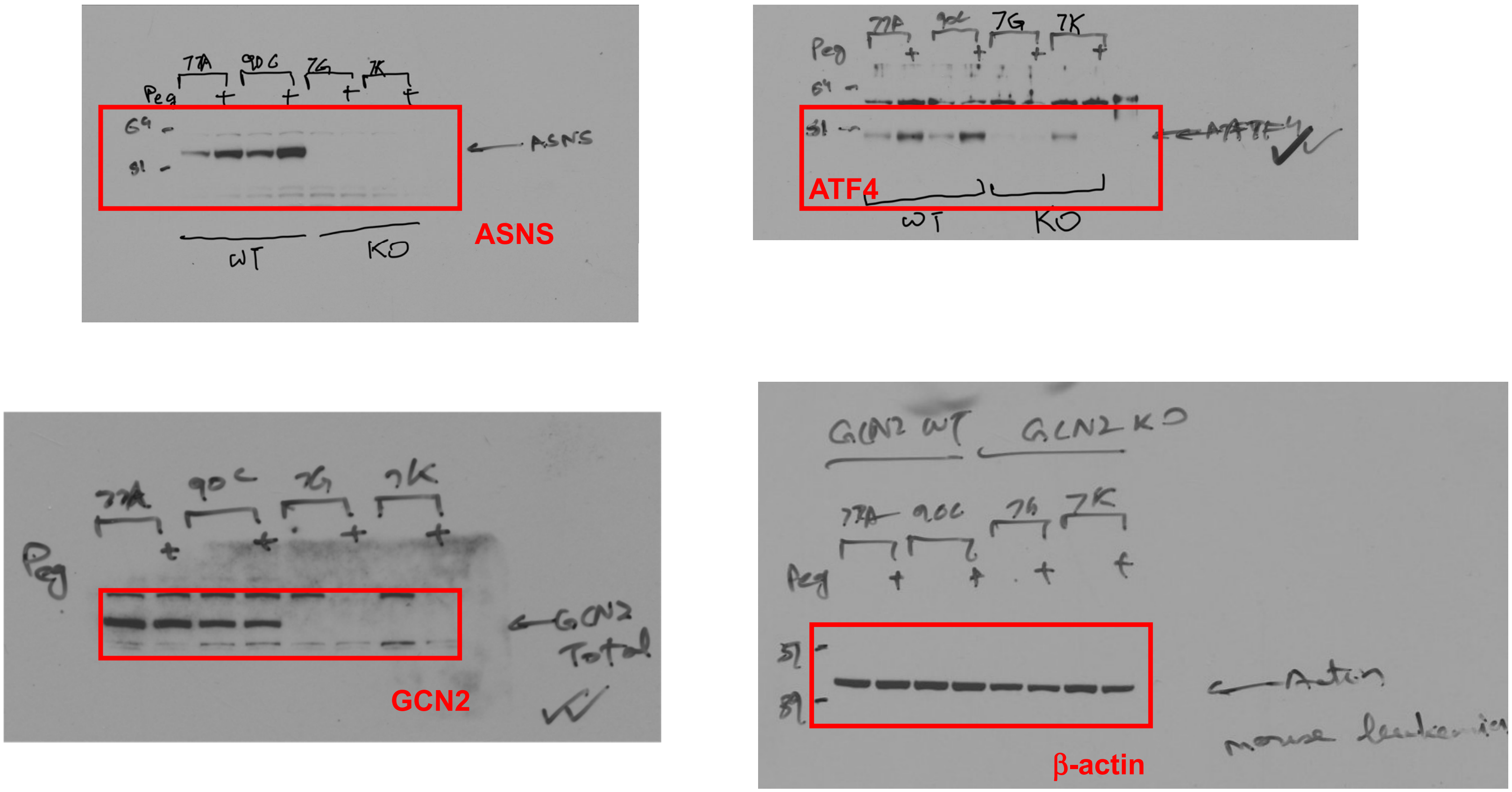
ASNS
WT
KO
ATF4
WT
KO
GCN2
GCN2 Total
Peg
GCN2 WT
GCN2 KO
Actin
β-actin

Figure 3B

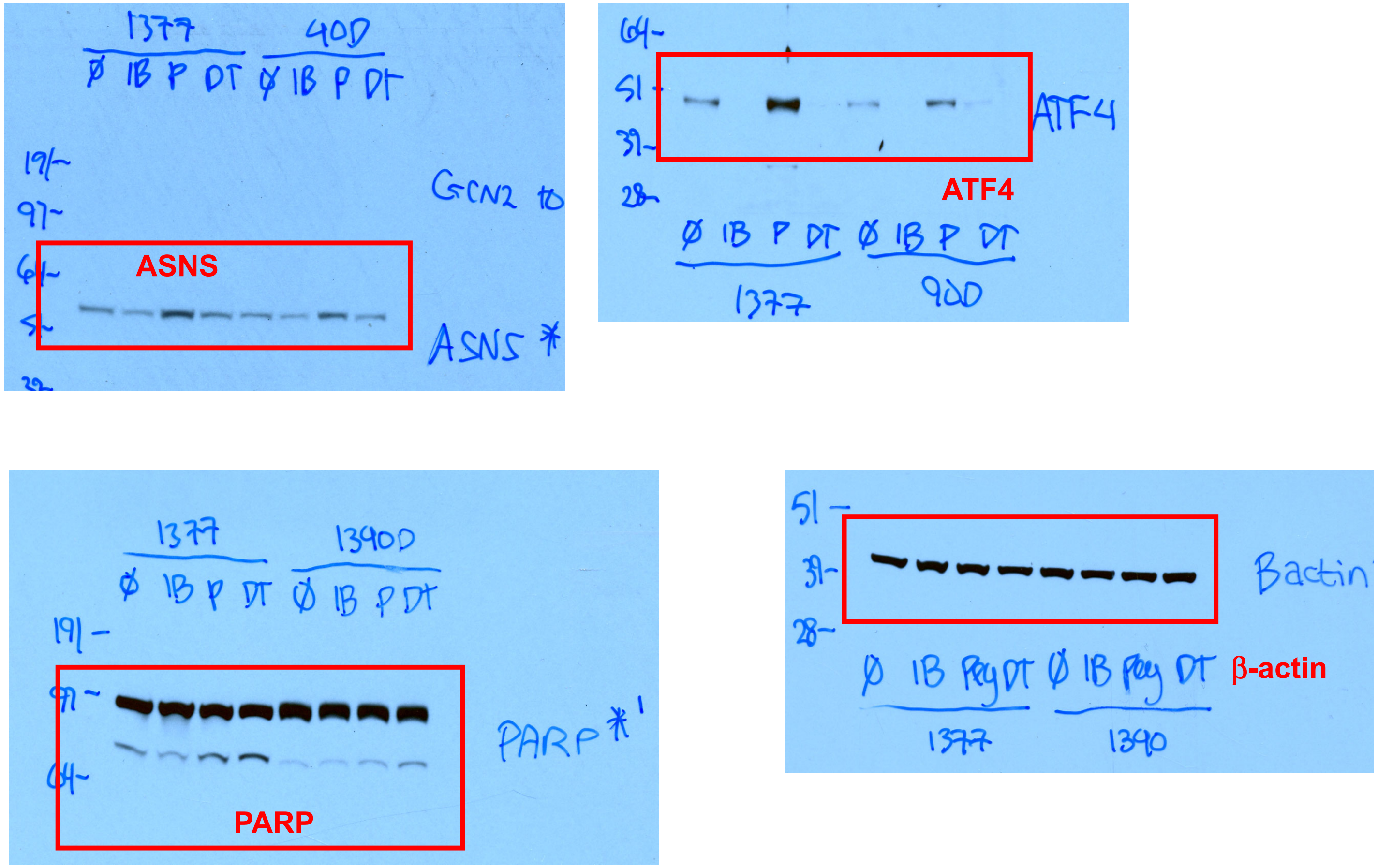

Figure 3D

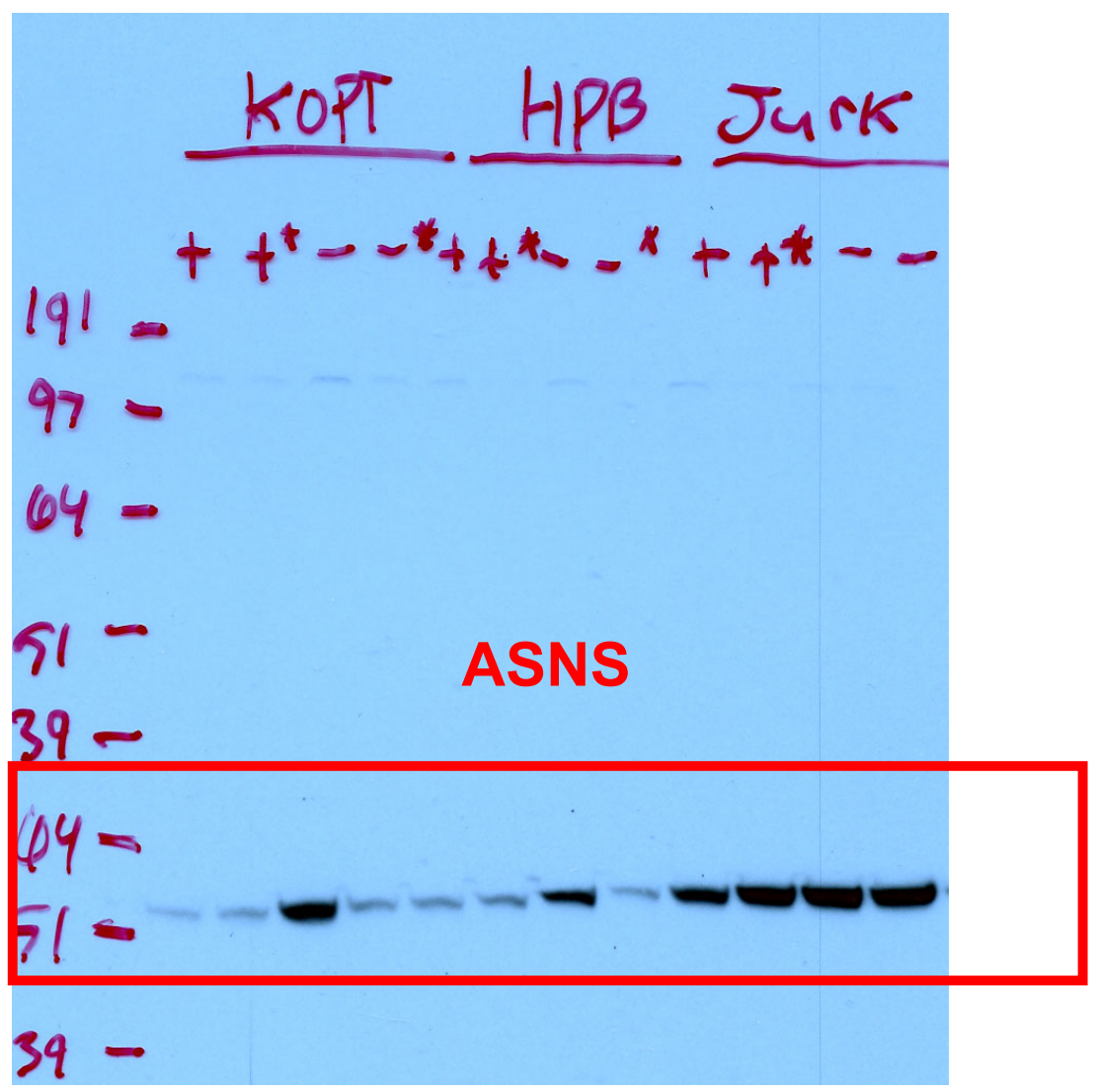
KOPT
HPB
Jurk
191
97
64
51
39
ASNS
64
51
39

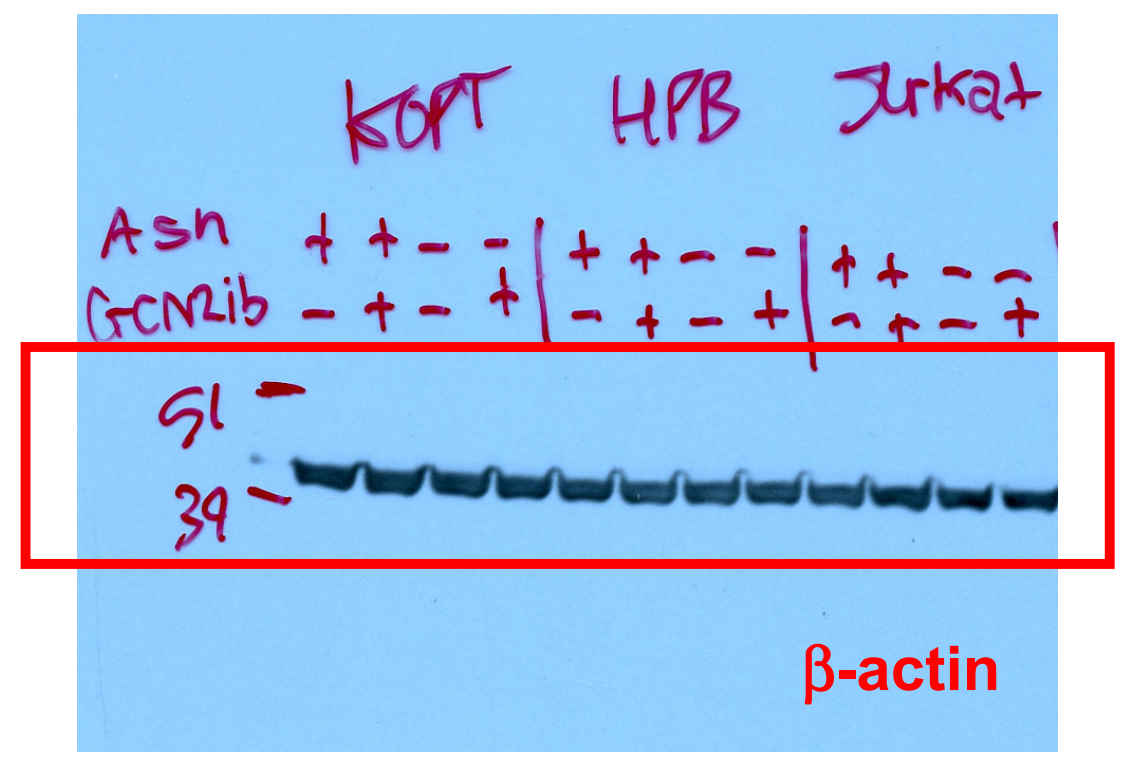
KOPT
HPB
Jurkat
Asn
GCN2ib
51
39
β-actin

Figure 4A (Left)

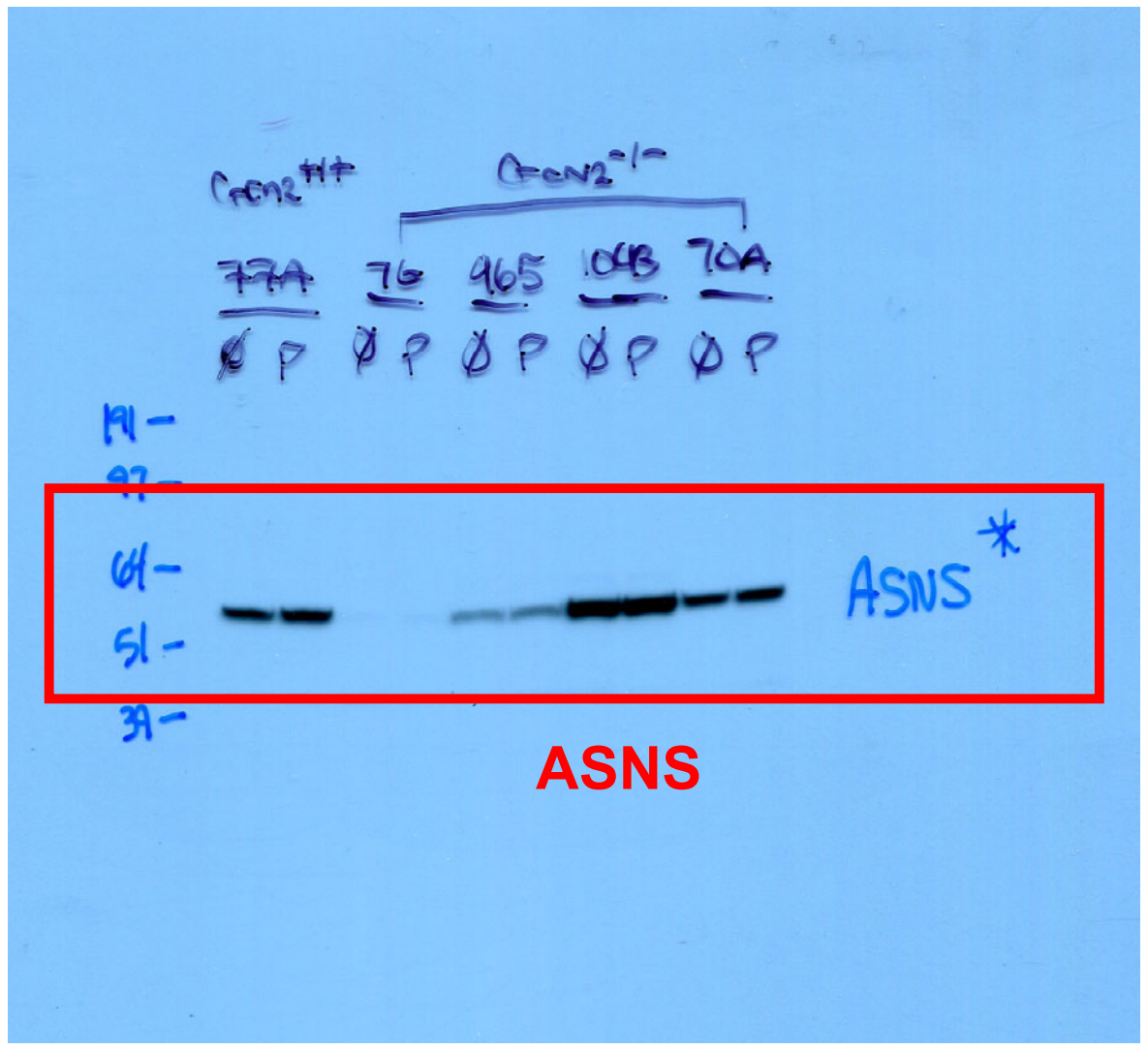

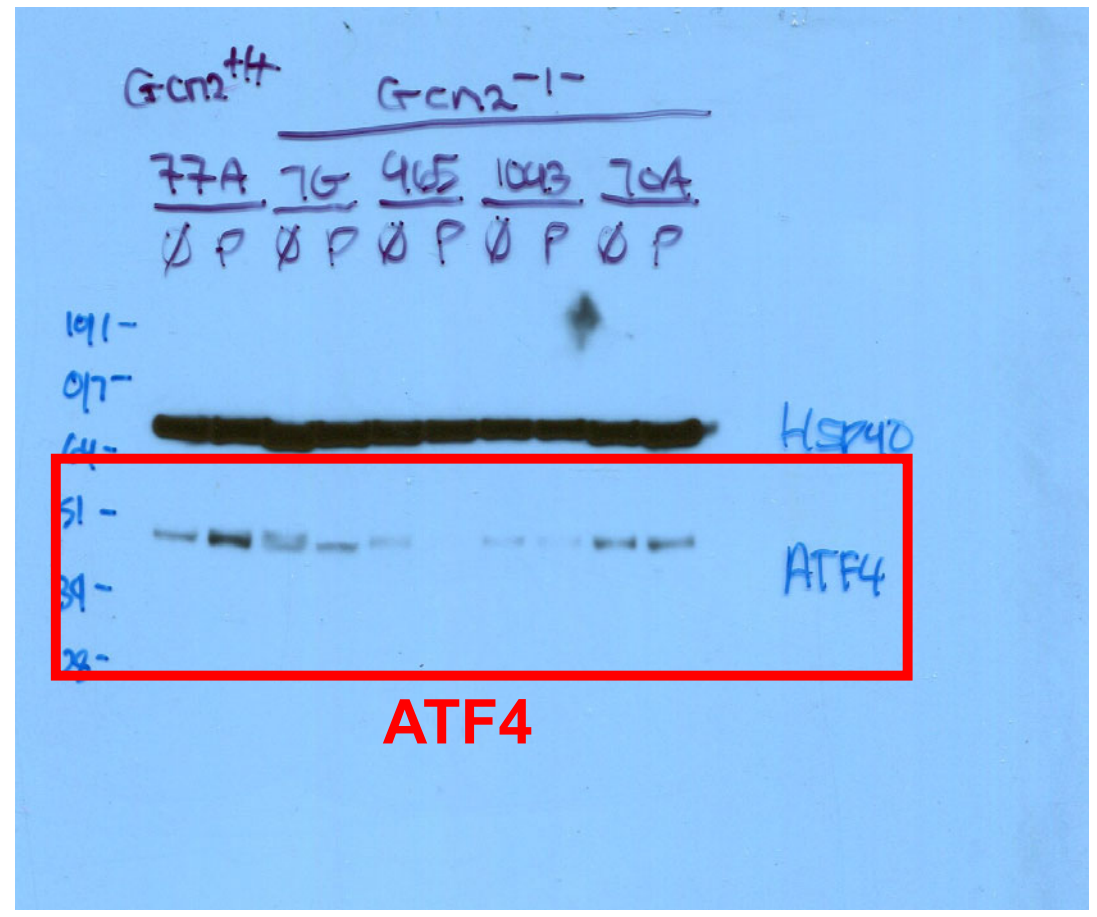

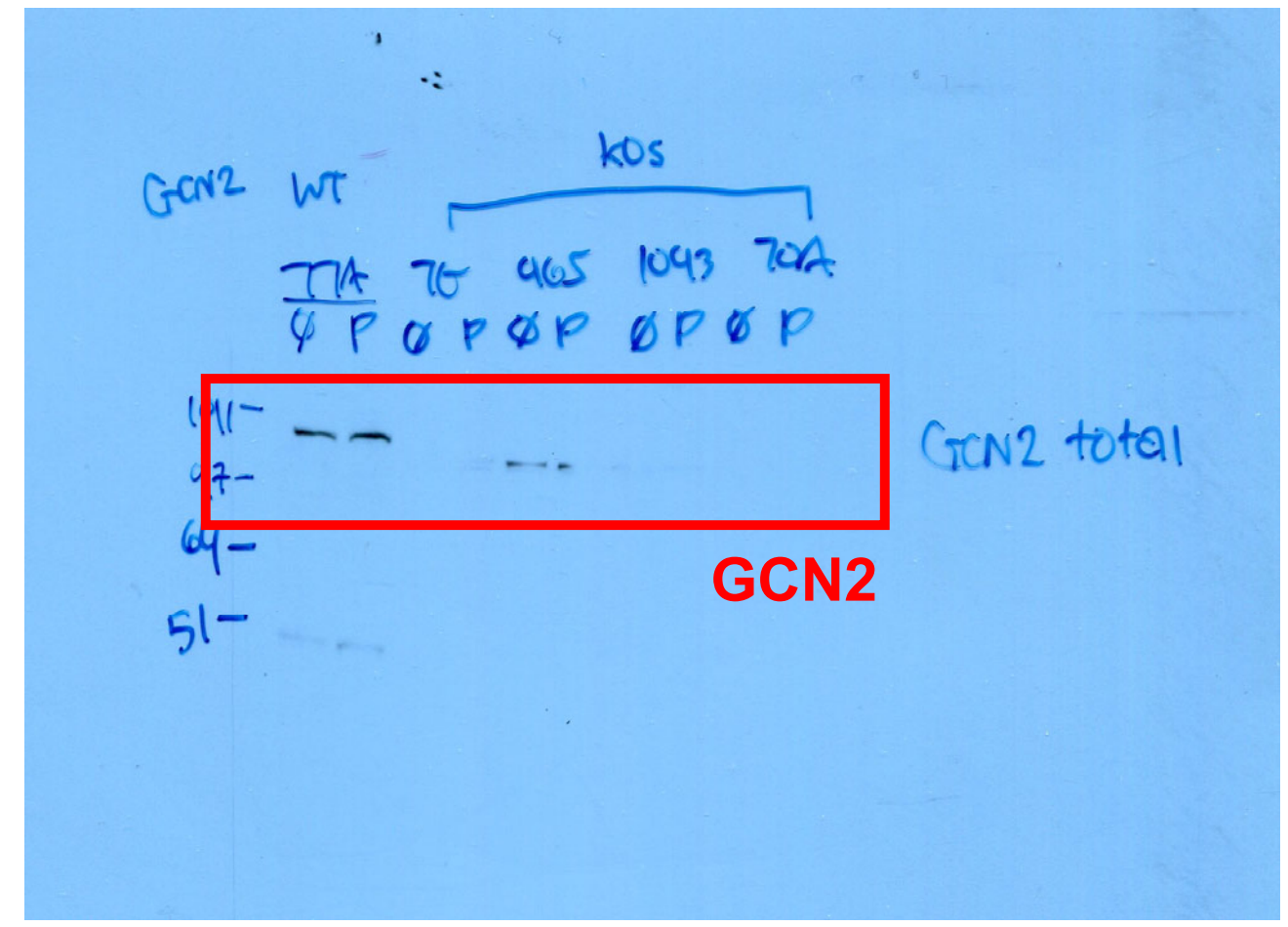

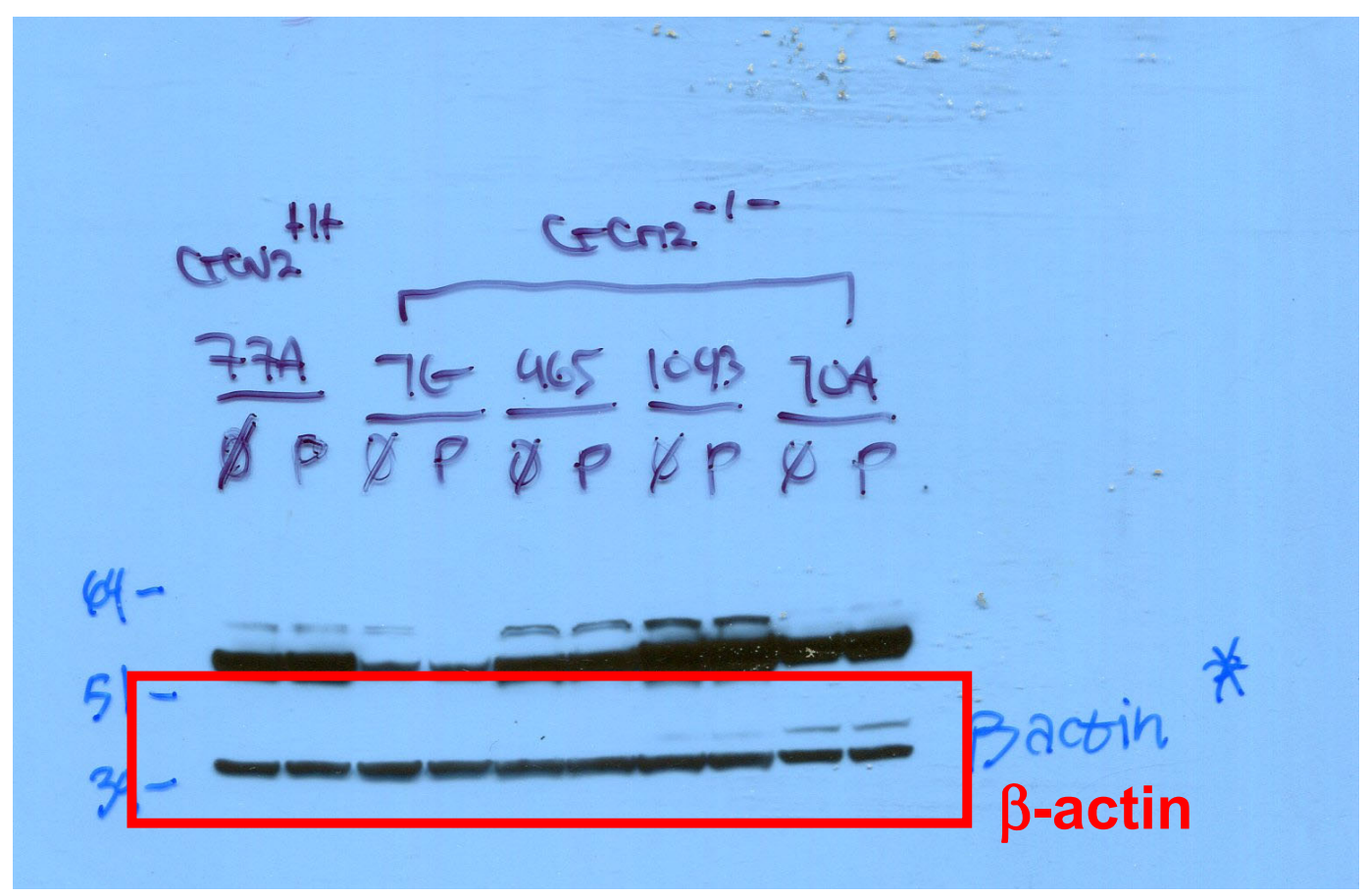

Figure 4A (Right)

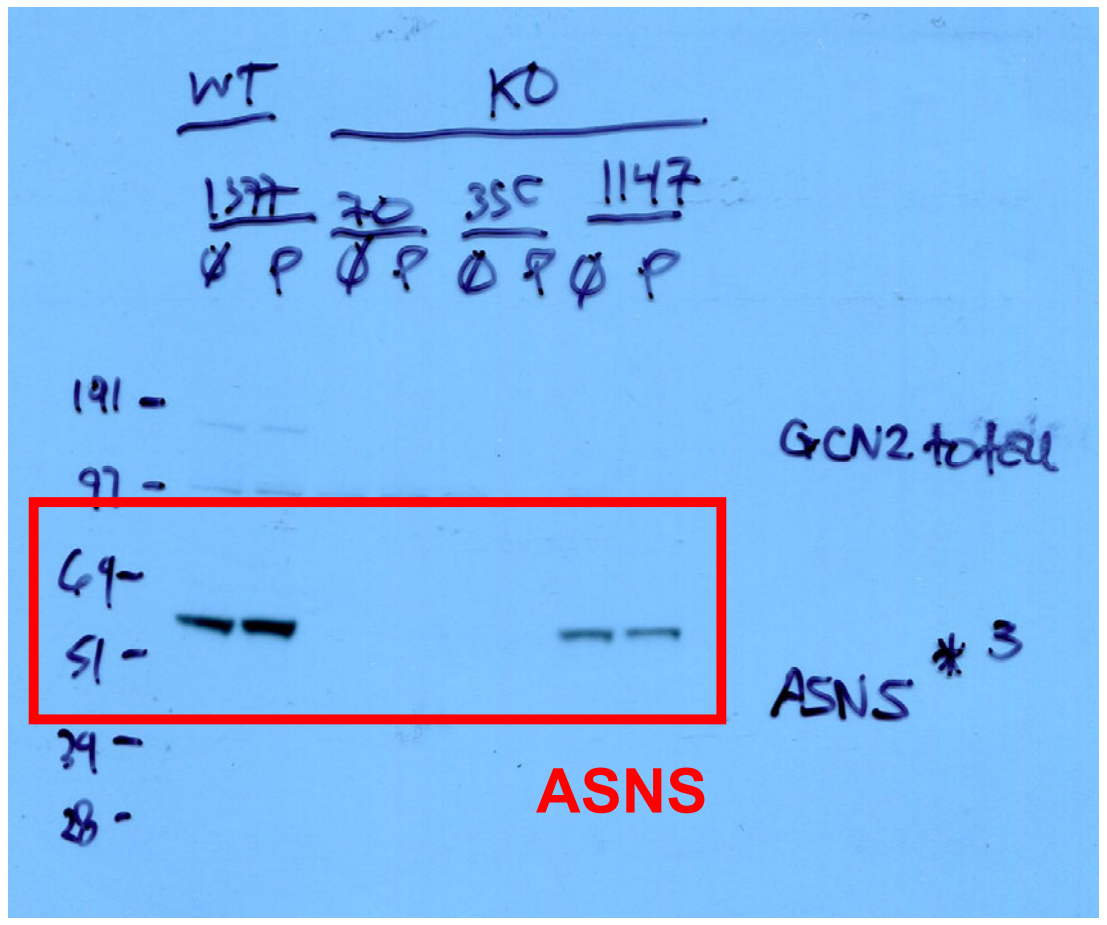

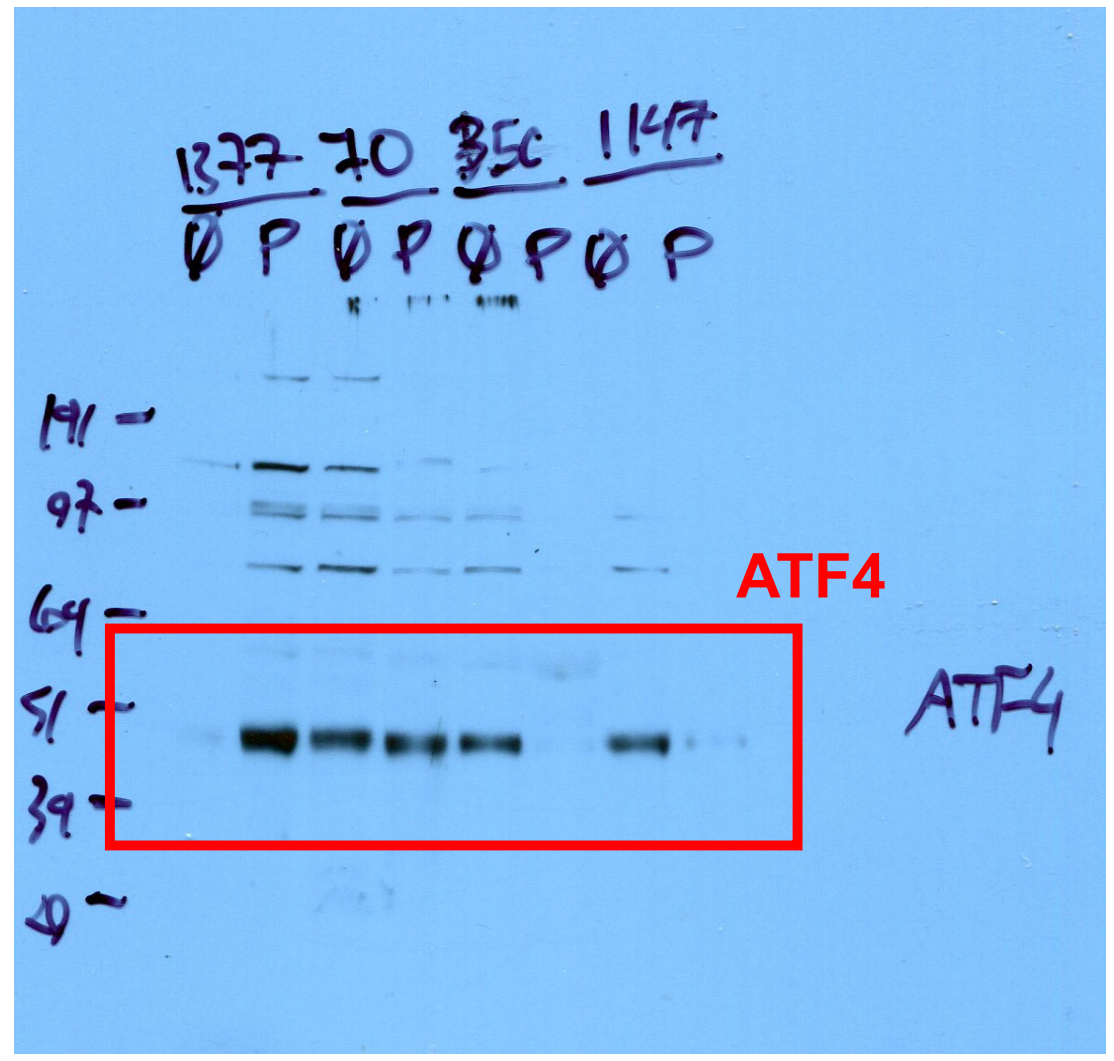

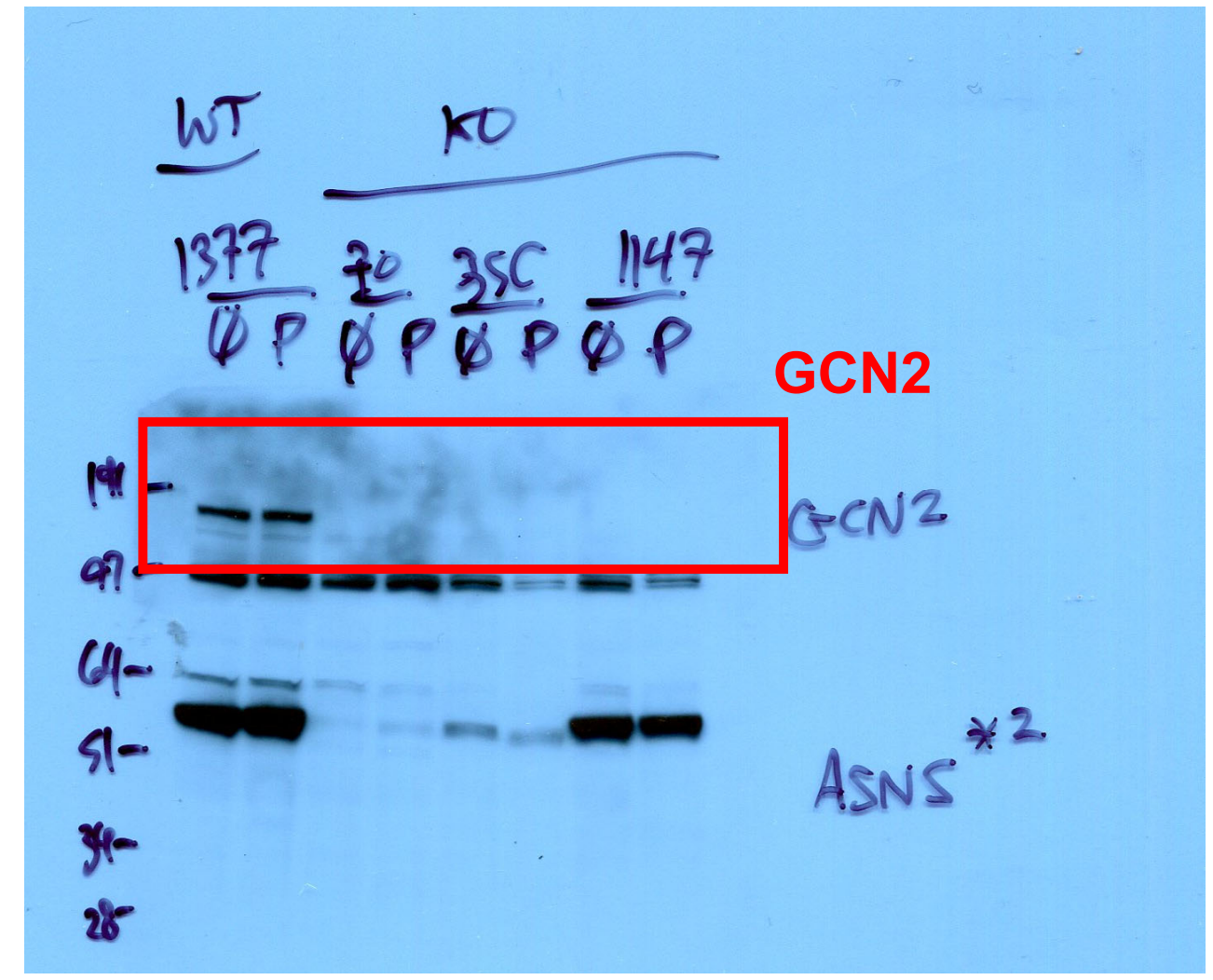

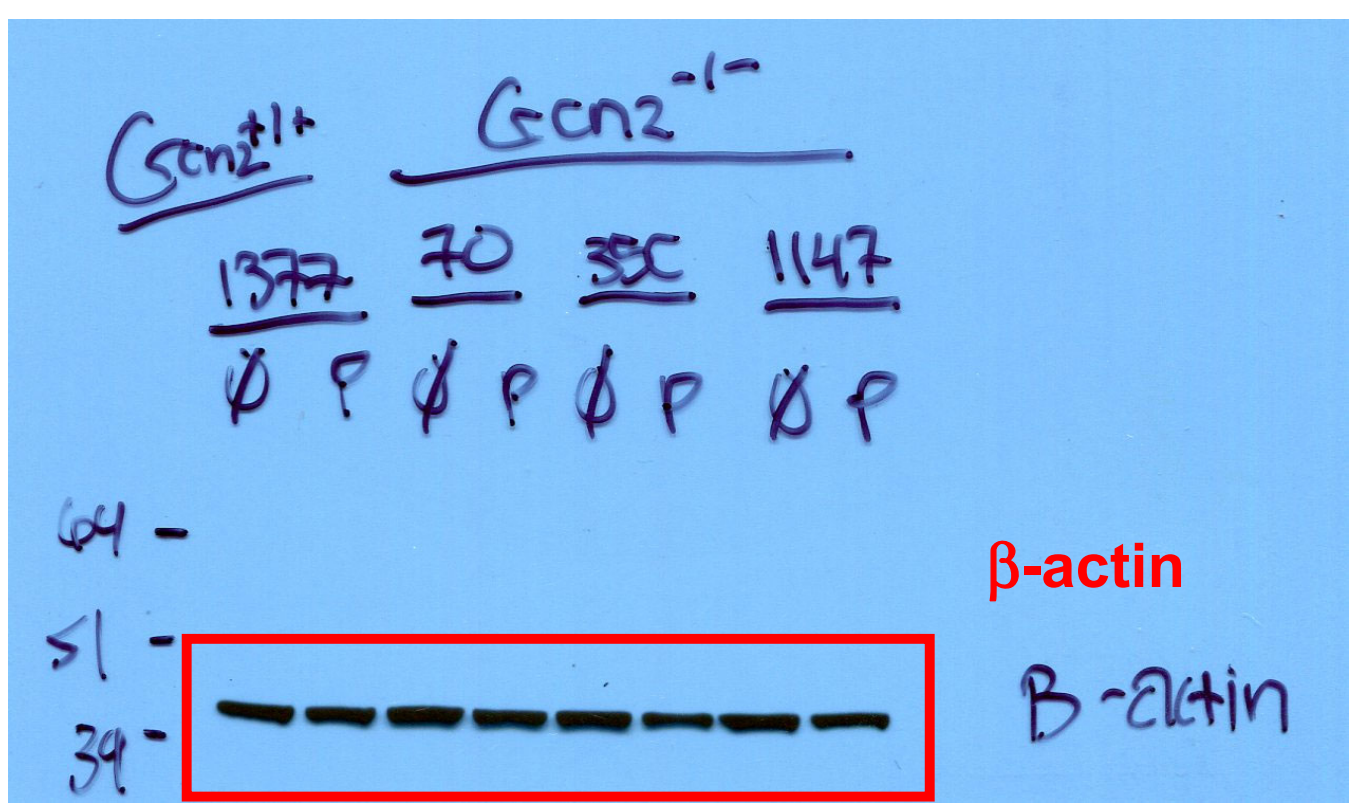

Figure 4F

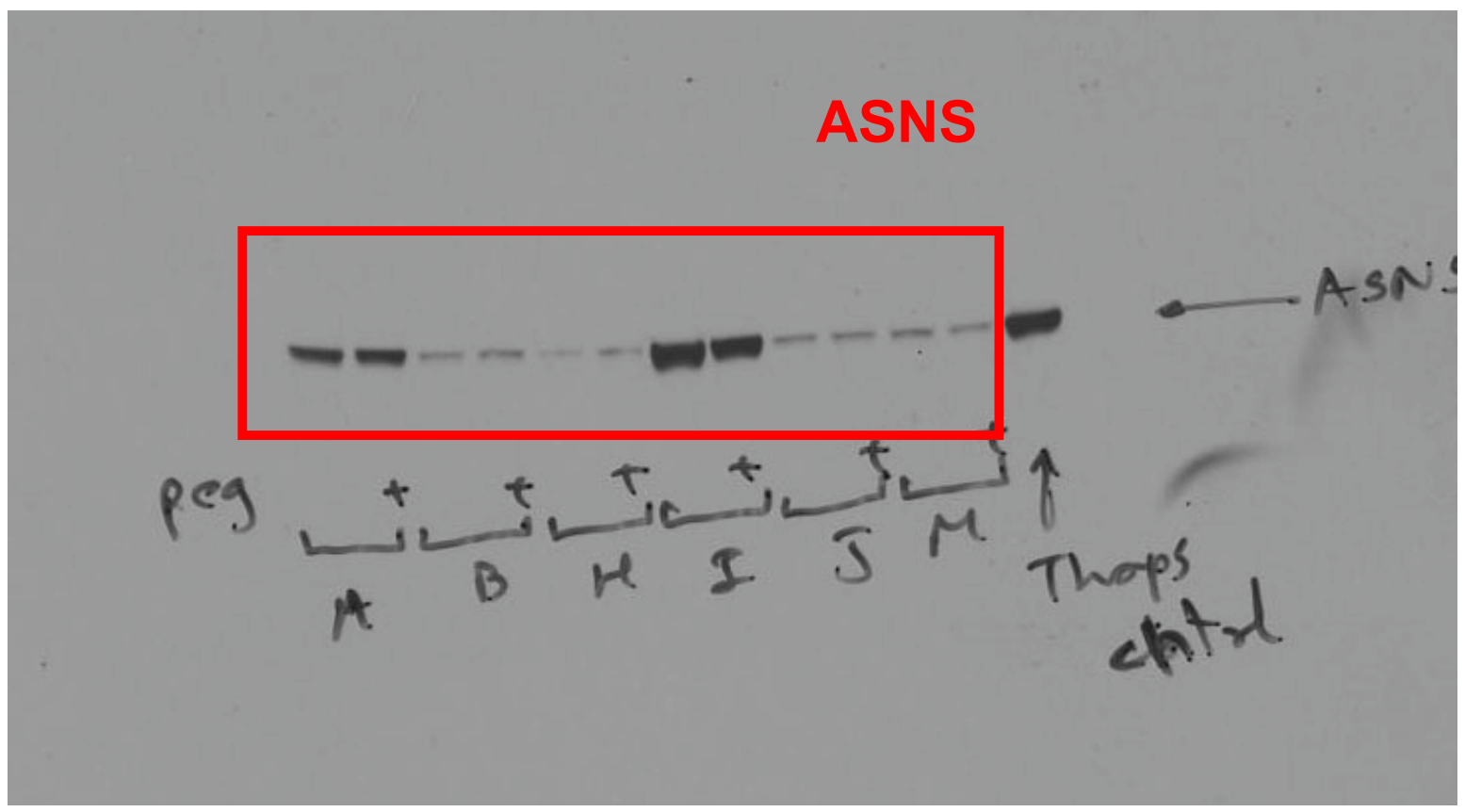

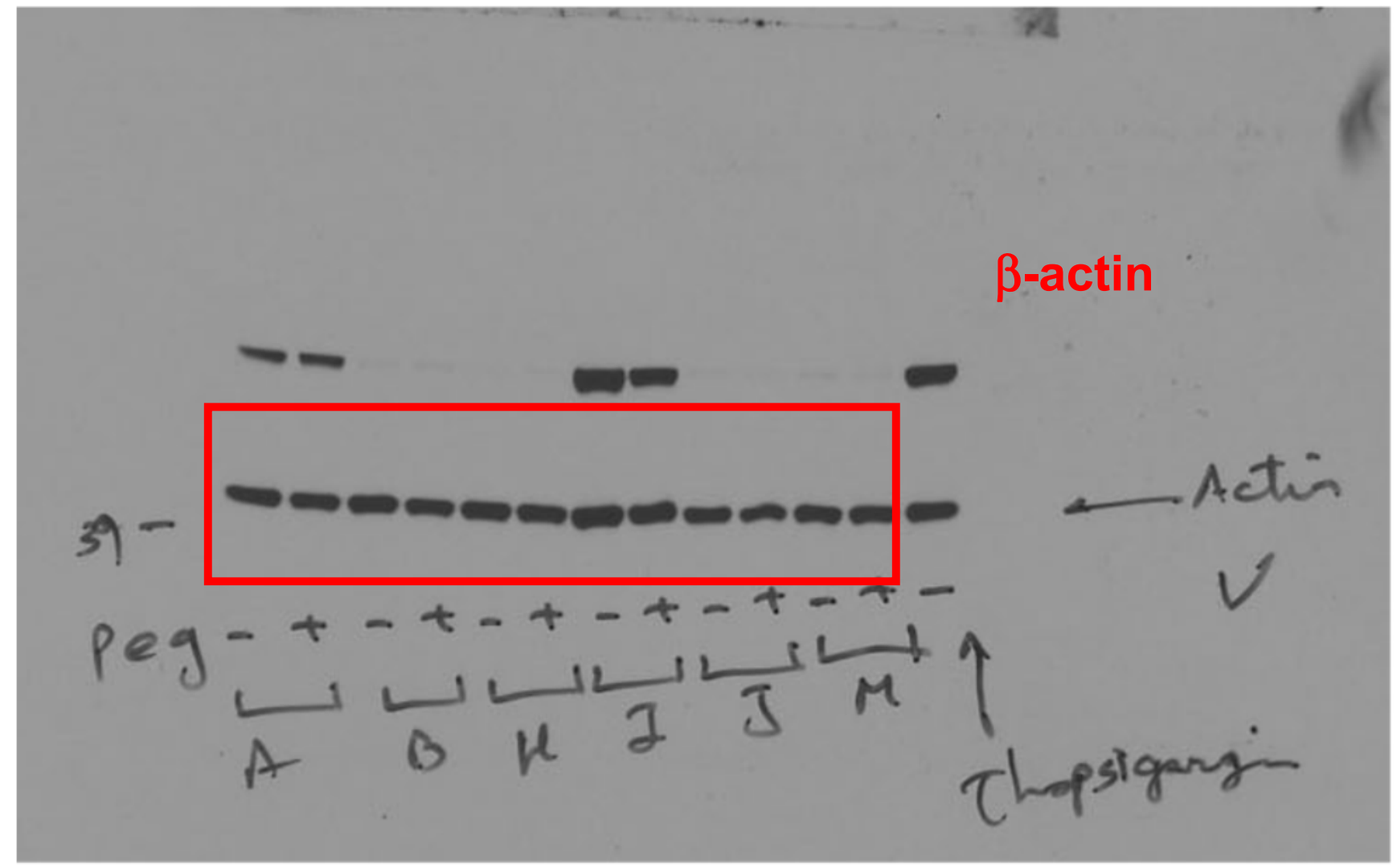

Figure 5A

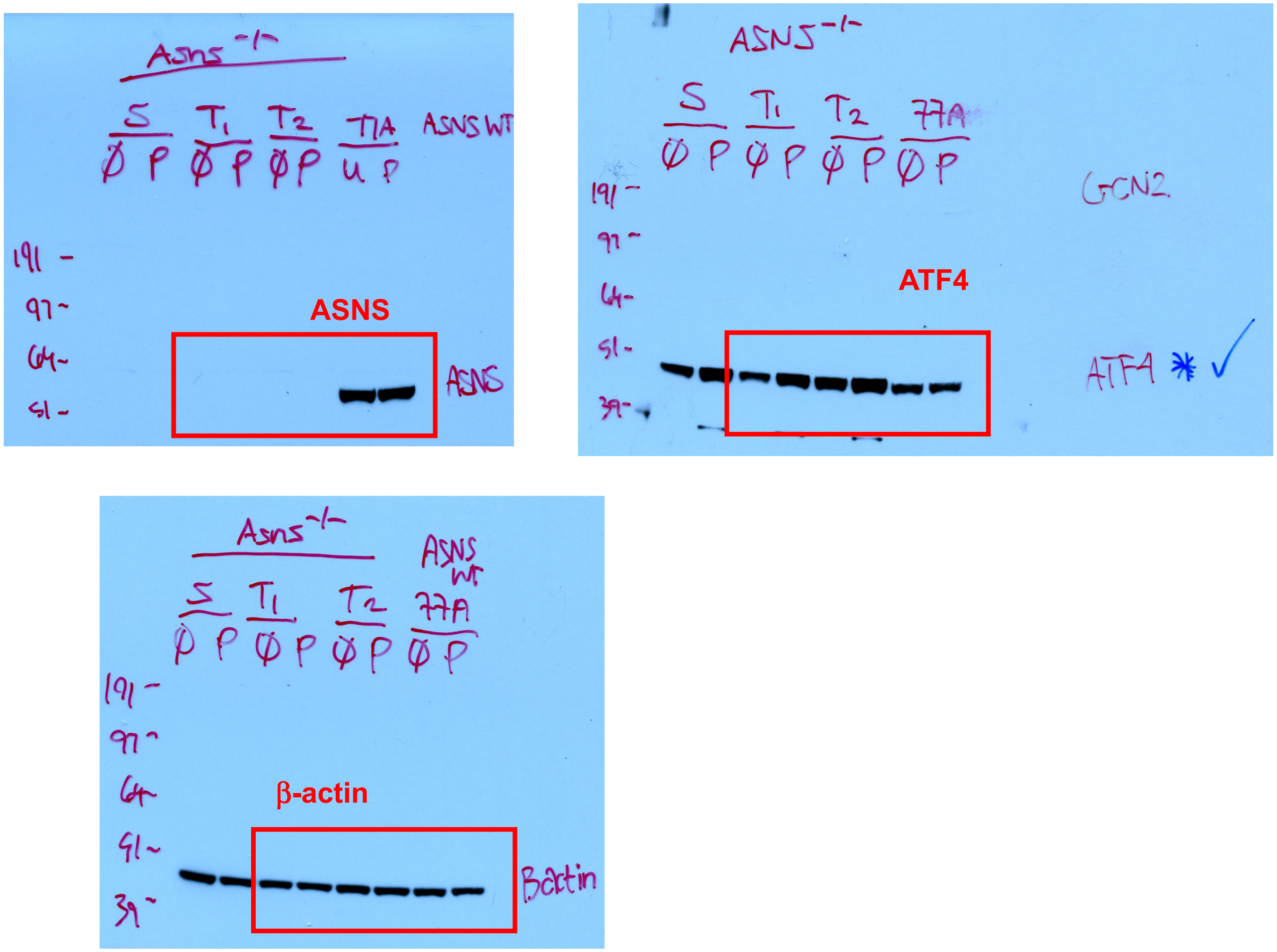
Asns -/-
S
T1
T2
77A
ASNS WT
191
97
64
51
ASNS
ASNS
ASNS -/-
GCN2
ATF4
ATF4
39
β-actin
Bactin

Figure 5C

Labeling of Nalm-6 and Reh was swapped

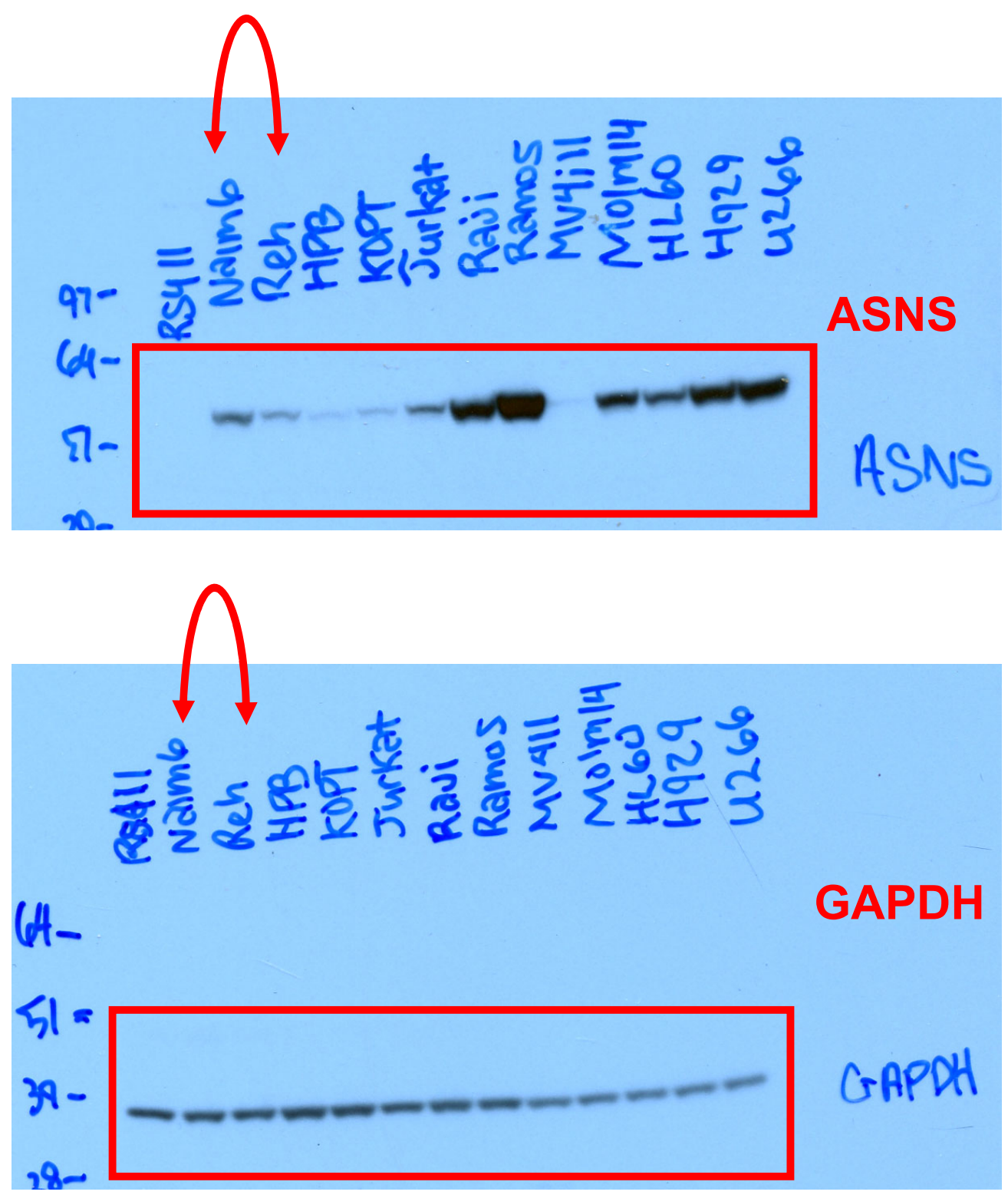

Figure 5H

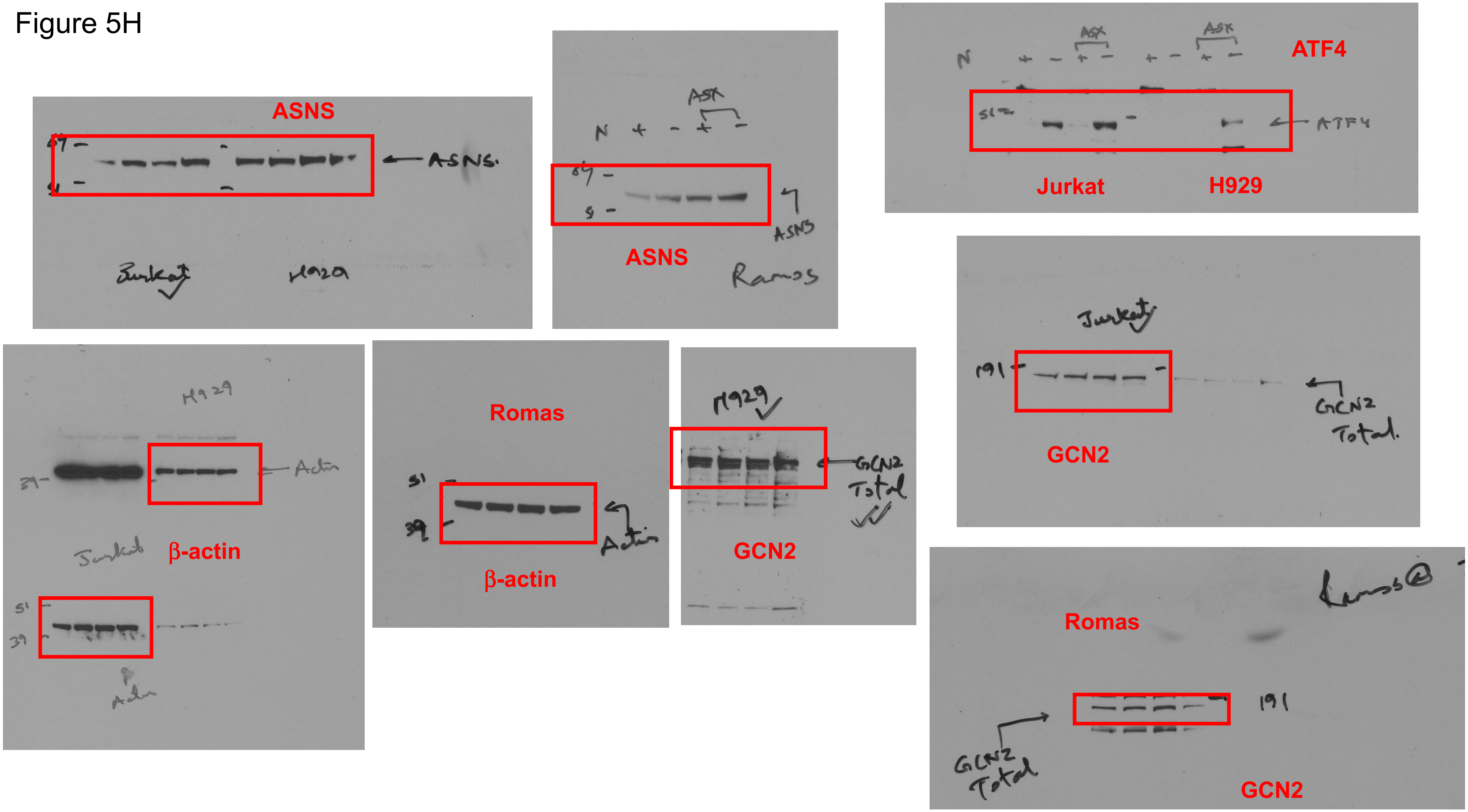
ASNS
ASNS
ATF4
Jurkat
H929
β-actin
Romas
β-actin
GCN2
GCN2
Romas
GCN2

Figure 5H

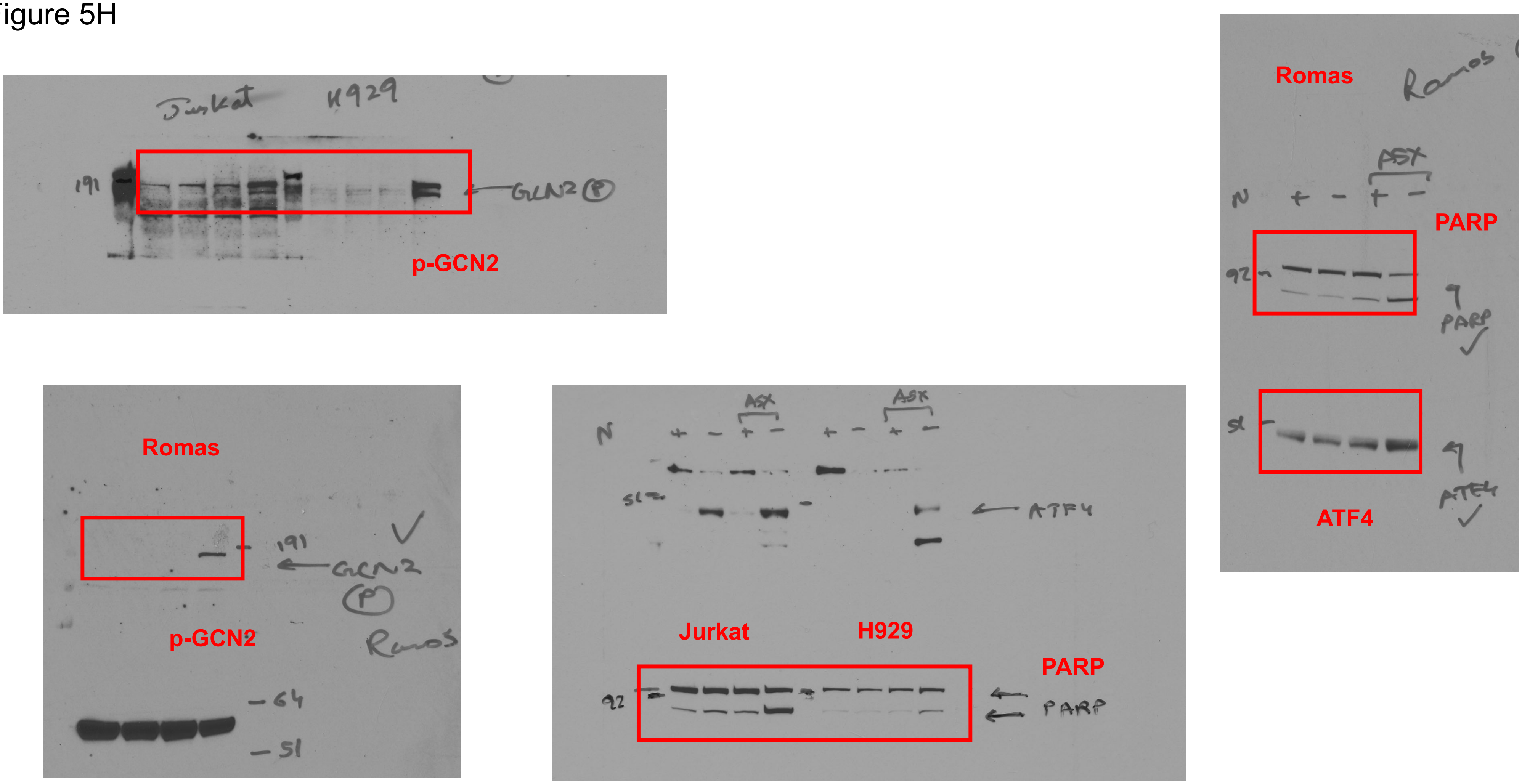

Jurkat
H929
191
GCN2 (P)
p-GCN2
Romas
191
GCN2 (P)
p-GCN2
Ramos
64
51
N
ASX
51
ATF4
Jurkat
H929
PARP
92
PARP
Romas
Ramos
ASX
N
PARP
92
PARP
51
ATF4
ATF4

Figure 6H

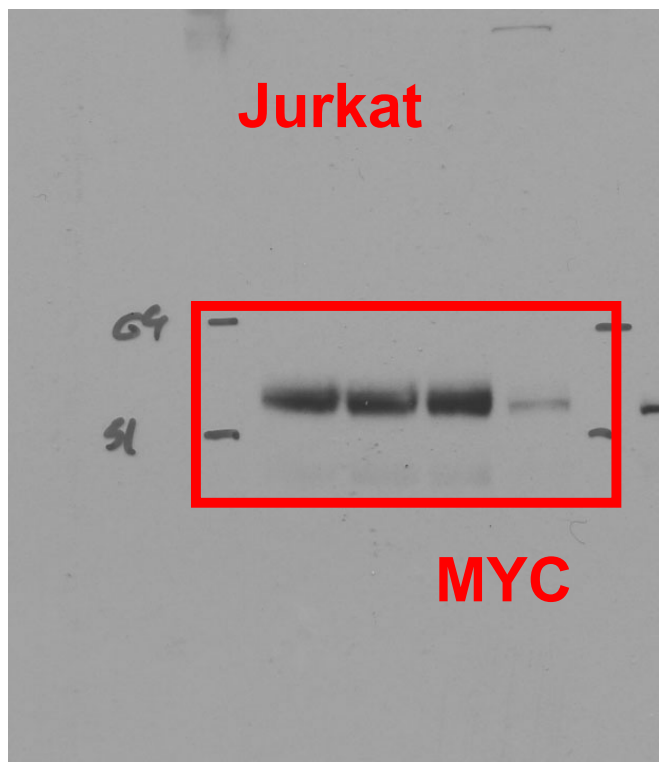
Jurkat
MYC

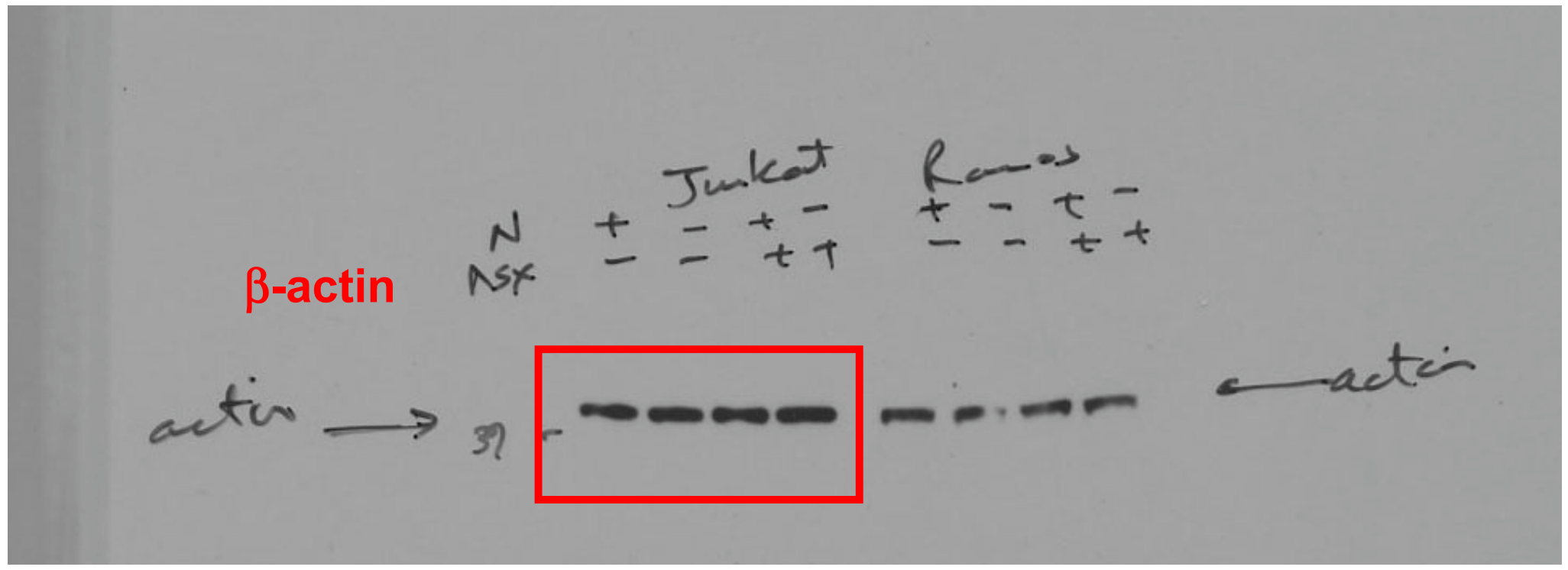
β-actin
Jurkat
N
ASX
actin
39

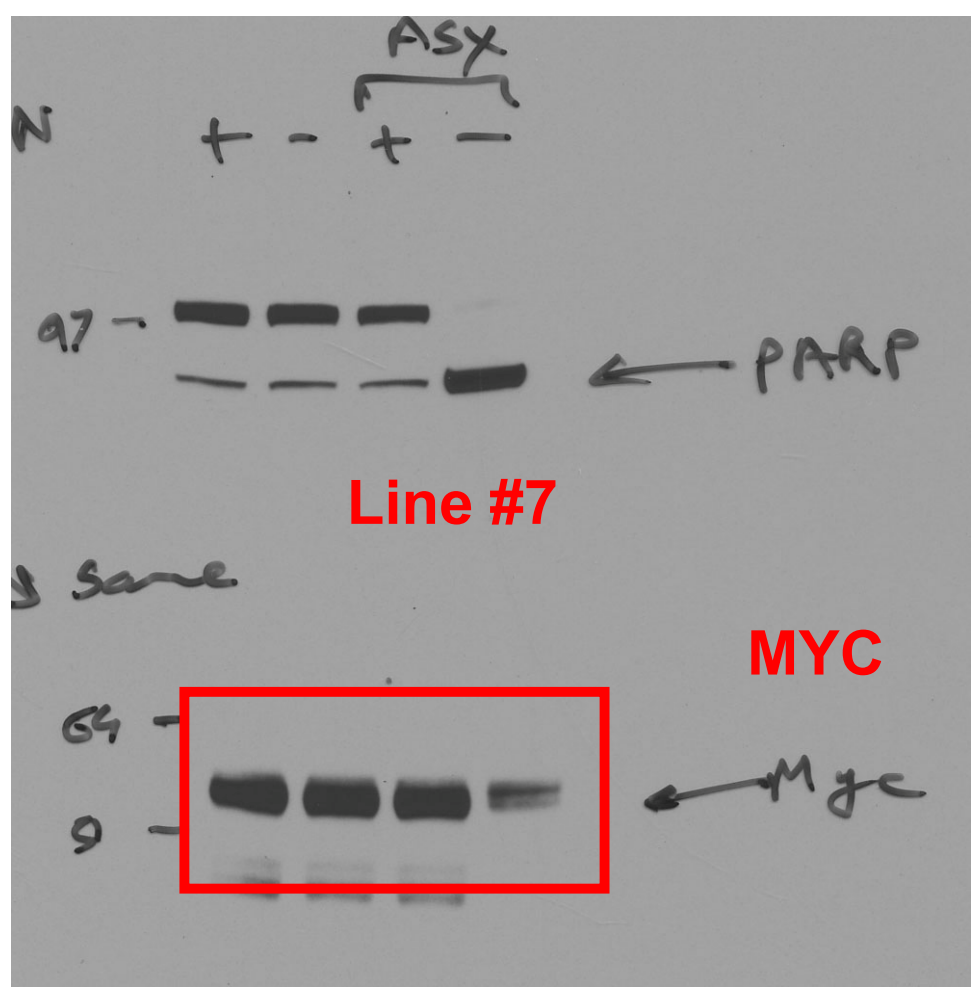
ASX
N
97
PARP
Line #7
MYC
64
Myc

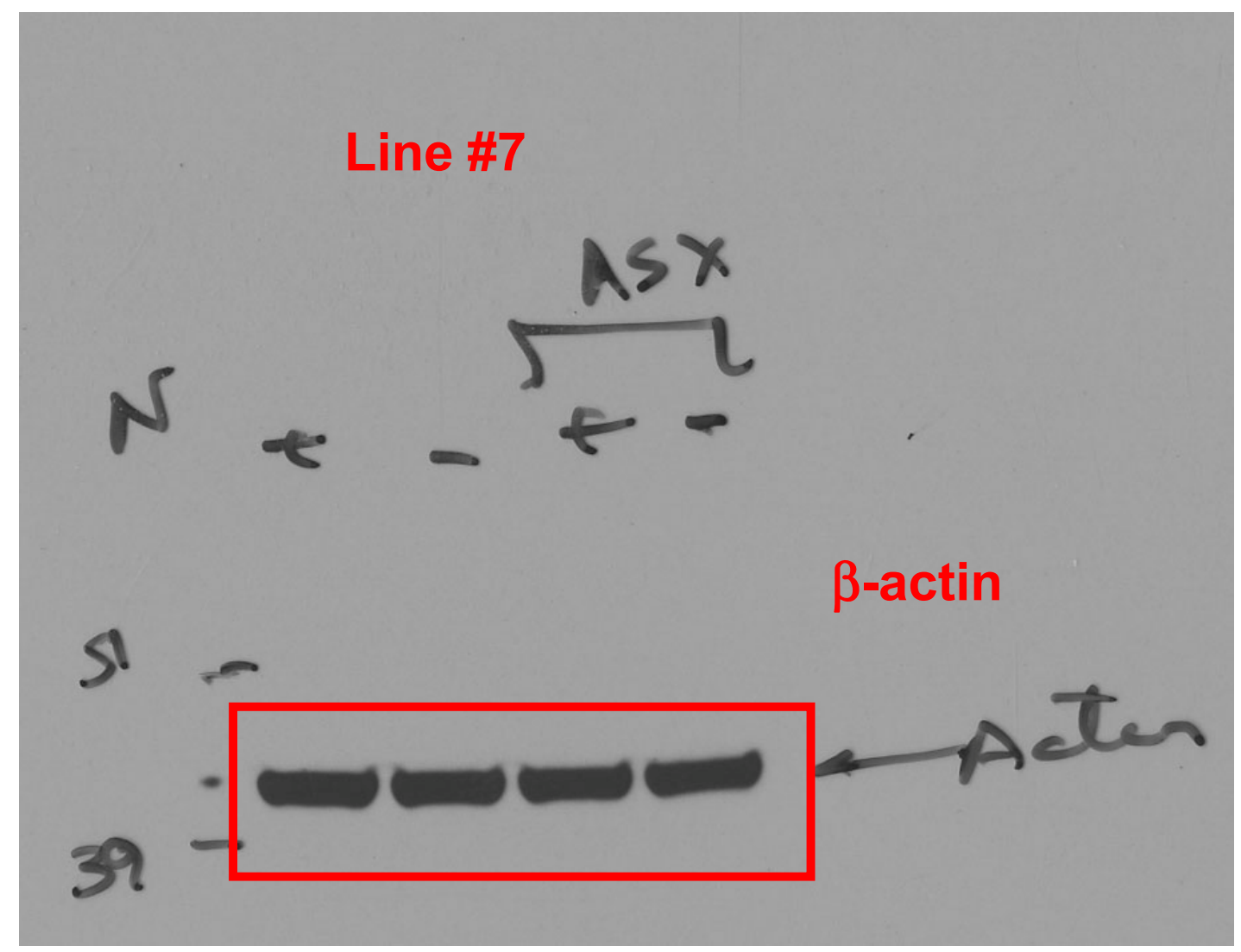
Line #7
ASX
N
β-actin
51
39
Actin

SFigure 1A

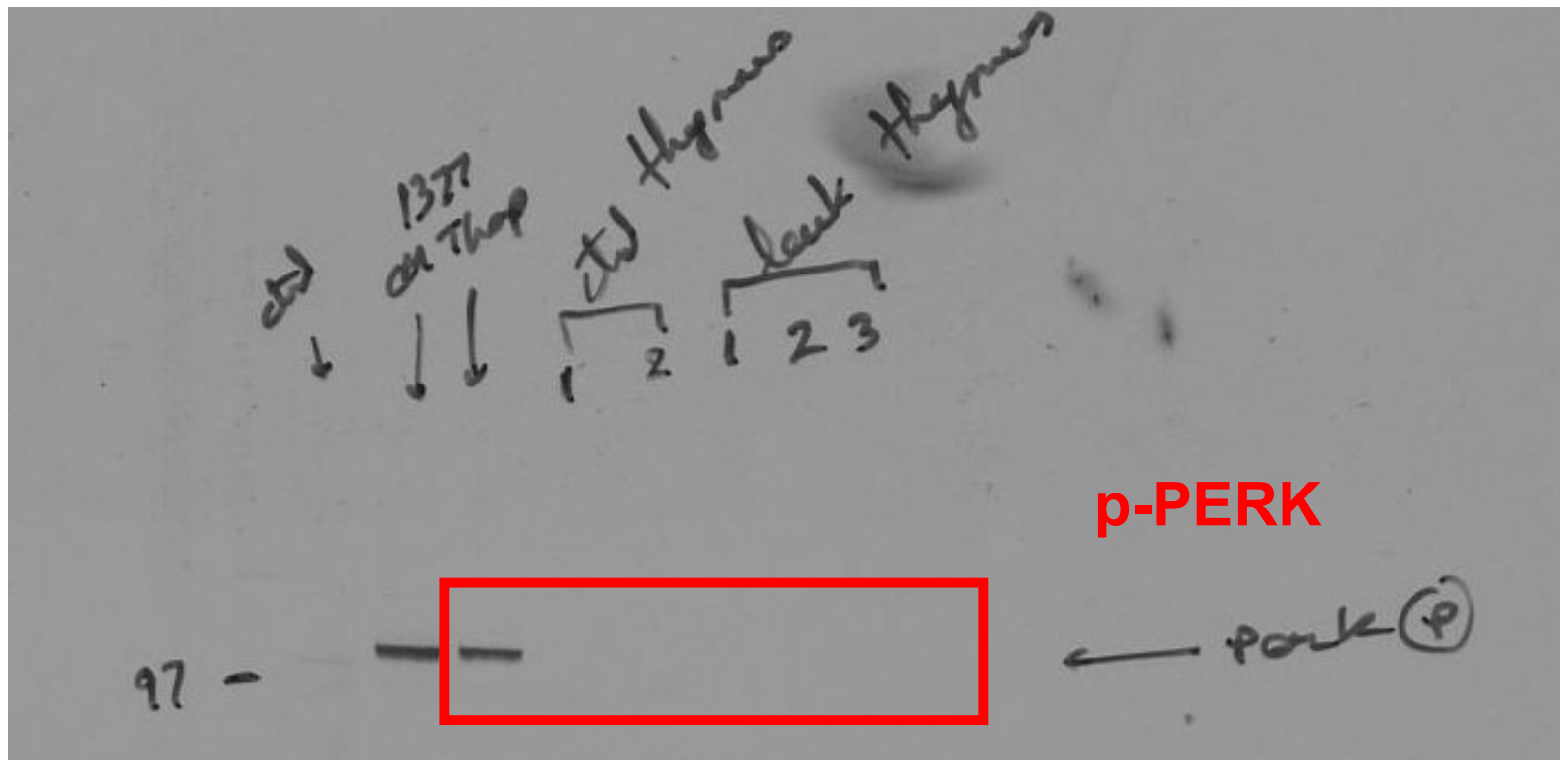

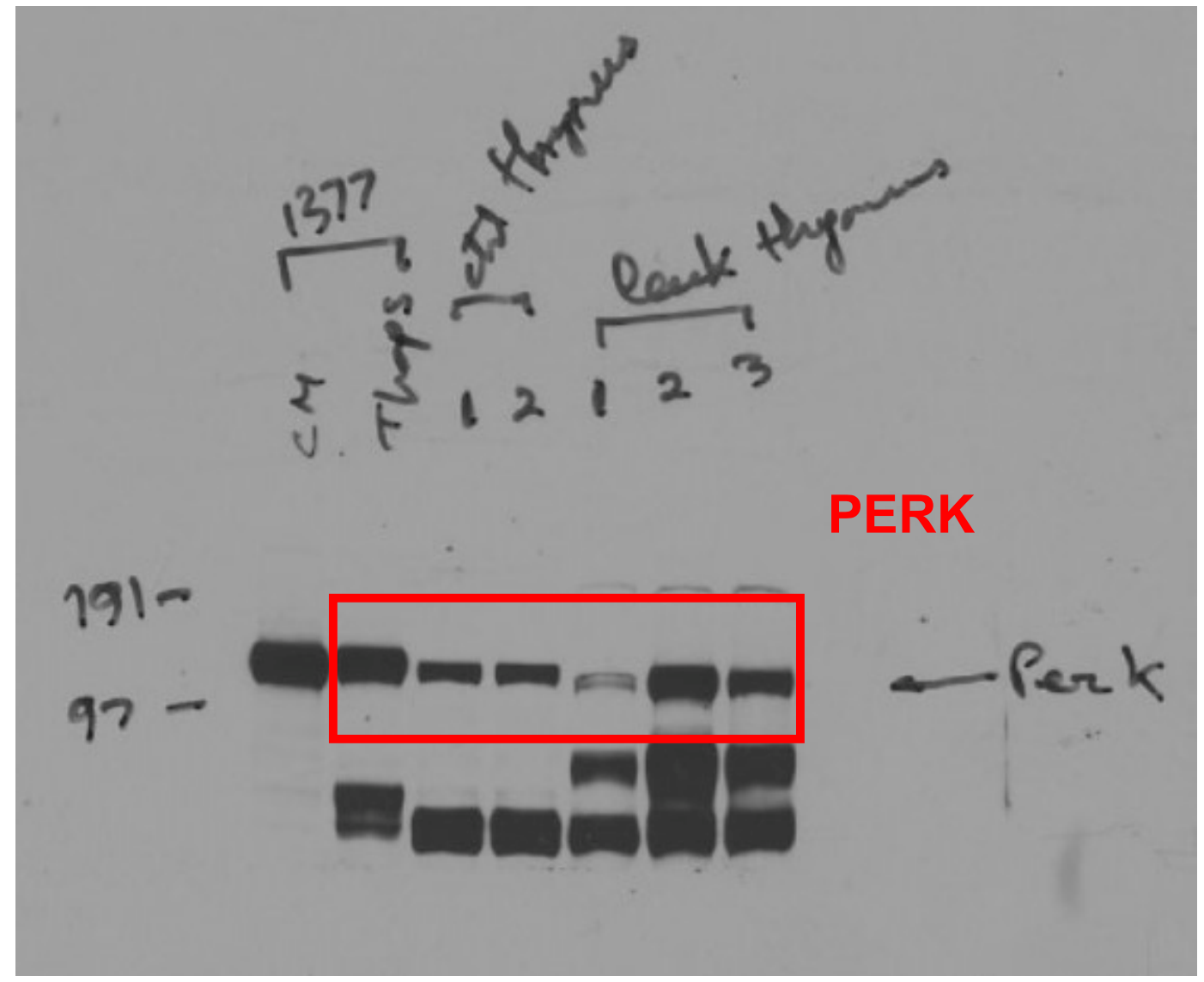

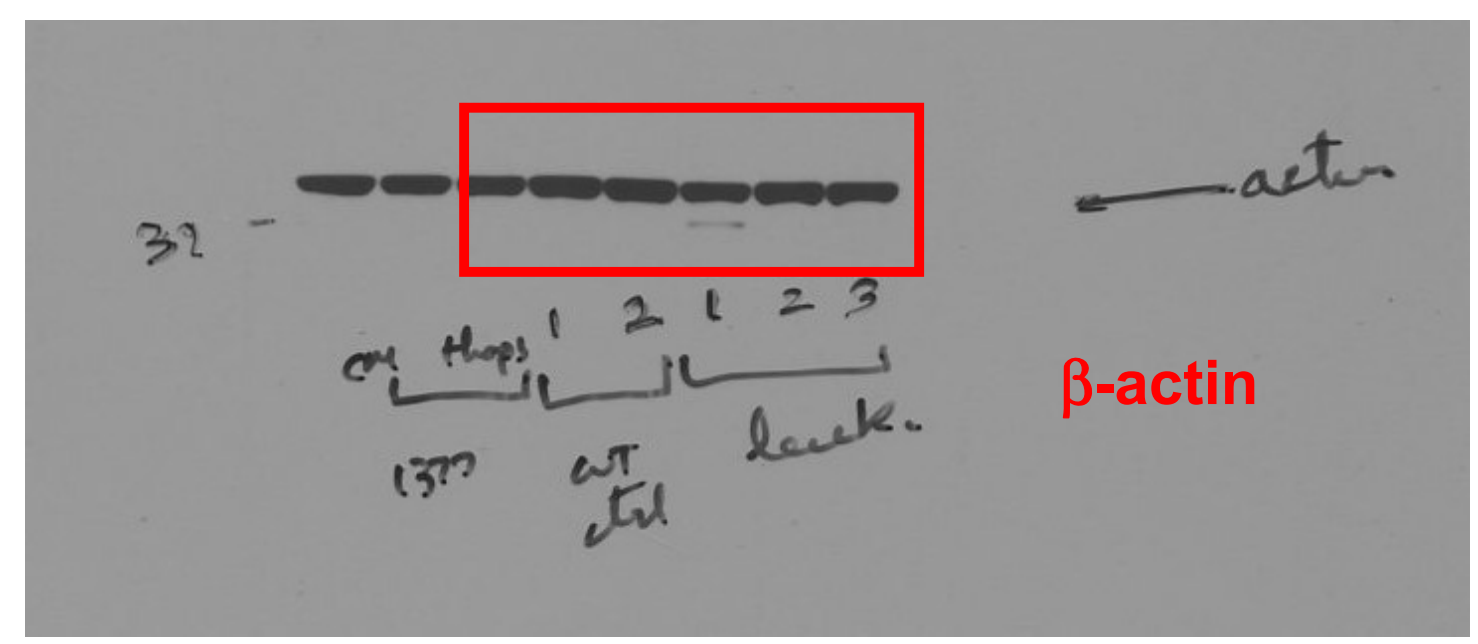

SFigure 1D

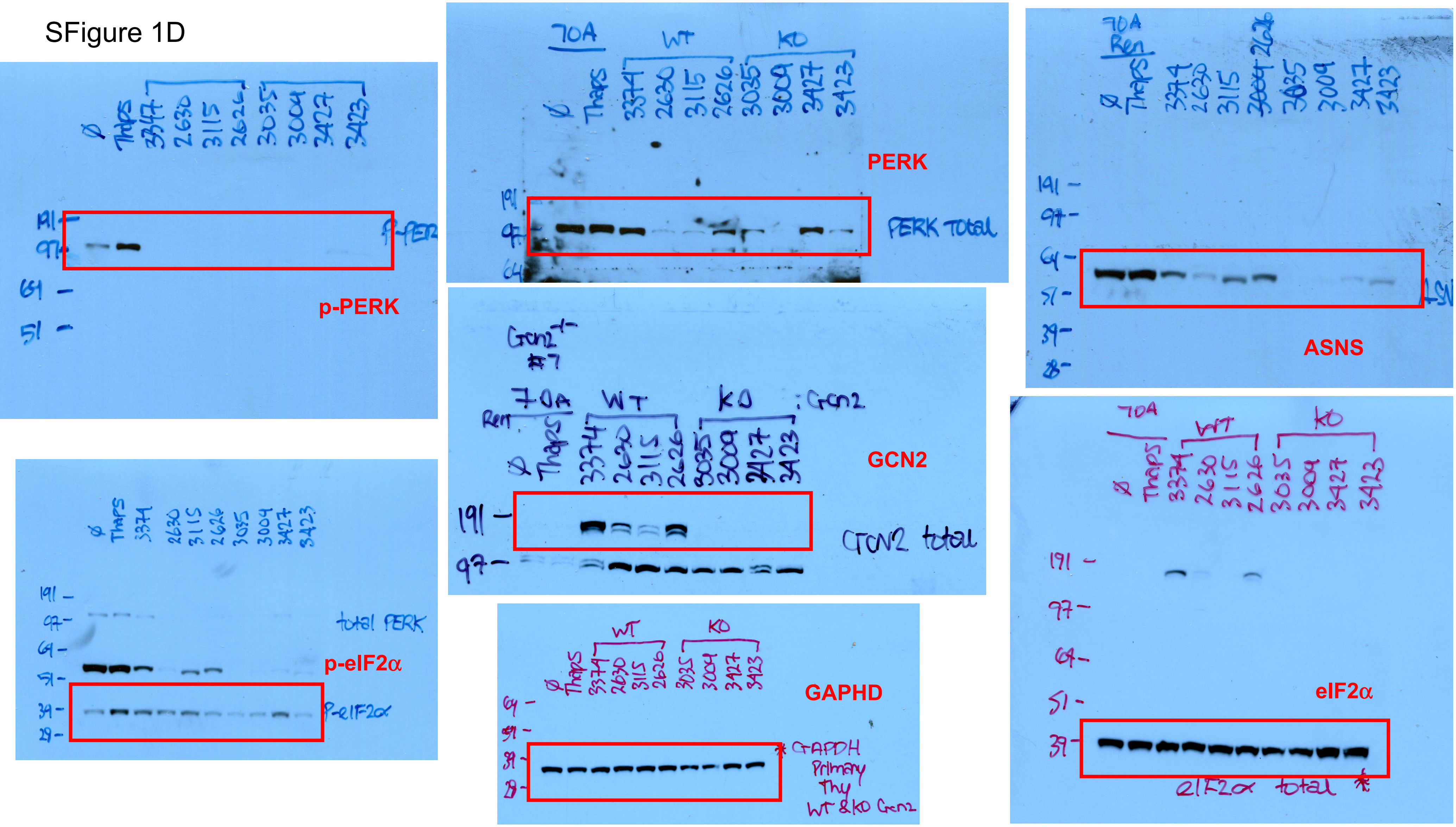
p-PERK
PERK
PERK Total
ASNS
GCN2
GCN2 total
p-eIF2α
total PERK
GAPHD
eIF2α
eIF2α total

SFigure 2A

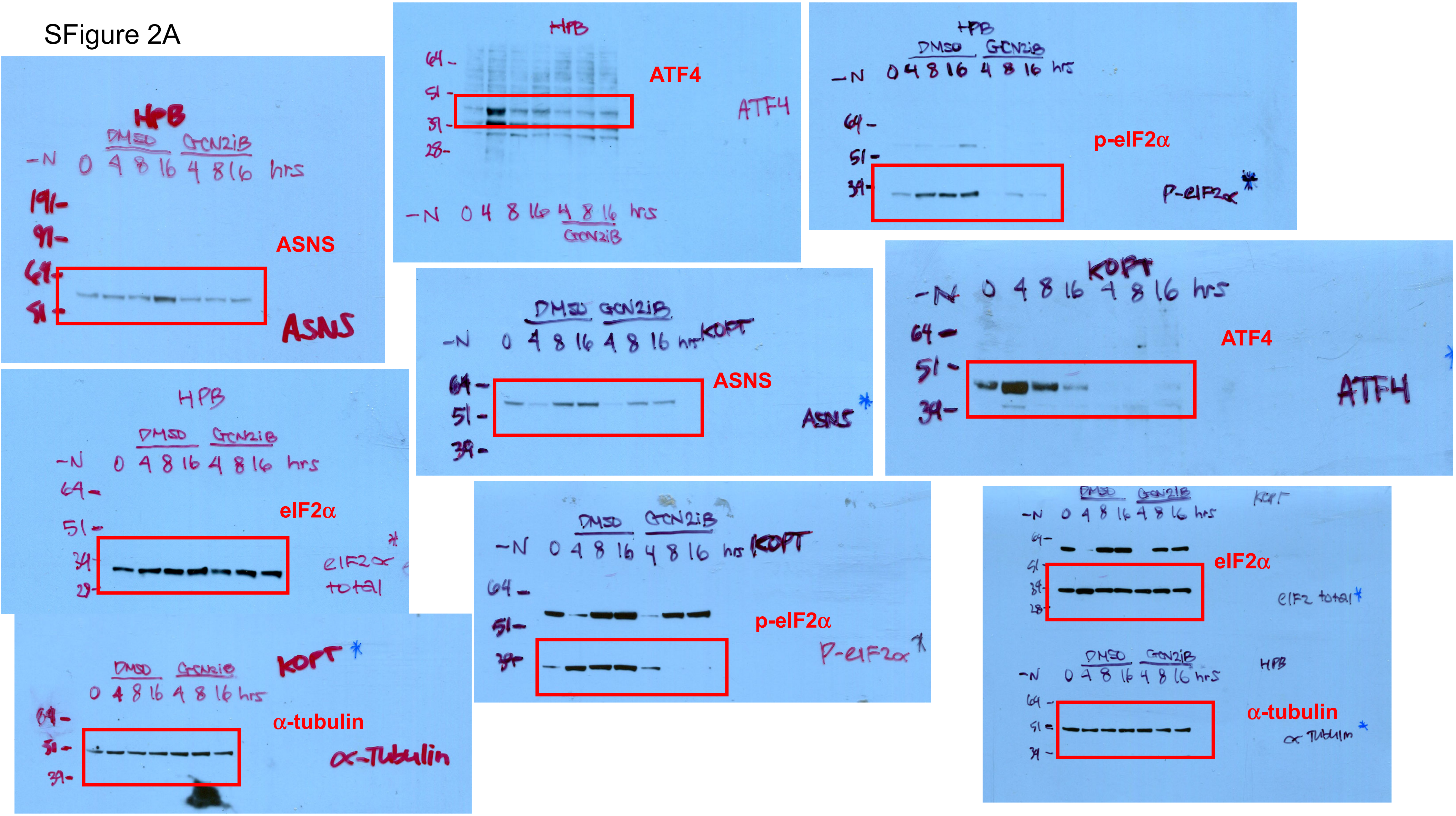
ATF4
p-eIF2α
ASNS
ASNS
ATF4
eIF2α
p-eIF2α
eIF2α
α-tubulin
α-tubulin

SFigure 3A

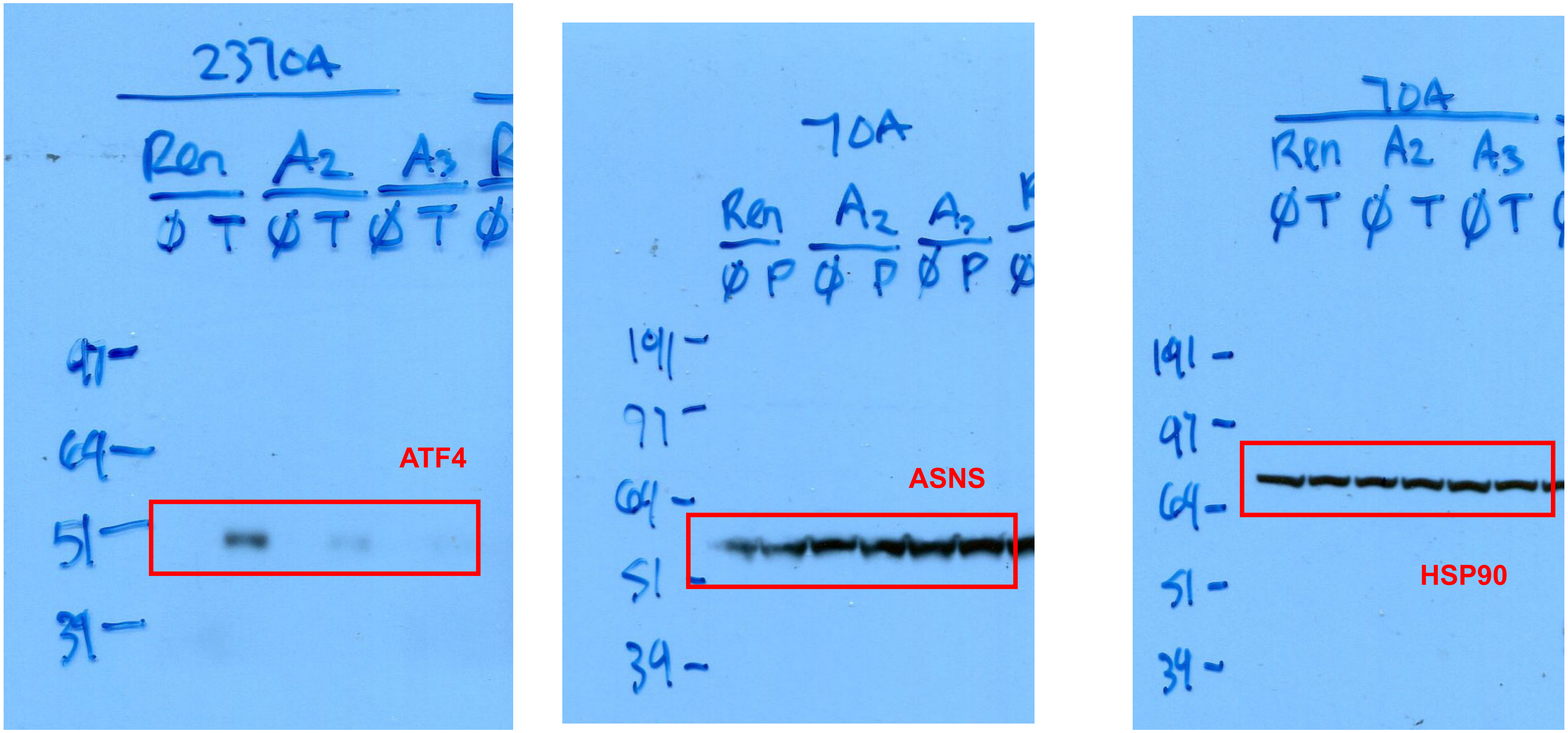

SFigure 3C

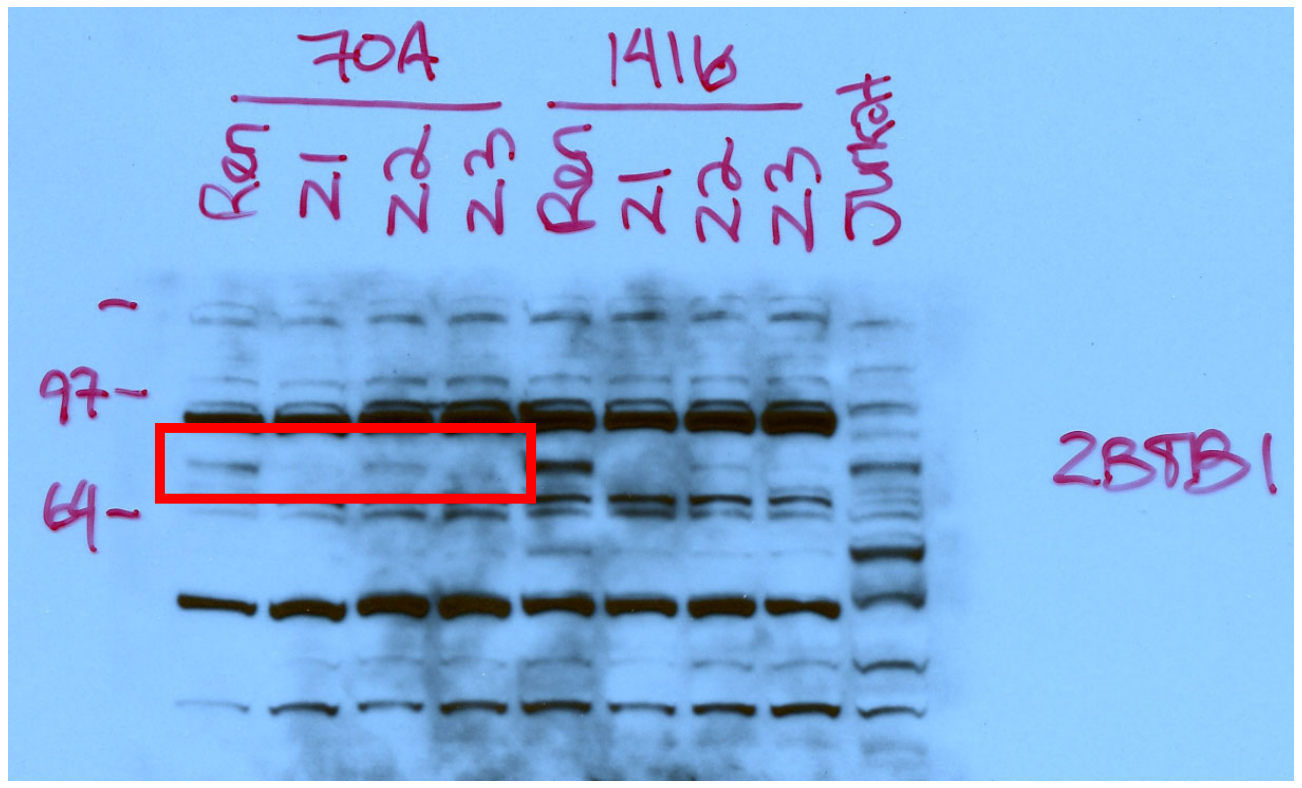

ZBTB1

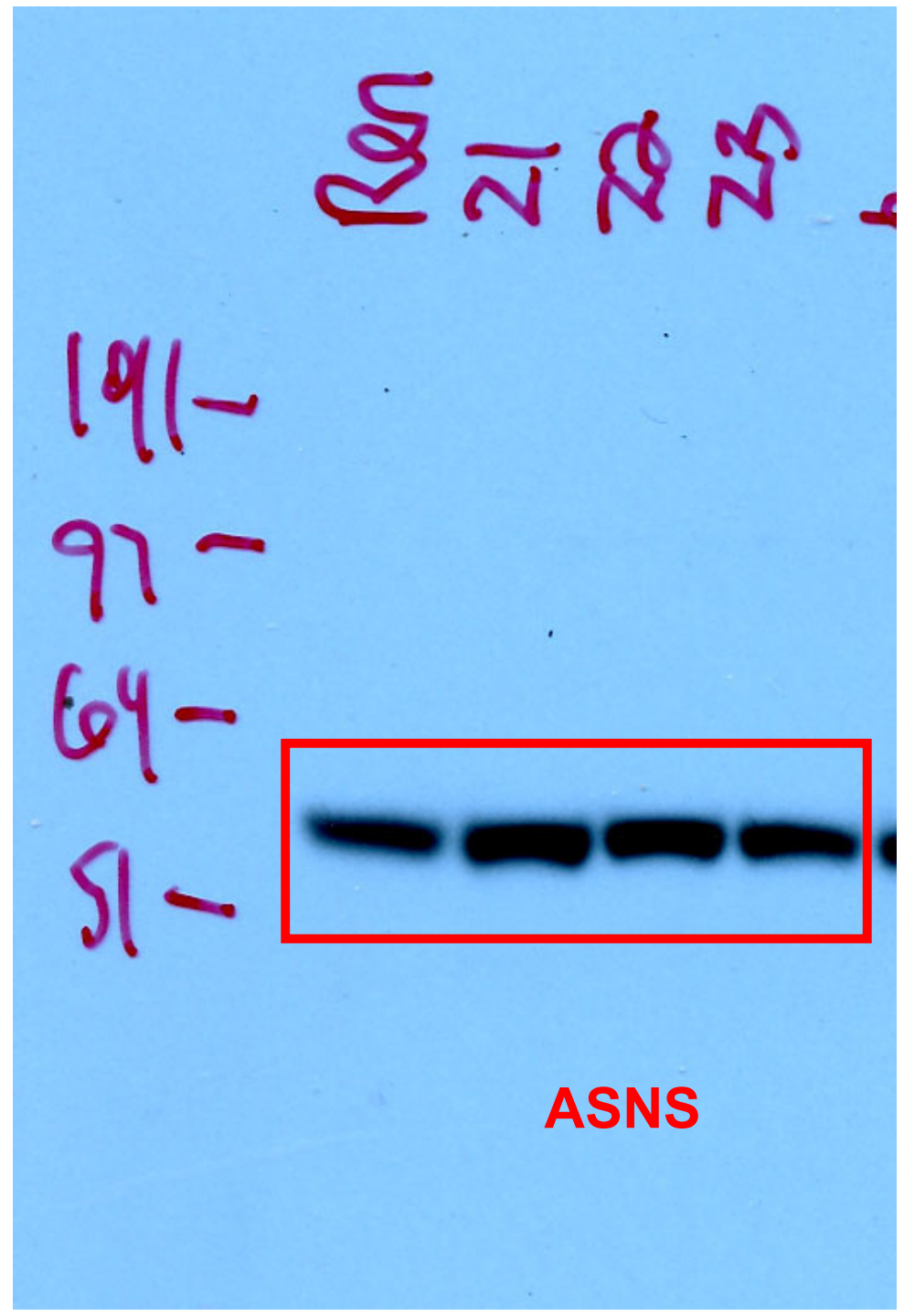

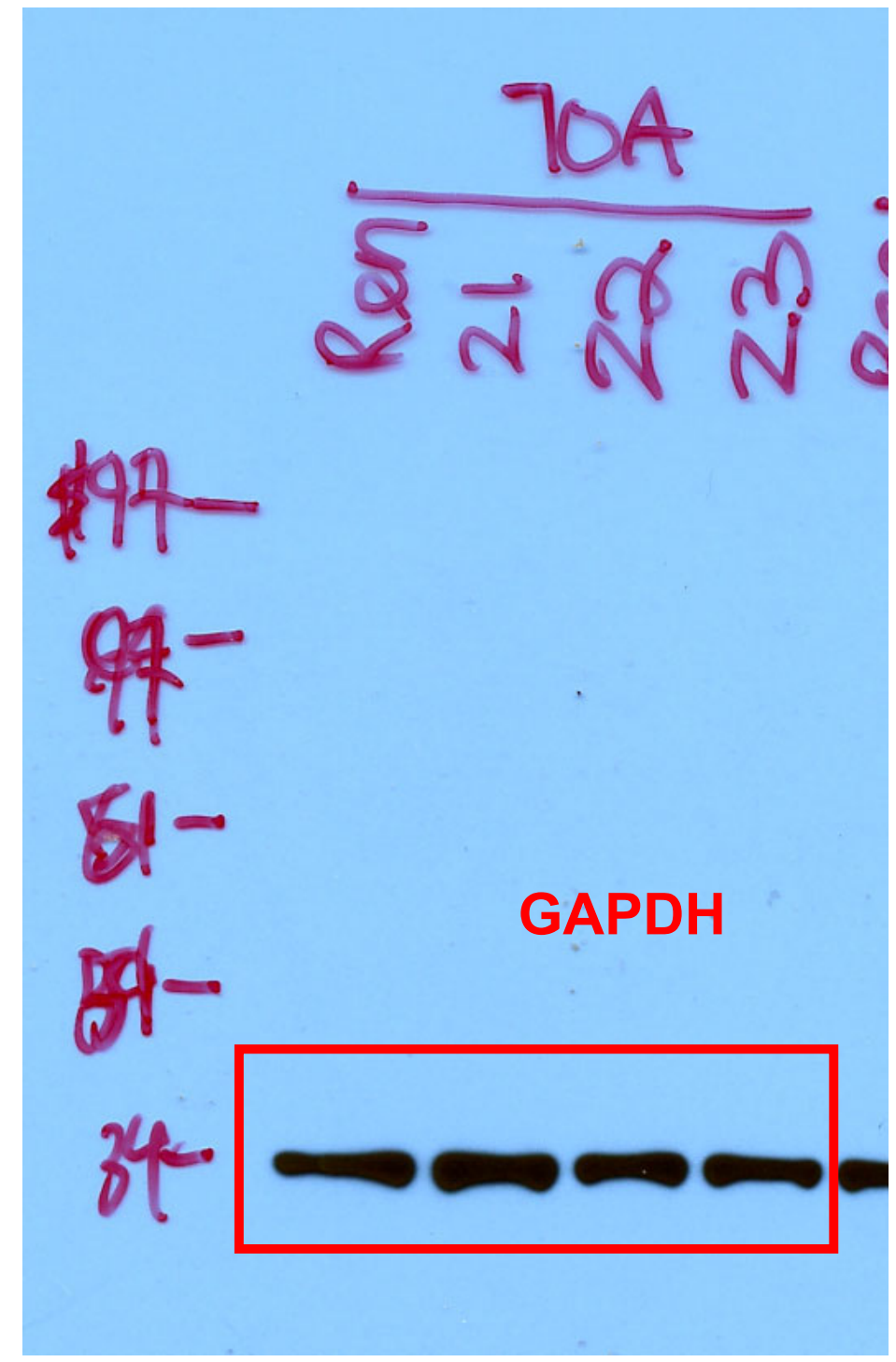

Supplement: Supplementary file 2 [file jciinsight-11-203777-s117.pdf]
